# Tsunami records of the last 8000 years in the Andaman Island, India, from mega and large earthquakes: Insights on recurrence interval

**DOI:** 10.1038/s41598-019-54750-6

**Published:** 2019-12-05

**Authors:** Javed N. Malik, Frango C. Johnson, Afzal Khan, Santiswarup Sahoo, Roohi Irshad, Debajyoti Paul, Shreya Arora, Pankaj Kumar Baghel, Sundeep Chopra

**Affiliations:** 1 Department of Earth Sciences, Indian Institute of Technology Kanpur, Kanpur, 208016 Uttar Pradesh India; 2 Department of Energy and Environment, Babasaheb Bhimrao Ambedkar University, Lucknow, 226025 Uttar Pradesh India; 3 Department of Geology, Utkal University, Vani Vihar, Bhubaneswar, 751004 Odisha India; 4 Inter- University Accelerator Centre (AMS & Pelletron Group), New Delhi, 110067 India

**Keywords:** Natural hazards, Tectonics

## Abstract

As many as seven tsunamis from the past 8000 years are evidenced by sand sheets that rest on buried wetland soils at Badabalu, southern Andaman Island, along northern part of the fault rupture of the giant 2004 Aceh-Andaman earthquake. The uppermost of these deposits represents the 2004 tsunami. Underlying deposits likely correspond to historical tsunamis of 1881, 1762, and 1679 CE, and provide evidence for prehistoric tsunamis in 1300–1400 CE, in 2000–3000 and 3020–1780 BCE, and before 5600–5300 BCE. The sequence includes an unexplained hiatus of two or three millennia ending around 1400 CE, which could be attributed to accelerated erosion due to Relative Sea-Level (RSL) fall at ~3500 BP. A tsunami in 1300–1400, comparable to the one in 2004, was previously identified geologically on other Indian Ocean shores. The tsunamis assigned to 1679, 1762, and 1881, by contrast, were more nearly confined to the northeast Indian Ocean. Sources have not been determined for the three earliest of the inferred tsunamis. We suggest a recurrence of 420–750 years for mega-earthquakes having different source, and a shorter interval of 80–120 years for large magnitude earthquakes.

## Introduction

**Figure 1:**
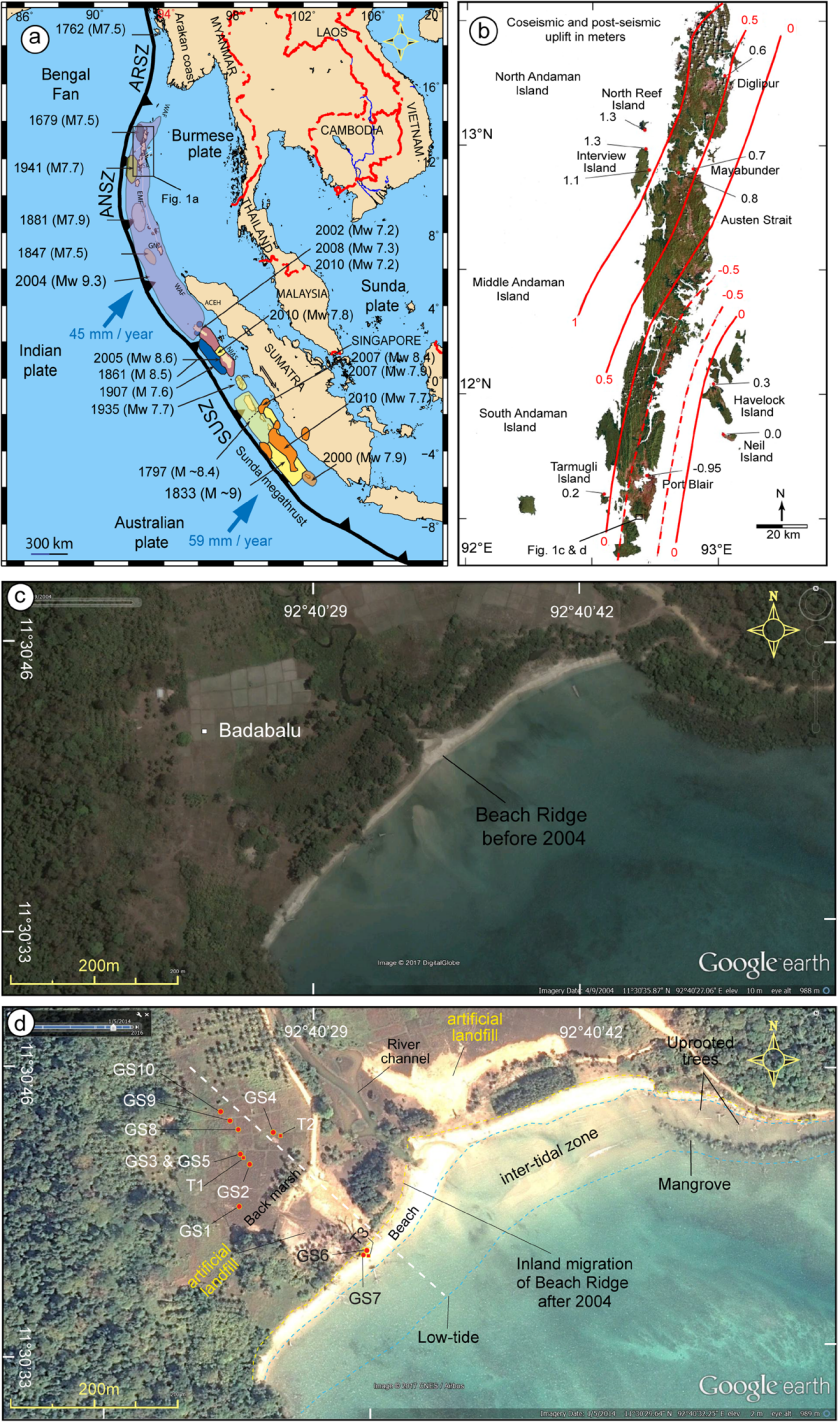
**(a)** Regional tectonic map of Sumatra-Andaman region along with the extent of ruptures and earthquakes (with magnitude) occurring between AD1600–2004. The map was generated using Generic Mapping Tool (GMT). Fault lines, earthquake rupture areas, earthquakes listed with magnitude are adopted from Malik *et al*.^3^; Monecke *et al*.^3^; Meltzner *et al*.^10,12^. Sumatra Subduction Zone (SUSZ), Andaman Subduction Zone (ANSZ) and Arakan Subduction Zone (ARSZ). Black box shows the area of Andaman Island of Fig. 1b. (**b**) Map of Andaman Islands showing areas of uplift and subsidence triggered by the 2004 event (after Malik *et al*.^3^). Black box shows the location of (**c**,**d**). (**c**) Google image of the area around Badabalu along the south coast of Andaman showing intact beach, beach-ridge and back-marsh before the 2004 event. The image was taken on 09 April 2004 (Map data: *Google, DigitalGlobe*). (**d**) Google image showing the effect of the 2004 Sumatra-Andaman earthquake. The land subsidence was ~40–45 cm, resulted in inundation and erosion of coastline, as well as inland migration of beach, beach-ridge and back-marsh. The image was taken on 01 May 2014 (Map data: *Google, CNES/Airbus*). White dashed line marks the WNW-ESE striking transect along which topographic profile, as well as stratigraphic sections, were collected. Geoslice locations are indicated as GS1 to GS10 and trenches as T1 to T3.

The societal impact from tsunamis is extremely catastrophic. Two recent tsunamis triggered by 2004 Sumatra-Andaman (Mw 9.3) and 2011 Tohoku (Mw 9.1) mega- earthquakes along subduction zones have exhibited our poor understanding about their occurrences, characteristic wave patterns, and sediment transport^1–3^. Reliable information pertaining to such events, which can be obtained from geological records, is crucial to minimize the consequent disaster. The Andaman and Nicobar Islands, and coastal areas along the Mainland India (as well as Indonesia and Thailand) are vulnerable to tsunamis generated from earthquakes originating from different sources that exist along the Sumatra Subduction Zone (SUSZ), Andaman Subduction Zone (ANSZ) and Arakan Subduction Zone (ARSZ) (Fig. 1a). Therefore, these are the best locations to study detailed aspects of tsunami events.

The 26 December 2004 tsunamigenic earthquake took more than 280,000 lives. A large coseismic rupture of ~1300 km^4–6^ occurred along the SUSZ and ANSZ, and resulted in dramatic land-level changes marked by prominent uplift and subsidence along the west and east coast of Andaman and Nicobar Islands (Fig. 1a,b)^3,6,7^. Historical earthquakes preceded the 2004 earthquake in 1679, 1762, 1847, 1881, and 1941 (?) (Fig. 1a). Lack of comprehensive historical data poses a big challenge for a proper tsunami hazard evaluation for the Andaman and Nicobar Islands as well as the east coast of Mainland India. A poor understanding of such catastrophic events implies a huge risk associated with the failure of existing/upcoming nuclear power plants and life-line infrastructure near the densely populated coastal areas in India. Also, lesson learnt from the Tohoku earthquake and associated tsunami necessitates identification of tsunamis generated from local source(s) along the Andaman-Arakan or Sumatra segment. Even earthquakes with large magnitude should not be underestimated, which could be devastative as experienced during the recent 28 September 2018, Palu tsunami in Indonesia, produced by a Mw7.5 earthquake.

Barring a few studies, not much information on paleoseismic and paleo-tsunami is available^3,6–13^. A recent study based on turbidites reveals 6600 year earthquake history from the Sumatra-Andaman Subduction Zone^14^. In addition to the 2004 Sumatra-Andaman earthquake (Mw 9.3) and the associated giant tsunami, several other tsunami events occurred during the medieval period CE 850–900, CE 660–880, CE 1100–1300, CE 1679, and CE 1762^3^. Of these, the CE 660–880 and CE 1100–1300 were unusual events (mega earthquakes 9.0 ≥ Mw ≤ 9.5) triggered along the Andaman-Arakan and Andaman segments respectively, and generated transoceanic tsunamis^3^. These events were similar to the 2004 Sumatra-Andaman earthquake in terms of magnitude, rupture length and in producing giant tsunami. In this study, we present geological signatures of at least seven tsunamis over the past 8000 years obtained from shallow stratigraphic sections retrieved from 10 geoslices and 3 trenches at Badabalu, along the south coast of Andaman Island (Fig. 1b–d). A compilation of all available paleoseismic and paleo-tsunami events reported from Andaman and Nicobar Islands and areas adjoining the Indian Ocean like Burma, Thailand, Indonesia and Sri Lanka, are summarized in Supplementary Table S1 ^3–17^.

The ENE-WSW striking Badabalu beach along the south coast of Andaman is a famous tourist destination (Fig. 1b,c). The Badabalu area was severely affected by the 2004 tsunami (Sumatra-Andaman earthquake) and experienced a coseismic subsidence of ~40–45 cm (Supplementary Fig. S1.1a–h). Google Earth images from 2004 to 2014 clearly exhibit the pre- and post-seismic changes in coastal geomorphology (Fig. 1c,d, Supplementary Figs. S1.1a–h and S1.2a,b).

According to the survivors, the Badabalu area experienced ~4 m high tsunami waves, with run-up up to 0.8–1.0 km from the coast. Coseismic subsidence resulted into a landward shifting of the beach by 35–50 m (Supplementary Figs. S1.1a–h and S1.2a,b). Landward migration of the beach also caused inundation of the area and formation of beach ridge, back-marsh inland, and dead forest along the coastline. Local residents artificially filled the area by 0.7–0.8 m, shifted their houses to the higher ground, and elevated the coastal roads to avoid inundation around residential area and agricultural fields during high-tides (Supplementary Figs. S1.2–S1.4).

## Results

### Stratigraphy

**Figure 2:**
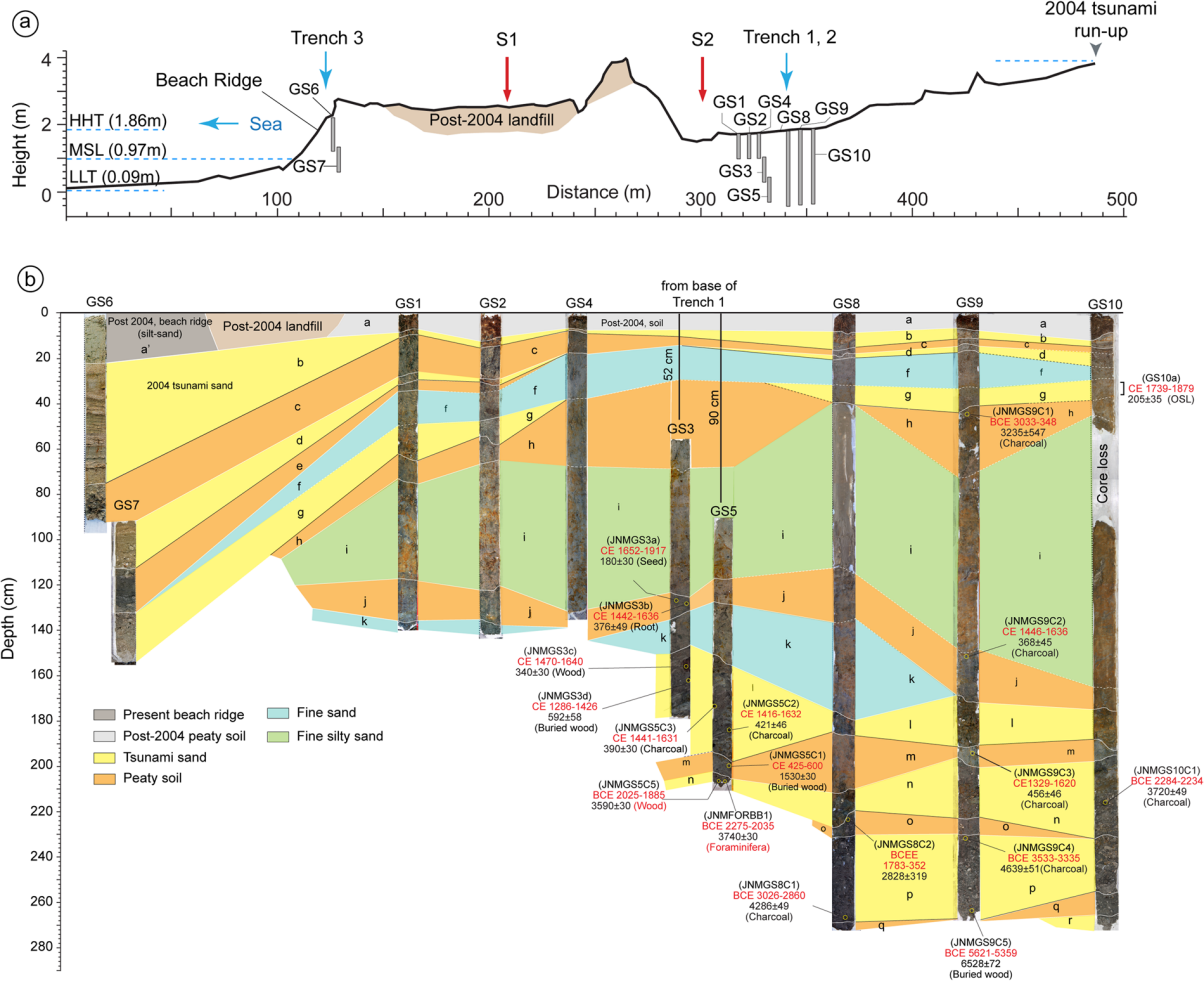
(**a**) Topographic profile collected along WNW-ESE transect (refer Fig. 1d for location). Locations of geoslices and trenches for shallow stratigraphic record are marked along the profile. The area shows beach ridge-swale-beach ridge topography. The middle portion of the profile shows artificial fill by local residents to prevent inundation. LLT – lower low tide, MSL – mean sea level and HHT – higher high tide. (**b**) Geoslice and trench sections placed with respect to horizontal. The vertical scale represents depth from the surface. The distribution of all exposed sedimentary lithounits was correlated. Based on the sedimentary characteristics the exposed units were classified into total 19 units, from the youngest Unit-a’ to the oldest Unit-r. In total seven tsunami deposits (including 2004 tsunami) were identified from the exposed stratigraphic sequence ranging in depth from 160–270 cm. Units b, d, g, l, n, p and r represents tsunami deposits marked by yellow colour.

**Figure 3:**
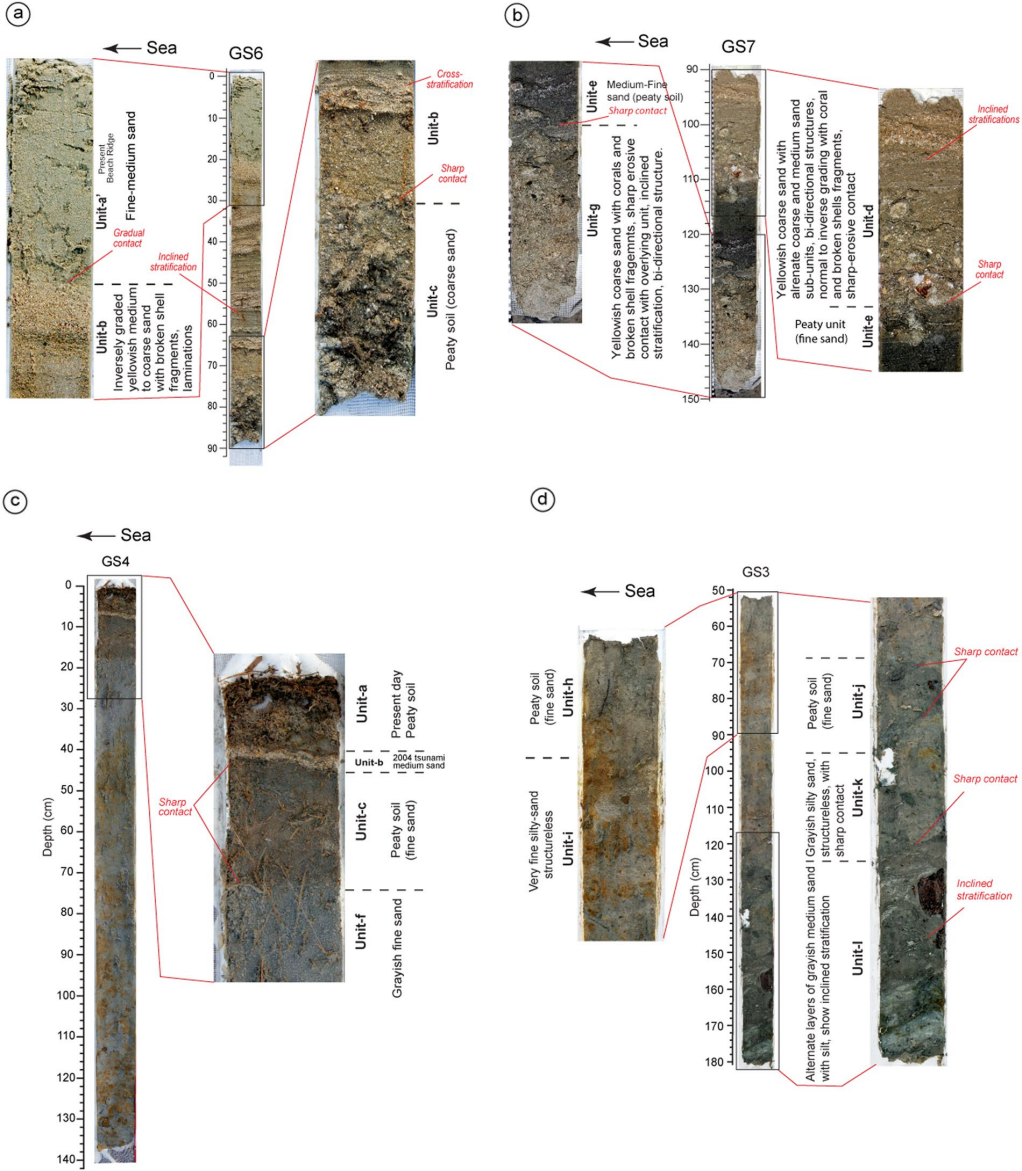
**(a)** Close-up view of 2004 tsunami deposit (Unit-b) and underlying peaty soil (Unit-c) exposed in GS6. Unit-b is marked by inversely graded yellowish medium-coarse sand with broken shells. It also shows prominent laminations, and a sharp contact with underlying unit (Unit-c). **(b)** Close-up view of Units d, e and g exposed in GS7. Unit-d (paleo-tsunami) comprises coarse to medium sand with fine gravel clasts and broken shell fragments. It shows bi-directional structure with normal to inverse grading and a sharp contact with underlying peaty unit (Unit-e). Unit-g comprises coarse to medium sand with broken shell and coral fragments. It shows inverse grading and bi-directional structure. **(c)** In GS4, the upper portion of the stratigraphy shows a thin layer of medium sand representing 2004 tsunami sandwiched between pre- (Unit-c) and post- (Unit-a) 2004 event peaty soils. Unit-b shows sharp contacts with underlying (Unit-c) and overlying (Unit-a) units. Unit-c also marks a sharp contact with underlying fine sand Unit-f. **(d)** Close-up view of geoslice GS3 collected from the base of T1-trench. The stratigraphy in the upper portion shows Unit-i with a gradual contact with overlying peat (Unit-h). Lower portion exhibits well-preserved paleo-tsunami deposit (Unit-l) with a sharp contact with Unit-k. Unit-l is ~30 cm thick medium sand-silt comprised of broken shells, coral fragments and rip-up plant material. It shows prominent inclined stratification. Unit-k comprised of silty-sand also shows sharp contact with overlying peat (Unit-j). Refer Fig. 2a,b for location.

**Figure 4:**
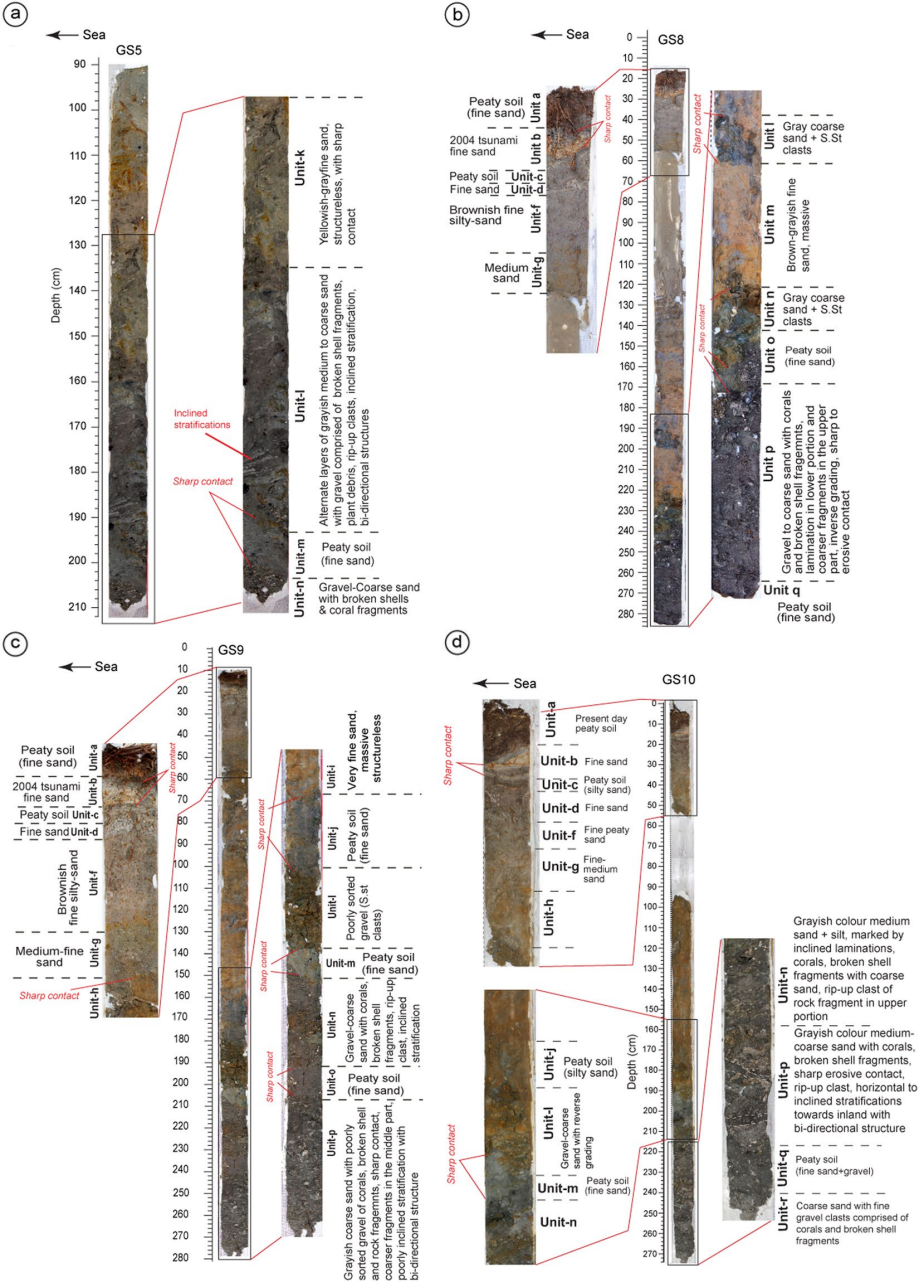
**(a)** The GS5 geoslice was collected from further deeper portion of the trench T1. About 40–45 cm thick paleo-tsunami deposit (Unit-l) comprises of alternate layers of medium to coarse sand with broken shell fragments, plant debris, rip-up clasts, and shows bi-directional structure. It shows sharp contacts with overlying Unit-k and underlying Unit-m. **(b)** GS8 displays a thin layer of 2004 tsunami (Unit-b), shows a sharp contact with overlying and underlying peaty units. This inland section also shows a very thin lens of Unit-d (fine sand) and Unit-g (medium-fine sand). Unit-l is greyish coarse sand with gravel (sandstone) clasts, shows sharp contact with Unit-m. Unit-P is thickest, comprised of gravel to coarse sand along with broken shells and coral fragments. **(c)** Geoslice GS9 exhibits Units-b, d, g, l, n and p – indicative of tsunami deposits. Unit-b shows sharp contacts with underlying and overlying units. Units d and g comprised of medium-fine sand are paleo-tsunami deposits, mark sharp contacts with underlying units. Units l, n and p represent paleo-tsunami deposits, separated by peaty units m and o. Unit p consist of coarse sand and gravel clasts, broken coral and shell fragments, and shows sharp contact with the overlying Unit-o. Unit-n made-up of coarse sand with inclined stratification shows sharp contact with the overlying and underlying units (Units m and o). **(d)** The GS10 geoslice collected from back-marsh shows thin medium sand layer of 2004 tsunami sandwiched between peaty soils pre- (Unit-a) and post- (Unit-c) 2004 event with sharp contacts. Unit-l comprised of sandstone clasts (gravel) and coarse sand shows a gradual contact with the overlying Unit-j and a sharp contact with underlying Unit-m. Units n and p are paleo-tsunamis sharing the same contact. Unit-o is missing in this section. Unit-n is a medium sand unit showing inclined laminations, rip-up clasts and broken shell and coral fragments. Unit-p is medium-coarse sand with coarser fragments of coral clasts and broken shells with bi-directional structures. Unit-p shows sharp contacts with underlying Unit-q (peaty soil) and overlying paleo-tsunami (Unit-n). Unit-r comprise coarse sand with fine gravel clasts, occurs at a depth of ~270 cm. Refer Fig. 2a,b for location.

Based on the sedimentary structure, grain size, depositional and/or erosional contacts observed in litho-sections, 19 litho-units (a’, a-r, from top to bottom) were identified (Figs. 2b, 3 and 4; Supplementary Data S2; Figs. S2.1–S2.4, Tables S2 and S5.1). **Unit-a’** is the youngest lithounit, represent present day beach-ridge facies. **Unit-a** is present-day peaty soil (humic), medium-fine sand observed from the back-marsh. **Unit-b** is 2004 tsunami yellowish medium-coarse sand. It is coarser and thicker near to the coast, and becomes thinner and finer towards inland (Fig. 1c, 2b, 3a,c and 4c,d; Supplementary Figs. S2.1–S2.4). It shows sharp contact with **Unit-c**, a peaty soil in the back-marsh that existed during 2004 event (Figs. 2b, 3a,c and 4b–d; Supplementary Figs. S2.1–S2.4). **Unit-d** is yellowish medium-coarse sand, with few broken shells and coral clasts. It shows a sharp contact with Unit-e. It seems that similar condition like 2004 existed at the time of deposition of Unit-d by historic tsunami over **Unit-e**, representing a wetland soil (Figs. 2b, 3b and 4b–d; Supplementary Fig. S2.3a,b). **Unit-f** is fine silty-sand, represents the phase of basin-filling (Figs. 2b, 3c and 4b–d; Supplementary Figs. S2.1–S2.4). **Unit-g**, comprising of medium-coarse sand, broken shell fragments, and angular coral clasts, shows bi-directional structure and sharp contact with **Unit-h** (partially developed peat) suggests that the Unit-g was associated with a tsunami (Figs. 2b, 3d and 4c,d, Supplementary Figs. S2.2–S2.4). This event caused subsidence as marked by the overlying finer Unit-f.

**Unit-i** is structureless grayish fine silty-sand, with sharp to gradual contact with Unit-j. Considering the thickness of 55–110 cm and finer nature of the unit, we infer that deposition took place during basin-filling, and the area remained submerged for a longer span during inter-seismic period (Figs. 2b and 4c; Supplementary Figs. S2.3–S2.4). **Unit-j** is peaty soil with fine sand, shows gradual contact with the overlying unit and a sharp contact with the Unit-k. We infer that the area was at or above mean sea-level before subsidence. (Figs. 2b, 3d and 4c,d; Supplementary Figs. S2.3–S2.4). **Unit-k** is yellowish medium-fine sand with scattered fine gravels, deposited in intertidal to subtidal condition (Fig. 2b; Supplementary Figs. S2.2–S2.4). **Unit-l** is greyish brown medium-coarse sand with coral clast and broken shells, show sharp contacts with Units m and k (Figs. 2b, 3d and 4a–d; Supplementary Figs. S2.3–S2.4). The unit is thicker and coarser towards the ocean, and finer and thinner towards inland. It shows prominent inclined stratification with bi-directional structure, alternative layers of greyish medium-coarse sand with silt and fine gravel clasts containing broken shell and coral fragments, plant debris, and rip-up clasts of bedrock (Fig. 2b, 3d and 4a–d). We suggests that this unit was deposited by a tsunami triggered by an earthquake that caused coseismic subsidence as indicated by overlying finer Unit-k (Fig. 2b). **Unit-m** is peaty soil with silty-sand, which separates Unit-l from Unit-n with sharp contacts (Figs. 2b and 4a–d; Supplementary Figs. S2.2–S2.4). Considering the study area in proximity to the ocean, we infer that this peaty unit was formed due to land-level change (Fig. 2b). **Unit-n** is greyish medium-coarse sand, marked by inclined laminations, with thin layers of coarser fragments comprised of broken shells, and rip-up clasts in the upper portion. It shows sharp contact with underlying peat Unit-o, which formed at or above mean sea-level (Figs. 2b and 4b–d; Supplementary Figs. S2.3). We infer that this unit (Unit-n) was deposited by tsunami generated by local event along the Andaman segment (Figs. 2b and 4b–d; Supplementary Figs. S2.3). **Unit-p**, a thick grayish coarse sand, with corals clasts, broken shells and rock fragments, is exposed at a depth of ~2 m, and shows sharp contacts with Unit-o and Unit-q (Figs. 2b and 4c,d). This unit in some sections shows poor lamination, with scattered gravels observed in the upper, and the middle portions as well as inverse grading (Fig. 4b–d). The unit was deposited by a tsunami event (Fig. 2b). **Unit-q** is a peaty unit composed of greyish fine sand with scattered gravel fragments (Figs. 2b and 4b). It shows sharp contacts with the underlying and overlying units (Fig. 2b), and possibly formed at or above mean sea level. **Unit-r** is coarse sand with broken shell fragments, deposited by a tsunami event (Figs. 2b and 4d).

**Figure 5:**
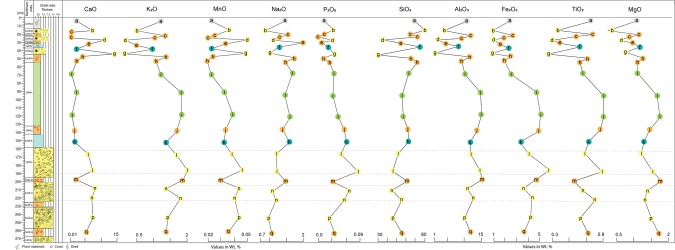
Variabilities in major oxides abundances (wt. %) in the complete litho-stratigraphic section (from Unit-a to Unit-q). The tsunami Units (marked by yellow color, Units b, d, g, l, n, p) show distinct enrichment in CaO, MnO, and P_2_O_5_, and depletion in K_2_O, and Na_2_O compared to those in the adjacent non-tsunami (terrigenous) Units. Other oxides like SiO_2_, Al_2_O_3_, Fe_2_O_3_, TiO_2_, and MgO show a clear depletion in the younger tsunami deposits, i.e., Units b, d, and g (in yellow), except in Unit-b with higher SiO_2_. All elements show a comparatively higher concentration in the older tsunami deposits (Units l, n, and p). Whereas, all the adjacent non-tsunami deposits, i.e. Units a, c, e, f, h, i, j, k, m, and q show medium to high concentration of the elements like Si, Al, Fe, Ti, and Mg, and thus, are indicative of terrigenous deposits. Also, the overall depletion of these elements within Units b, d, g, l, n, and p characterize tsunamigenic origin of these deposits. The Ca and Mn enrichment is a characteristic signature indicating marine origin of these sediments. Though Na and K ions constitute a significant part of the seawater, their depletion in the tsunami deposits is suggestive of chemical alteration through ion-exchange due to leaching and prolonged burial.

**Figure 6:**
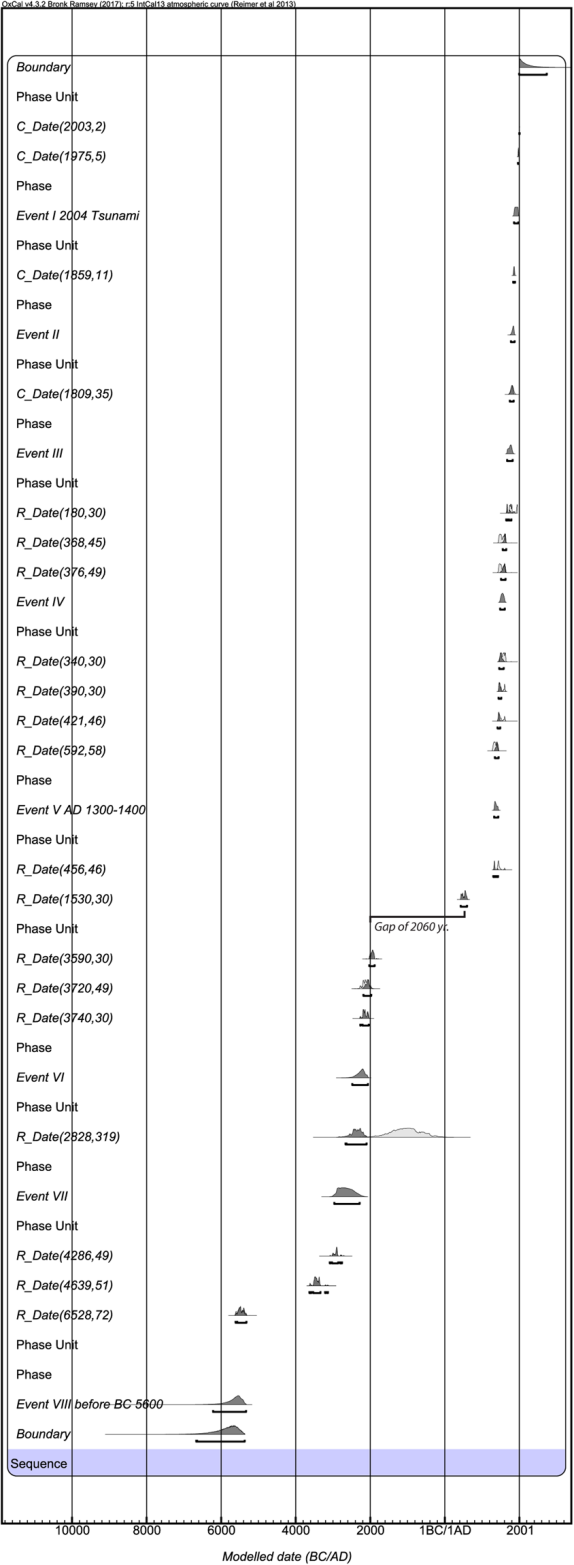
Modelled calendar ages and probability distributions of stratigraphy exposed in geoslices and trenches excavated along WNW-ESE transect at Badabalu. The ages are calculated and modeled using OxCal version 4.2.4 (Reimer *et al*.^22^) and IntCal13 atmospheric curves (Reimer *et al*.^23^). We infer at least eight events (earthquakes/tsunamis) those occurred in last 8000 yrs. Event I represents 2004 tsunami; Event II was around AD 1777–1883, could be correlated with AD 1881 earthquake and tsunami from Car Nicobar. Event III occurred during AD 1674–1821, could be correlated with AD 1762 earthquake/tsunami reported from Arakan Suduction Zone. Event IV was around AD 1485–1610, correlated with AD 1672 reported from Andaman Island. Event V was around AD 1325–1434, correlated with AD 1300–1400 earthquake and tsunami from Andaman, Thailand and Indonesia. Event VI was around BC 2480–2060, could be correlated with a tsunami reported from southeast Sri Lanka that occurred during BC 2000–3000. Event VII occurred during BC 2966–2286, correlated with tsunami event that occurred during BCE 2810-3200 reported from southeast Sri Lanka, and also with the event of BCE 2892-1895 reported from Indonesia. Event VIII occurred before BC 5600, correlated with event that occurred during BCE 5786-5301 and tsunami during BCE 5374-5579 reported from Indonesia. Conventional radiocarbon ages, OSL ages and other relevant details are summarized in Tables S4.1 and S4.2. Areas with white outlines indicate the probability distributions functions (PDFs) of calibrated radiocarbon ages. Gray areas represent posterior PDFs. Brackets below each PDF are 2σ uncertainties.

Synthesis of sedimentological (structures, grain size, lithology), geochemical (major and trace element abundances) and biological (foraminifera) data suggest that Units b, d, g, l, n, p and r were deposited by sudden high-energy wave events – tsunamis^3,18,19^ (Figs. 2–6; Supplementary Data S2–S5). Clear discrimination between tsunami and storm deposits is difficult. However, most of the cyclones around Anadman start to develop at their initial stage and are not strong enough to affect the sedimentation pattern^3^. This rules-out the possibility of the identified deposits to be non-tsunami origin. Also, the lithounits identified in the exposed geoslicers and trenches show distinct sedimentological signatures like alternate layers of medium sand and silt or coarse sand, broken shell and coral fragments, poorly sorted sediments, normal to inverse grading, rip-up clasts, plant material, inclined stratification, bi-directional structures etc. Further, it is also argued that usually tsunami deposits show layers with bi-directional flow, i.e., towards landward and seaward directions, whereas, storm or cyclone deposits do not show such bi-directional flow^20,21^. Tsunami deposits usually show bi-modal distribution of grain size, whereas, storm deposits are well sorted^21^. In our study we found layers with bi-directional flow as well as bi-modal distribution of grain size. Hence, we conclude that the deposits from Badabalu are deposited by tsunami events.

### Micro-fossil analysis

Quantitative analysis of foraminifera obtained from Units b, d, e, g, n and p indicated two distinct biofacies suggestive of marginal marine environments and sediment provenance: Biofacies-I: subtidal, and Biofacies-II: intertidal (Supplementary Data S3; Figs. S3.1–S3.3; Tables S3.1–S3.2). Units p and n exposed from the 350 m inland section show two sediment sources derived from marginal marine environment. Unit-n corresponds to the Biofacies-I, comprises majority of subtidal species like *Rotalia* sp., and *Eiphidium crispum* with minor amount of *Quinqueloculina seminulam*, and *Amphistegina Amphistegina lobifera*. Whereas, Unit-p shows dominance of *Ammonia beccarii* - an intertidal species (Supplementary Fig. S3.3). Foraminifera assemblages from Units b, d, and g also show same kind of species and taphonomy. These units also show high percentage of abraded and fragmented foraminifera test (Supplementary Fig. S3.1). The peaty soil Unit-e with *Elphidium discoidale* suggests shallow intertidal-beach environment. The change in the environment from intertidal-beach to wetland is attributed to interseismic uplift. This further strengthen our interpretation that Units b, d, g, n and p were deposited by tsunami events, which transported and deposited forams from different depths (Supplementary Data S3).

### Dating (OSL and AMS)

**Figure 7:**
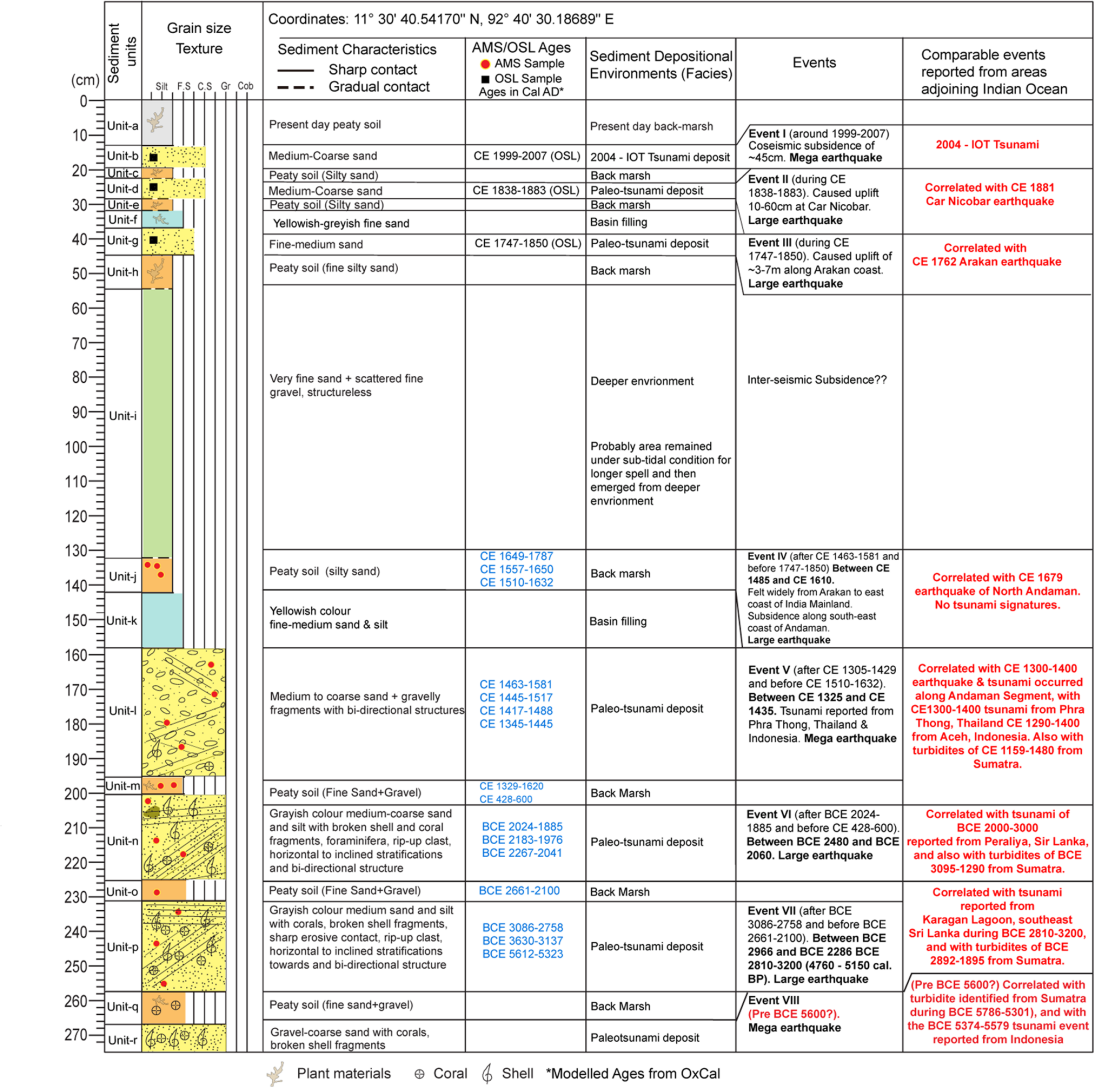
Composite stratigraphic-section constructed using the litho-sections obtained from geoslices and trenches at Badabalu, south coast of Andaman Island.

From the exposed succession (geoslices + trenches) at Badabalu, we obtained 22 ages (Figs. 2b, 6 and 7; Tables 1 and 2; Supplementary Data S4; Tables S4.1 and S4.2; Fig. S4.1). Eighteen radiocarbon (Accelerator Mass Spectrometer, AMS) ages were obtained by dating charcoal, buried wood, and plant material, along with four Optically Stimulated Luminescence (OSL) ages of the sediment samples. The ages range from cal BCE 5600 to cal CE 2000. All ages were calibrated and modelled with OxCal v.4.2.4 to obtain the calendar ages and events (Fig. 6)^22,23^. A distinct depositional gap of 2000 years was observed between 3590 and 1530 cal BCE (Fig. 6).

**Table 1:** ^14^C Accelerator Mass Spectrometry (AMS) ages from Badabalu (for more details refer Supplementary Table S5.1).

Sr. No.	Sample site Geoslice/Trench no.	Sample no.	Sedimentary Unit	Depth in cm	Material	^14^C age yrs BP	Calendar age (2σ) from OxCal	Modelled Age from OxCal
1	TS2	TS2Ca	c	15	Charcoal	102.4 + /− 0.3 pMC	Modern	
2	GS9	JNMGS9C1	h	44	Buried wood	3235 ± 547	**BCE 3033–348**	—
3	GS3	JNMGS3a	j	125	Coconut clast	180 ± 30	**CE 1652–1917**	**CE 1649–1787**
4	GS3	JNMGS3b	j	130	Root	376 ± 49	**CE 1442–1636**	**CE 1510–1632**
5	GS9	JNMGS9C2	j	148	Charcoal	368 ± 45	**CE 1446–1636**	**CE 1557–1650**
6	GS3	JNMGS3c	l	154	Buried wood	340 ± 30	**CE 1470–1640**	**CE 1463–1581**
7	GS3	JNMGS3d	l	163	Buried wood	592 ± 58	**CE 1286–1426**	**CE 1345–1445**
8	GS5	JNMGS5C3	l	174	Buried wood	390 ± 30	**CE 1441–1631**	**CE 1445–1517**
9	GS5	JNMGS5C2	l	182	Charcoal	421 ± 46	**CE 1416–1632**	**AD 1417–1488**
10	GS5	JNMGS5C1	m	196	Buried wood	1530 ± 30	**CE 428–600**	**AD 428–598**
11	GS9	JNMGS9C3	m	190	Charcoal	456 ± 46	**CE 1329–1620**	**AD 1305–1429**
12	GS5	JNMGS5C5	n	204	Buried wood	3590 ± 30	**BCE 2028–1885**	**BCE 2024–1885**
13	GS5	JNMFORBB1	n	208	Foraminifera	3740 ± 30	**BCE 2275–2035**	**BCE 2267–2041**
14	GS10	JNMGS10C1	n	216	Charcoal	3720 ± 49	**BCE 2284–1972**	**BCE 2183–1976**
15	GS8	JNMGS8C2	o	222	Charcoal	2828 ± 319	**BCE 1877–211**	**BCE 2661–2100**
16	GS8	JNMGS8C1	p	260	Charcoal	4286 ± 49	**BCE 3082–2704**	**BCE 3086–2758**
17	GS9	JNMGS9C4	p	228	Charcoal	4639 ± 51	**BCE 3630–3137**	**BCE 3630–3137**
18	GS9	JNMGS9C5	p	272	Buried wood	6528 ± 72	**BCE 5621–5359**	**BCE 5612–5323**

Samples processing and measurements were carried out at Beta Analytics, USA and IUAC, New Delhi.

“Calibrated” or calendar ages were calculated using “CALIB rev 5.01” and calibration curves (IntCal04, Reimer *et al*.^22,23^).

**Table 2:** Optical Stimulation Luminescence (OSL) ages from Badabalu (for more details refer Supplementary Table S5.2).

Sr. No.	Sample site Geoslice/Trench no.	Sample no.	Depth in cm	Sedimentary Unit	Material	^a^Age in BP	^b^Age in CE/BCE Cal. from OxCal	Modelled Cal. Age from OxCal
1	TS1	TSTL1	17	b	Fine-sand	11 ± 2	**CE 1999–2007**	**CE 1999–2007**
2	TS1	TSTL2	35	d	Fine-sand	155 ± 11	**CE 1836–1881**	**CE 1838–1883**
3	TS3	TS3TL1	70	b	Medium-coarse sand	39 ± 5	**CE 1965–1985**	**CE 1965–1986**
4	GS10	GS10a	34	g	Fine-sand	205 ± 35	**CE 1739–1879**	**CE 1747–1850**

^a^Samples processing and measurements were carried out at Indian Institute of Technology Kanpur.

^b^“Calibrated” or calendar ages were calculated using “OxCal” program.

The OSL ages gave some offset due to over-dispersion of De (Equivalent Dose), hence the Minimum Age Model (MAM) was used (Fig. S4.1). Although, the age obtained from 2004 tsunami sand was the most reliable, the estimated age gave an offset of 20-40 years. Similar results of residual charge equivalent to <50 year have been observed in the 2004 tsunami deposits from India^24^; 60–120 years from Lisbon 1755 tsunami^25^; and 20–40 years from Thailand tsunami^26^.

### Geochemical analysis

The major oxides and trace elemental abundances in sediments from Badabalu revealed characteristic signatures similar to that observed in other global tsunami deposits (Fig. 5; Supplementary Data S5; Fig. S5.1; Table S5.1). Tsunami and non-tsunami deposits (i.e., terrigenous) identified on the basis of sedimentological and microfossil proxies showed differences in geochemical signatures (Supplementary Data S3 and S5). In the upper section (<50 cm), the tsunami Units b, d, and g have distinctly lower abundances of Al_2_O_3_, Fe_2_O_3_, and K_2_O compared to those of the intermittent (non-tsunami) Units a, c, e, f, and h (Fig. 5). However, the tsunami layers (Units l, n, and p) in the bottom section (>150 cm) do not show lower abundances of Al_2_O_3_, Fe_2_O_3_, and K_2_O relative to the adjacent non-tsunami Units k, m, and q (Fig. 5). Interestingly, the tsunami deposits in the bottom section are thicker compared to those in the upper portion. It is possible that the some of the distinct geochemical signatures in these older tsunami deposits are disturbed due to prolonged burial and leaching^27^. On the other hand, all the tsunami Units b, d, g, l, n and p contain distinctly higher CaO and MnO than the other terrigenous Units a, c, e, f, h, i, j, k, m, o, and q. High abundance of CaO and MnO is a characteristic signature in tsunami deposits^27^. In general, the terrigenous units are also characterized by the higher contents of SiO_2_, TiO_2_, MgO and Na_2_O compared to the tsunami units (Fig. 5). The major oxides abundances further strengthen our interpretation that Units b, d, g, l, n and p are of marine origin, and deposited inland during tsunami events.

Compared to the terrigenous units, the tsunami Units b, d, g, l, n and p are generally enriched in alkali elements, in particular Ca, Na, K, Sr and have higher Ca/Sr, Na/K, and Sr/Ba ratios (Supplementary Fig. S5.1; Table S5.1). Higher Rare Earth Elements (REEs) and Large Ion Lithophile Elements (LILE: Rb, Ba), and Transition Trace Elements (TTE: Cu, V, Cr, Co, Ni) abundances in tsunami units indicate sediment input from submarine sources (Supplementary Figs. S5.1). The Ce anomaly (Ce/Ce* = Ce_CN_/(La_CN_ × Nd_CN_)^0.5^; CN is chondrite-normalized) has been considered as a paleo-oceanographic indicator of widespread marine anoxia^28^. The chondrite-normalized REE patterns of the majority of the tsunami samples exhibit a distinct positive Ce anomaly with the Ce/Ce* ratio varying from 1.0 to 2.55 (Supplementary Data Table S5.1). Cerium can exist in +3 or +4 oxidation states depending on the redox conditions. The insoluble Ce^4+^ is prone to be adsorbed and sequestered by Mn-oxides and hydroxides under oxidizing environment and thus marine sediments rich in Fe–Mn exhibit positive Ce anomalies^29^. The range of Ce/Ce* values (1.0 to 2.55) in the sediments from tsunami layers confirm an origin in anoxic to suboxic environment. Thus, characteristic geochemical signatures in Units b, d, g, l, n and p further affirms their tsunamigenic origin.

## Discussion and Conclusions

The signatures of 2004 Sumatra-Andaman earthquake and tsunami were considered as a modern analogue to distinguish the role of local and distant source earthquakes towards the deposition of tsunami deposits. At Badabalu, we found relatively thicker and coarser deposits (Units l, n and p) as compared to Unit-b deposited by 2004 tsunami. The presences of thicker deposits could be attributed to the paleo-shoreline morphology. Possibly at the time of deposition the beach-ridge and associated back-marsh were located farther inland relative to the present coastline configuration, with deposition taking place in a swale or back-marsh area. Further, the coarser and thicker deposits could be related to tsunami events with much higher energy conditions, which was possible by a major earthquake triggered along the Andaman-Arakan Segment, suggesting a local earthquake. This is well justified comparing the 2004 tsunami deposit at the same location. Therefore, we infer that the thicker and coarser units (viz. Units l, n and p) were deposited by the local-source earthquakes those occurred along the Andaman-Arakan Segment. Paleo-tsunami and paleoseismic events identified in the present study were correlated with the reported events from the areas adjoining Indian Ocean (Supplementary Table S1).

The present study from Badabalu revealed evidence of at least seven tsunami events in the last 8000 years (Figs. 2b, 6 and 7; Supplementary Data S4a-b; Tables S4.1 and 4.2). These events were bracketed based on their modelled calendar ages^22,23^ (Tables 1 and 2; Supplementary Tables S4.1 and S4.2). The **Event-I** (Unit-b), having an OSL age of CE 1999–2007, represents the 2004 Sumatra-Andaman tsunami (Table 2; Supplementary Tables S1 and S4.2). **Event II** (Unit-d) occurred around CE 1838–1883, after CE 1747–1850 and before CE 1999–2007 (Figs. 2b, 6 and 7; Tables 1 and 2; Supplementary Table S1). We correlate this event with the 1881 (Mw 7.9) Car Nicobar earthquake, which was felt over much of India and parts of Burma as well as in the Bay of Bengal^30^. This was a local event that occurred along the Andaman segment, generated 0.8 m high tsunami, resulted in an uplift of 10–60 cm at Car Nicobar^30^, but did not have a widespread effect. **Event-III** (Unit-g) took place around CE 1747–1850, after CE 1649–1787 and before CE 1838–1833, and correlates with an earthquake of CE 1762 (Mw7.5) (Figs. 2b, 6 and 7; Tables 1 and 2; Supplementary Table S1). This event occurred along the Arakan Subduction Zone that caused uplift of ~3–7 m along the coasts of Ramree, Cheduba, and Foul Islands, located offshore of the Arakan coast of Myanmar^10^, and also generated a tsunami^31^. Signatures of liquefaction and tsunami deposit were also reported from Mitha-Khadi around Port Blair in Andaman Island^6^. However, no clear evidence of land-level change was found at the present study site. This suggest that Andaman was at the southern tip of this rupture. **Event-IV** (Unit-j) occurred after CE 1463–1581 and before CE 1747–1850. The event correlates with the historic earthquake of CE 1679 (Figs. 2b, 6 and 7), which was felt around Arakan (Burma), Bangladesh, Chennai, and areas adjoining Indian Ocean^32^. This event may also be correlated with: (i) the event occurred along the Andaman Segment, that accompanied land-subsidence during CE 1600 from Mitha-Khadi near Port Blair^6^, (ii) a tsunami event of CE 1640–1950 reported from Sumatra^8^, and/or (iii) with a tsunami event of CE 1530–1730 (380 ± 50 cal. BP) from Thailand^31^. Considering its wide-spread effect we suggest that this event was triggered along the Andaman Segment and was comparatively larger than CE 1881 (Event II), inflicting wider effect in the Indian Ocean. **Event V** (Unit-l) occurred after CE 1305–1420 and before CE 1510–1632 (Figs. 2b, 6 and 7), which correlates with the CE 1300–1400 tsunami reported from Phra Thong, Thailand^12,13,33^ and CE 1290–1400 tsunami from Aceh, Indonesia^8^. This event may also be correlated with the CE 1120–1300 tsunami event reported from the west coast of Andaman^3^, and with turbidites found from Sumatra (T3: 630 ± 110 cal. BP; CE 1159–1480)^14^. Also, signatures of subsidence and tsunami deposit during CE 1040–1495 have been reported from the south Andaman, as well as an uplift from Hut Bay and north Andaman^11,12^. This was a mega earthquake sourced locally along the Andaman segment and resulted in a transoceanic tsunami^3^. **Event VI** (Unit-n) occurred after BCE 2660–2100 and before CE 428–600 (Figs. 2b, 6 and 7, Tables 1 and 2; Supplementary Table S1). Further considering the youngest age of detrital charcoal from Unit-n (BCE 2024–1885), we suggest that Event VI occurred after BCE 2024–1885. This event may be correlated with turbidites observed in a core (T26: 3720 ± 340; BCE 3095–1290) from Sumatra^14^, and also with the tsunami of BCE 2000–3000 reported from Peraliya, Sir Lanka^34,35^. We infer that Event VI was a local event produced by Andaman Segment, generated a tsunami that reached the eastern coast of India and Sri Lanka. **Event VII** (Unit-p) was after BCE 3086–2758 and before BCE 2661–2100, during BCE 2966–2286 (Figs. 2b, 6 and 7, Tables 1 and 2). Due to a wider age bracket it is difficult to correlate this event with a particular event reported from other adjoining areas in the Indian Ocean. Nevertheless, it may be correlated with the BCE 2810–3200 (4760–5150 cal. BP) tsunami reported from Karagan Lagoon, southeast Sri Lanka^34,35^, and also with turbidites reported from Sumatra (T27: 3900 ± 190 cal. BP; BCE 2892–1895)^14^. Since we do not have any lower limit bracketing **Event VIII**, we suggest that **Event VIII** (Unit-r) occurred before BCE 5612–5323 (Figs. 2b, 6 and 7, Tables 1 and 2; Supplementary Table S1). This event can be correlated with turbidite identified from Sumatra during (T43: 6600 ± 140 cal. BP; BCE 5786–5301)^14^, and with the BCE 5374–5579 (7324–7529 cal. BP) tsunami event reported from Indonesia^36^. Because we found this tsunami deposit in only one geoslice (Fig. 2b), it is difficult to ascertain if this was a local (Andaman segment) or a distant sourced event.

Based on the stratigraphic record, OSL and ^14^C AMS ages, and modelled ages in OxCal, we observed a considerable depositional gap for almost 2000 year between 3700 and 1500 years BP (Figs. 2b, 6 and 7, Supplementary Data S4, Table S4.1–S4.2). This discontinuous stratigraphic record could be attributed to erosion due to one of the possibilities: (a) coseismic uplift or gradual uplift during inter-seismic period along the up-dip portion of the subducting plate or upper plate fault. But no upper plate fault from this region has been reported; (b) Relative Sea Level (RSL) fall which accelerated erosion of the stratigraphic sequence. The chronology of the beach ridges and reconstruction of complex pattern of shoreline progradation and erosion from Phra Thong, Thailand suggest a short episode of local erosion between 4000 and 3800 yr BP, could be attributed to climate change, impact of a tsunami or tropical cyclone^37^. Further, Brill *et al*.^37^ also reported a signature of sea-level fall and shoreline progradation from Phra Thong with decreased rate of <1 m/year during 3300–3500 yr BP. Dura *et al*.^38^ reported an incomplete record of subduction zone earthquakes in coastal stratigraphy from the coast of Sumatra, because the late Holocene (last 4 ka) RSL or sea-level fall facilitated erosion and restricting preservation of lithounits. Following these arguments, we suggest that erosion was accelerated due to RSL fall at ~3500 BP in Andaman and Nicobar Islands. The continuous stratigraphic sequence from 1500 years BP till present is attributed to a gradual RSL rise, which remained within the tidal frame of 1–2 m in the Andaman region.

The lower portion of the stratigraphic section comprising Units l to r reveals stacked sequence of peaty units (wetland soils; Units m, o and q) and tsunami deposits (Figs. 6 and 7). The Unit-k comprising fine-medium silty-sand suggests basin-fill under sub-tidal condition followed by a gradual uplift during inter-seismic period. The peaty soil (Unit-j) indicates the formation of wetland soil at or above mean sea-level. This was followed by a coseismic subsidence, and a long-term post-seismic subsidence is well justified by the presence of a 75 cm thick very fine sand (Unit-i). The presence of Unit-h (peaty soil) suggests that the area was at or above mean sea-level. We infer that the area emerged from deeper environment (sub-tidal) around this time.

The upper portion of the stratigraphic section, Unit-b marks the 2004 tsunami, Units d and g represents tsunamis during the recent historic time (Figs. 5–7). Unit-f with fine sand suggests that the area was under the influence of sub-tidal environment, whereas Units e and c (peaty soils) indicate that the area was at or above mean sea-level. Possibly the area experienced subsidence during these earthquakes and recovered during post-seismic period, which eventually facilitated the formation of wetland soils and vegetation growth. The area remained submerged for substantially longer span during inter-seismic period as indicated by a thick fine silty-sand (Unit-i) (Figs. 6 and 7). A long-term inter-seismic subsidence implies a huge strain accumulation. However, couple of large magnitude earthquakes viz. CE 1881, with a rupture near Car Nicobar in the mid-segment of Andaman, and CE 1762, ruptured along the Arakan Subduction Zone, partially released the long-term accumulated strain after CE 1679 event. The CE 1679 event was a local event having its rupture along the Andaman Island. Hence, we conclude that the Andaman Segment has enough accumulated strain to trigger a mega- tsunamigenic subduction zone earthquake in near future. A 2000 years stratigraphic gap add to the uncertainty associated with the estimation of the recurrence of tsunamigenic earthquakes. However, 1500 years of continuous sequence suggests a recurrence of 420–750 years for a mega-earthquakes along subduction zone like the 660–880 CE^3^, 1300–1400 CE and the 2004 Sumatra Andaman earthquake having different source. A shorter interval of 80–120 years is inferred for the large earthquakes like 1679, 1762 and 1881 CE.

## Methods

Google Earth images (pre and post 2004 earthquake) were used to identify the location that experienced land-level change and having a shoreline configuration with beach-ridge-swale topography, which are ideal for the preservation of tsunami deposits (Supplementary Figs. S1.1a–h and S1.2a,b). A detailed topographic survey using Total Station was conducted transverse to the shoreline along the WNW-ESE transect (Figs. 1d and 2a).

We identified typical signatures of paleo-earthquakes and paleo-tsunamis from shallow stratigraphy at Badabalu (Figs. 1c,d and 2a,b; Supplementary Figs. S2.1–S2.4). The area is marked by typical beach ridge-swale-beach ridge topography, with a distinct back-marsh (Figs. 1d and 2a,b). Such geomorphic setting is considered to be an ideal sites for the preservation of tsunami deposits^13^. Three 1–1.5 m deep trenches (T1-T3; Fig. 2a) were excavated, and 10 geoslices (GS1-GS10) 1.5–3 m deep along a WNW-ESE transect normal to the coastline were obtained (Figs. 1d and 2a,b; Supplementary Data S2, Figs. S2.1–S2.4). All exposed stratigraphic sections studied are perpendicular to the shoreline. Lithounits in the exposed sections were classified based on their sedimentological characteristics in the field (e.g., color, grain size, contacts etc.) (Figs. 2b, 3a–d and 4a–d; Supplementary Figs. S2.1a–e and S2.2–S2.4). To further strengthen our interpretations towards differentiating tsunami and non-tsunami deposits, we preformed geochemical and micro-fossil analysis (Fig. 5; Supplementary Data S3 and S5; Figs. S3.1–3.3; S5.1). The foraminifera analysis was carried with a standard methodology (Supplementary Data S3; Fig. S3.1 and Table S3.1). To identify the environment foraminifera we carried out Q-mode cluster analysis using Constrained Incremental Sum of Squares (CONISS) method^39^, Fisher Alpha Diversity Index (FADI)^40^, Detrended Correspondence Analysis (DCA), and Hierarchical Dendrograms^41^ (Fig. S3.2).

Four sediment samples were dated by Optical Stimulated Luminescence (OSL) dating technique at IIT Kanpur, and 18 samples were dated for ^14^C (AMS) ages at Beta Analytic, USA, as well as at Inter University Accelerator Centre (IUAC), New Delhi (Supplementary Data S4; Figs. S4.1–S4.3). We collected sediment samples from the exposed trenches as well as geoslices obtained from Badabalu site (Figs. S2.1–S2.11; Table S4.2). For paleodose measurement, samples were treated with 1 N HCl for one hour followed by washing the sample at least three times with de-ionized water. It was followed by treatment with 30% H_2_O_2_ until all the effervescence disappeared, and washed again with de-ionized water. This is done to get rid of carbonates and organic matter from the sediments. Dried samples were then sieved to obtain 90–212 um grain fractions of which only 90–125 um fraction size was used for further analysis. The quartz and feldspar were isolated with the help of Frantz magnetic separator with constant current of 1.50A. Then the isolated quartz was etched with 40% Hydrofluoric acid (HF) solution for 60 minutes to remove outer alpha skin and dissolve any leftover feldspar. The isolated quartz was then rinsed with HCL to get rid of any fluorite precipitate from HF acid. After drying, the sample was re-sieved to remove <90 um to acquire fine pure quartz grains. These grains were then mounted on 9.8 mm diameter stainless steel aliquots with the help of silicon spray. All the processing was carried out in the laboratory controlled red light environment. For the paleodose determination, Riso TL/OSL reader with an EMI 9635Q photomultiplier and two 3 mm Hoya U-340 filters were used. Ages were calibrated and modelled with Bayesian analysis in the program OxCal v.4.2.4 to get calendar ages and events^22,23^ (Fig. S4.2; Supplementary Table S4.1 and Fig. S4.2).

To examine the geochemical signatures of the near-surface coastal stratigraphy from Badabalu site, we analysed 16 samples from 17 litho-units (Units a to q, except from Unit-o) for major oxides and selected trace element abundances (Fig. 5 and Supplementary Data S5; S5.1). Major oxides abundances were determined using a wavelength dispersive X-ray Fluorescence Spectrometry (WD-XRF, Rigaku ZSX Primus II) and trace element concentrations determined using an Inductively Coupled Plasma Mass Spectrometer (ICP-MS, Thermo Fisher Scientific iCAPQ) at IIT Kanpur. Detailed analytical procedure is given by Chandra *et al*.^42^. XRF analyses were carried out on fusion glass beads and ICP-MS analyses were done on samples digested using HF-HNO_3_ mixture. Based on three repetitive measurements of geo-standards (LKD-2, SBC-1, WGB-1, AGV-2) analysed with unknown samples, the uncertainty associated with the major elements is <5% and that for the trace elements is within the range of 3–10%.

Considering all above-mentioned data and ages (OSL and ^14^C AMS) a composite stratigraphy was generated for final interpretations towards identifying paleoseismic and associated tsunami events. Ages were used to bracket the events. An attempt was made to correlate the identified events with the events reported from adjoining areas along the Indian Ocean like Indonesia, Thailand, and Sri Lanka.

## Supplementary information

Supplementary Data
