# Supplementary material for: Tsunami records of the last 8000 years in the Andaman Island, India, from mega and large earthquakes: Insights on recurrence interval
Source: Sci Rep. 2019 Dec 5;9:18463. doi: 10.1038/s41598-019-54750-6 (PMC6895190; doi:10.1038/s41598-019-54750-6)
Supplement: Supplementary file 1 — Supplementary Data [file 41598_2019_54750_MOESM1_ESM.docx]

*Supplementary Data*

*for*

**Tsunami records of the last 8000 years in the Andaman Island, India, from mega and large earthquakes: Insights on recurrence interval**

**Javed N Malik*^1^, Frango C Johnson^1^, Afzal Khan^1, 2^, Santiswarup Sahoo^3^, Roohi Irshad^1^, Debajyoti Paul^1^, Shreya Arora^1^, Pankaj Kumar Baghel^4^, Sundeep Chopra^4^**

**^1^**Department of Earth Sciences, Indian Institute of Technology Kanpur. Kanpur 208016. Ph. +91-512-2597723; +91-9956300101. Emails: [javed@iitk.ac.in](mailto:javed@iitk.ac.in) *****Corresponding author; [frangojohnson@gmail.com](mailto:frangojohnson@gmail.com); [afzal@iitk.ac.in](mailto:afzal@iitk.ac.in); [roohi@iitk.ac.in](mailto:roohi@iitk.ac.in); [roohigeology@gmail.com](mailto:roohigeology@gmail.com); [dpaul@iitk.ac.in](mailto:dpaul@iitk.ac.in); [sarora@iitk.ac.in](mailto:sarora@iitk.ac.in); [shreyaar07@gmail.com](mailto:shreyaar07@gmail.com)

^2^Department of Energy and Environment, Babasaheb Bhimrao Ambedkar University, Lucknow 226025, Uttar Pradesh, India. Email: afzal2iit@gmail.com

^3^Department of Geology, Utkal University, Vani Vihar, Bhubaneswar 751004, Odisha, India. Email: [sahoosantiswarup@gmail.com](mailto:sahoosantiswarup@gmail.com)

**^4^**Inter- University Accelerator Centre (AMS & Pelletron Group), New Delhi 110067. India. Email: pkb@iuac.res.in; [sundeepchopra@gmail.com](mailto:sundeepchopra@gmail.com)

**S1: Land-level change caused by 2004 Sumatra-Andaman (Mw 9.3) earthquake, Geomorphology, Paleo-tsunami and Paleoseismic signatures from areas adjoining Indian Ocean.**

The 2004 Sumatra-Andaman earthquake resulted into dramatic land-level change along the Andaman and Nicobar coastline. Subsidence was along the southern Andaman Island was prominent. At Badabalu subsidence of about 40-45 cm was recorded, that resulted in landward shifting of beach, beach-ridges and back-marsh (Figs. S1.1a-h; S1.2a-b). Local roads were elevated to avoid inundation during high-tide, even local residents relocated their houses on an elevated platform (Fig. S1.3a-c). Formation of dead forest due to the intrusion of saline water and regular inundation resulted due to subsidence was common along the southern coast of Andaman Island (Fig. S1.4a-e). Intense coastal erosion of the beach, beach-ridge, and uprooting of trees along the coast was also observed (Fig. S1.4b). Artificial land-fill was done near the coastal zone to protect the agricultural fields and regular inundation during high-tides (Fig. S1.4c-e).

**S2: Stratigraphic record from Badabalu**

In total three trenches (T1 to T3) and ten geoslice sections (GS1-GS10) were obtained along the WNW-ESE transect to identify the signatures of paleo-earthquakes and paleo-tsunamis preserved in the near surface coastal stratigraphy (Figs. S2.1-S2.4). Trenches were excavated on present day beach/near the forelimb of the beach-ridge as well as behind the present beach-ridge. To have deeper stratigraphy, we obtained geoslices sections along the WNW-ESE transect almost perpendicular to the present beach. Detailed sedimentological, micro-fossil and geochemical analyses were carried to differentiate the tsunami and non-tsunami deposits (Figs. 2-6; Supplementary Data S2, S3 and S4). A composite stratigraphy is given in the main text considering all geoslice and trench sections (Figs. 2-6).

*Stratigraphy at Badabalu:*

**Unit-a’** is the youngest lithounit representing present day beach-ridge facies, grayish in colour, moderately to well-sorted fine sand (mean = 2.1ϕ; sand % = 94.5%) observed in T3 and GS6 (Figs. S2.1-S2.4).

#### Table S1: Paleotsunami and Paleoseismic signatures identified from the Andaman and Nicobar Islands and the adjoining regions^1-17^.

| Sr. No. | Location | Period | Coseismic event and evidence | Reference |
| --- | --- | --- | --- | --- |
| 1 | A&N Islands | 1. AD 770-1040 2. AD 1250-1450 | a) Extensive Paleo-root zone, subsidence b) Tsunami deposits | Rajendran et al^1, 2^ |
| 2 | a) Hut Bay, South Andaman  b) North Andaman | 1. AD 1041-1635 2. AD 1030-1495 3. c) AD 1043-1417 | a) Based on the uplifted marine terrace along the Hut Bay  b) Based on the subsidence near to Port Blair and  c) Uplift – based on uplifted marine terraces, Avis Island | Rajendran et al^1^ |
| 3 | Kaveripattinam,  South-eastern coast of India | 1. AD 850-980 2. b) AD 940-1090 | a) Tsunami deposit  b) TL date of embedded pottery sherds | Rajendran et al^3^ |
| 4 | Near Port Blair, South Andaman Island  Southwest coast of Andaman Island | 1. AD 1679 or later 2. Around AD 1762 3. Predated AD 800 4. AD 660-800 5. c) AD 1124-1274 | a) Subsidence – based on shallow stratigraphy record  b) Uplift – from shallow stratigraphy record  a) Tsunamigenic event  b) Uplift – from shallow stratigraphy record  c) Tsunamigenic event | Malik et al^4^  Malik et al^5^ |
| 5 | North of Andaman from Myanmar region – Arakan Subduction Zone | 1. BC 1395-740 2. AD 805-1220 3. AD 1585-1810 (related to AD 1762 tsunamigenic earthquake) | Uplift - based on uplifted marine terrace along the coastline | Aung et al^6^ |
| 6 | Aceh, Indonesia | 1. After AD 780-990 2. After AD 1290-1400 3. c) AD 1640-1950 | Tsunami sand sheet in the stratigraphic record | Monecke et al^7^ |
| 7 | Aceh, Indonesia | 1. 2815-2916 (cal. BP) [BC 865 – 966] 2. 3270-3341 (cal. BP) [BC 1320 – 1391] 3. 3278-3346 (cal. BP) [BC 1328 – 1396] 4. 3287-3353 (cal. BP) [BC 1337 – 1403] 5. 3304-3363 (cal. BP) [BC 1354 – 1413] 6. 5357-5575 (cal. BP) [BC 3407 – 3625] 7. 5578-5866 (cal. BP) [BC 3628 – 3916] 8. 6083-6915 (cal. BP) [BC 4133 – 4965]   i) 7324-7529 (cal. BP) [BC 5374 – 5579] | 11 – sand sheet suggestive of tsunami deposits from coastal cave | Rubin et al^8^ |
| 8 | Phra Thong, Thailand | 1. Before AD 150 2. After AD 500 3. AD 1300-1450 4. BC 800 | Tsunami sand sheet in the stratigraphic record | Jankaew et al^9^ |
| 9 | Phra Thong, Thailand | 1. 380±50 Cal. BP [AD 1570±50] 2. 990±130 to 1410±190 (cal. BP) [AD 540±190 to 960±130] 3. 2100±260 (cal. BP) [BC 150±260] 4. AD 2004 | Tsunami sand sheet from stratigraphic record | Prendergast et al^10^ |
| 10 | Phra Thong, Thailand | 1. before AD 1300 2. before AD 1900 | Tsunami sand sheet- from stratigraphic record | Fujino et al^11^ |
| 11 | Simeulue Island, Indonesia | 1. around AD 956±16 2. around AD 1394±3 3. around AD 1430±3 4. around AD 1422 5. AD 1450±3 6. AD 2004 7. AD 2005 | Uplift - based on died coral | Meltzer et al^12, 13^ |
| 12 | Mentawai Island,  Indonesia | 1. AD 1347±18 2. AD 1596±13 3. AD 1648±26 4. AD 1797 5. AD 1833 | Uplifted Coral: Uplift events with three super-cycles of the Mentawai segment, during 1300, 1600, 1797 and 1833 | Sieh et al^14^ |
| 13 | Karagan Lagoon in southeastern Sri Lanka | 1. 2004 tsunami 2. 2417±152 to 2925±98 cal. BP   [BC 467±152 to 975±98]   1. 4064±128 to 4331±126 cal. BP   [BC 2114±128 to 2381±126]   1. 4331±126 to 4583±196 cal. BP   [ BC 2381±126 to 2633±196]   1. 4764±140 to 5152±178 cal. BP   [BC 2814±140 to 3202±178]   1. 6197±156 to 6249±68 cal. BP   [BC 4247±156 to 4299±68]   1. 6249±68 to 6455±118 cal. BP   [BC 4299±68 to 4505±118]   1. 6455±118 to 6665±110 cal. BP   [BC 4505±118 to 4715±110] | a) Tsunami event from stratigraphic record  b) to g) six tsunami events occurred between 4064±128 cal. BP and 6665±110 cal. BP with a mean recurrence interval of 434± 40 yr | Jackson et al^15^ |
| 14 | Peraliya, Sri Lanka | a) BC 2000 to 3000 | a) Tsunami event from historical texts reported from Sri Lanka | Dahanayake and Kulsena ^16^ |
| 13 | Sumatra, Indonesia | 1. 2004 tsunami 2. T2 390 ± 260 cal. BP [AD 1165-] 3. T3 630 ± 110 cal. BP [AD 1159-1480] 4. T4 740 ± 120 cal. BP [AD 1035-1415] 5. T5 820 ± 130 cal. BP [AD 975-1405] 6. T7 1080 ± 140 cal. BP [AD 672-1210] 7. T8 1220 ± 210 cal. BP [AD 406-1220] 8. T10 1500 ± 110 cal. BP [AD 260-766] 9. T11 1620 ± 140 cal. BP [AD 103-660] 10. T14 1950 ± 210 cal. BP [AD 560 -BC 430] 11. T16 2120 ± 150 cal. BP [AD 234 -BC 540] 12. T20 2520 ± 200 cal. BP [BC 1191-165] 13. T21 2750 ± 100 cal. BP [BC 1211- 771] 14. T26 3720 ± 340 cal. BP [BC 3095-1290] 15. T27 3900 ± 190 cal. BP [BC 2892-1895] 16. T29 4460 ± 150 cal. BP [BC 3631-2706] 17. T33 4630 ± 160 cal. BP [BC 3695-2921] 18. T35 4720 ± 220 cal. BP [BC 3957-2918] 19. T39 5220 ± 160 cal. BP [BC 4356-3695] 20. T40 5790 ± 140 cal. BP [BC 4979-4354] 21. T41 6030 ± 150 cal. BP [BC 5310-4588] 22. T42 6470 ± 130 cal. BP [BC 5664-5207] 23. T43 6600 ± 140 cal. BP [BC 5786-5301] | a) Evidence of turbidite from Sumatra Andaman Subduction Zone  b) Uppermost turbidite deposits, spanning ~1500 yr, are consistent with terrestrial paleoseismic/tsunami records from Thailand, Sumtra and India. | Patton et al^17^ |

**Unit-a** is present-day peaty soil (humic) comprised of moderately to well-sorted medium-fine sand (mean = 2.7ϕ; sand = 99.7%), observed from present-day back-marsh in almost all stratigraphic sections (T1, T2 and T3) except GS3 and GS5, shows a sharp contact with 2004 tsunami deposit, thickness varies from 5-10cm (Figs. 1c, 2b, 3c-d, 6; S2.1-S2.4).

**Unit-b** is yellowish medium-coarse sand exposed in almost all litho-sections: GS1, GS2, GS4, GS6, GS8-GS10, and T1-T3 (Figs. 1c, 2b, 3a, 4c-d; S2.1-S2.4). The uppermost portion of this unit in GS6 is medium sand (mean = 1.8ϕ; sand = 98.8%), middle and bottom portions are medium-fine sand (mean = 1.1 to 2.4ϕ; sand = 82.5 to 95.6%) with broken shell fragments, and shows a sharp contact with Unit-c. Inclined to horizontal stratification, laminations and bi-directional structures were observed in the middle and upper portions. **Unit-b** is thicker (~54cm) and coarser near to the coast, becomes thinner up to 5-8cm and finer towards inland. The inland geoslices (GS8-GS10) shows laminated fine-medium sand with a sharp contact (Figs. 1c, 2b and 3a, c-d; 4 c-d, 6; Supplementary Figs. S2.1-S2.4). The thicker and coarser deposits near the coast, finer and thinner inland, inverse grading, laminations, inclined-horizontal stratifications and bi-directional structures are indicative of deposition under variable velocity during inflow and back-wash by a tsunami wave. This unit represents 2004-tsunami event (Figs. 1c; 2b; 3a, c; 4c-d, 6; Supplementary Figs. S2.1-S2.4).

**Unit-c** is a peat, medium-fine sand (mean = 2.9ϕ; sand = 85.7%), exposed in GS1, GS2, GS4, GS6, GS8-GS10 as well as in T1 and T2. It developed in a coarser deposit (mean = 0.9ϕ; sand = 77.1%) near the coast as observed in GS6 and GS7 (Figs. 1c, 2b, 3a, c-d; 4 b-d; Supplementary Figs. S2.1-S2.4). Thickness varies from 5-10cm, and show sharp contact with Unit-b and a gradual contact with Unit-d. We interpret that Unit-c represents a soil unit, developed in a back-marsh environment existed during 2004 (Figs. 2b, 3a, c, 4b-d, 6; Supplementary Figs. S2.1-S2.4).

**Unit-d** is yellowish medium-coarse sand (mean = 1.4ϕ, sand = 91.3%), with a few fragments of broken shells and coral clasts. The coarser deposit was observed along the coastline in GS7 (Figs. 2b, 3b, 4b-d; Supplementary Fig. S2.3a-b), whereas, medium-coarse sand in inland litho-sections GS1, GS2, GS4, GS8-GS10 and T1-T2 (Figs. 2b, 3b-d; Supplementary Figs. S2.1-S2.4). Thickness varies from 5-20cm. We infer that this unit was deposited by the historic tsunami (Figs. 2b-4 and 6).

**Unit-e** is a peaty soil with medium-fine sand (mean = 2.4ϕ; sand = 97.3%), exposed in GS7 at a depth of ~1.10m (Figs. 2b, 3b), observed in GS1 and GS2, but not in inland sections (Figs. 2b; Supplementary Figs. S2.2-S2.4). Thickness is about 10-15cm near the coast, whereas, thins-out inland with a variable thickness from 5-8cm (Fig. 2b), shows sharp contact with underlying and overlying units.

**Unit-f is** fine silty-sand exposed in GS1, GS2, GS8-GS10, and in T1 and T3 (Figs. 2b, 3c, 4b-d; Supplementary Figs. S2.1-S2.4). Thickness varies from 8-15cm and shows gradual contact with the underlying and overlying units, represents the phase of basin-filling (Figs. 2b and 6).

**Table S2:** Grain size data of the litho-units from Geoslices – GS2, GS3, GS5, GS6, GS7, GS9, Trenches, and Beach Ridge Deposits. * Beach-ridge deposits

| **Litho-Units** | **Averaged Depth (cm)** | **Mean (φ)** | **Mode (φ)** | **Stand. Deviation (φ)** | **Gravel (%)** | **Silt (%)** | **Sand (%)** | **Grain Size (Sediment class)** |
| --- | --- | --- | --- | --- | --- | --- | --- | --- |
| Unit-a’ | 0-20 (BRD*) | 2.15 | 2.24 | 0.70 | 0.0% | 5.5% | 94.5% | Fine Sand |
| Unit-a | 0-9 | 2.79 | 2.74 | 0.57 | 0.0% | 0.3% | 99.7% | Fine sand |
| Unit-b | 9-15 | 2.47 | 2.73 | 0.92 | 4.2% | 0.2% | 95.6% | Fine Sand |
| Unit-b | 9-15 | 1.89 | 1.98 | 0.86 | 1.2% | 0.0% | 98.8% | Medium Sand |
| Unit-b | 9-15 | 1.14 | 2.74 | 1.74 | 17.3% | 0.2% | 82.5% | Medium Sand |
| Unit-c | 15-19 | 0.92 | 2.737 | 1.77 | 21.7% | 1.2% | 77.1% | Coarse Sand |
| Unit-c | 15-19 | 2.97 | 3.24 | 1.01 | 0.2% | 14.1% | 85.7% | Fine Sand |
| Unit-d | 19-24 | 1.41 | 2.74 | 1.39 | 8.1% | 0.6% | 91.3% | Medium Sand |
| Unit-e | 24-28 | 2.47 | 2.73 | 0.72 | 2.5% | 0.2% | 97.3% | Fine Sand |
| Unit-f | 28-33 | 3.16 | 2.74 | 0.76 | 0.0% | 15.3% | 84.7% | Very Fine Sand |
| Unit-g | 33-44 | 3.02 | 3.86 | 0.78 | 0.0% | 6.4% | 93.6% | Very Fine Sand |
| Unit-g | 33-44 | 1.38 | 2.73 | 1.39 | 8.2% | 0.6% | 91.2% | Medium Sand |
| Unit-h | 44-72 | 2.79 | 2.74 | 0.97 | 0.0% | 9.5% | 90.5% | Fine Sand |
| Unit-i | 72-118 | 2.20 | 3.24 | 1.46 | 3.8% | 4.6% | 91.6% | Fine Sand |
| Unit-j | 118-138 | 3.12 | 2.73 | 1.11 | 0.2% | 17.1% | 82.7% | Very Fine Sand |
| Unit-k | 138-168 | 2.66 | 3.23 | 0.82 | 0.0% | 0.2% | 99.8% | Fine Sand |
| Unit-l | 168-188 | 2.87 | 2.74 | 0.97 | 4.3% | 8.3% | 87.4% | Fine Sand |
| Unit-l | 168-188 | 1.92 | 2.73 | 1.68 | 4.3% | 6.6% | 89.1% | Medium Sand |
| Unit-m | 188-200 | 2.49 | 2.74 | 1.36 | 0.6% | 9.9% | 89.5% | Fine Sand |
| Unit-n | 200-223 | 1.79 | 2.73 | 1.58 | 13.3% | 3.9% | 82.8% | Medium Sand |
| Unit-o | 223-232 | 2.56 | 3.23 | 1.12 | 0.0% | 5.4% | 94.6% | Fine Sand |
| Unit-p | 232-256 | 1.79 | 3.24 | 1.86 | 11.1% | 1.7% | 87.2% | Medium Sand |
| Unit-q | 256-265 | 2.32 | 3.23 | 1.66 | 9.3% | 9.4% | 81.3% | Fine Sand |
| Unit-r | 265- ≥ 272 | 0.95 | 2.74 | 1.36 | 13.3% | 0.0% | 86.7% | Coarse Sand |

**Unit-g** coarse-medium sand (mean = 1.3ϕ; sand = 91.2%), observed in GS7 at a depth of ~1.35m, and was ~15cm thick (Fig. 2b, 4c-d, Supplementary Fig. S2.2-S2.4). Comprises broken shell fragments, angular coral clasts, and shows bi-directional structure (Fig. 2b, 4c-d, Supplementary Fig. S2.1-S2.4). Further inland the unit is thinner with comparatively finer grain size (mean = 3ϕ; sand = 93.6%), observed in GS1, GS2, GS8-GS10 (Fig. 2b, 4c-d, Supplementary Fig. S2.2-S2.4). Inland, it becomes relatively finer with no prominent sedimentary structure. This unit was deposited by a tsunami event (Figs. 2b and 6).

**Unit-h** is partially developed peat, comprised of fine silty-sand (mean = 2.8ϕ; sand = 90.5%), observed in GS1, GS2, GS9 and GS10 with a variable thickness of 10-20cm. It is structureless marked by a gradual contact with the underlying unit, whereas, shows sharp contact with the overlying unit i.e., Unit-g (Figs. 2b, 3d, 4c-d, 6; Supplementary Figs. S2.2-S2.4). We infer that the area was isolated from the ocean waters and remained at or above mean sea level (Figs. 2b and 6).

**Unit-i** is grayish fine silty-sand (mean = 2.2ϕ; sand = 91.6%), structureless with a sharp to gradual contact with the underlying unit, observed in GS1-GS10 except GS6 and GS7 with a variable thickness of 55-110cm, thickest in GS10 (Figs. 2b, 4c, 6; Supplementary Figs. S2.2-S2.4). Finer sediments, silt to silty-sand could be deposited under fluvial or estuarine environment or during the basin-filling. We suggest that this unit was deposited during basin-filling when the area remained submerged with a sub-tidal condition. Also considering the maximum thickness (110 cm) it could be inferred that the area remained submerged for a longer span during the inter-seismic period (Figs. 2b and 6).

**Unit-j** is peaty soil with fine sand (mean = 3.1ϕ; sand = 82.7%), shows a gradual contact with the overlying unit and a sharp with the underlying Unit-k. Observed in GS1-GS10 with thickness varying from 10-20cm (Figs. 2b and 3d, 4c-d; Supplementary Figs. S2.2-S2.4). We infer that the area was at or above mean sea level before subsidence (Figs. 2b and 6).

**Unit-k** is yellowish medium-fine sand (mean = 2.6ϕ; sand = 99.8%) with scattered fine gravels, exposed in GS1-GS5 and GS8 (Fig. 2b; Supplementary Figs. S-2.2-S2.4). Thickness varies from 14-30 cm and shows a gradual to sharp contact with underlying and overlying units. We infer that the area was under the influence of inter-tidal to sub-tidal condition (Figs. 2b and 6).

**Unit-l** is greyish brown medium-coarse sand (mean ranges from = 2.9-1.9ϕ; sand = 87.4-89.1%) with gravel comprised of coral clast and broken shells (Figs. 2b, 3d, 4a-d; Supplementary Figs. 2.2-2.4). Thickness varies from 10-40cm, and was exposed in GS3, GS5, GS8-GS10. More thickness was observed towards the ocean, becomes finer (fine sand) and thinner towards inland. In GS3, this unit was exposed at a depth of ~1.5-1.8m, whereas, in GS5 it was observed up to 2m. In GS5 Unit-l shows alternative layers of greyish medium-coarse sand with silt and fine gravel clasts comprised of broken shell and coral fragments, along with plant debris, and rip-up clasts of bedrock (Figs. 2b; 3d, 4a-d). It shows prominent inclined stratification with bi-directional structures. Also, marked by sharp contacts with Unit-m and Unit-k (Figs. 2b, 3d, 4a-d; Supplementary Figs. S2.2-S2.4). This unit was deposited during the tsunami produced by an earthquake that also resulted in coseismic subsidence as indicated by overlying finer Unit-k (Figs. 2b and 6).

**Unit-m** is peaty soil with silty-sand (mean = 2.4ϕ; sand = 89.5%), about 10-20cm thick marked by poor lamination. This unit was exposed in GS5, GS8-GS10, showing sharp contact with the underlying and overlying units (Figs. 2b, 4a-d; Supplementary Figs. S2.2-S2.4). Peaty soils are usually formed at or above mean sea level in areas isolated from the ocean. Also, peats can accumulate below the mean sea level inland if the area is isolated from the oceanic waters. Considering the study area in proximity to the ocean, we infer that the peat was formed due to a land-level change (Figs. 2b and 6).

**Unit-n** is greyish medium-coarse sand (mean = 1.7ϕ; sand = 82.8%), and was observed in GS5, GS8-GS10, exposed in deeper part at a depth of 2m (Figs. 2b, 4a-d; Supplementary Figs. S2.6, S2.9-S2.11). Thickness varies from 10-30cm and is marked by inclined laminations, with thin layers of coarser fragments comprised of broken shells. At places, rip-up clasts were also noticed in the upper portion. It shows sharp contact with underlying and overlying units. This unit was deposited by the tsunami (Figs. 2b and 5). **Unit-o,** is a thin fine silt peaty unit exposed in GS8 and GS9 only. Thickness varies from 5-8cm (Figs. 2b, 4b-c; Supplementary Figs. S2.2-S2.4), and was formed at or above mean sea level (Figs. 3b and 7).

**Unit-p** is 20-40cm thick grayish coarse sand (mean = 1.7ϕ; sand = 87.2%), with corals clasts, broken shells and rock fragments. Exposed in GS8-GS10 at a depth of ~2m, shows sharp contact with overlying and underlying units (Figs. 2b, 4c-d; Supplementary Figs. S2.2-S2.4). Gravel clasts are poorly sorted, confined in the upper portion of GS8 and in the middle part of GS9. Shows poor lamination in the upper part of GS9. Whereas, GS10 with prominent alternative sub-units is composed of whitish–grey coarse sand, blackish sand with scattered gravels and sand+gravel units. Also exhibits inverse grading in the middle portion. The coarse sediments are mainly coral clast, broken shell fragments and rock clasts. This unit is indicative of a tsunami deposit (Figs. 2b and 6). **Unit-q** is peaty unit composed of greyish fine sand with scattered gravel fragments (Figs. 2b, 4b, d, 6; Supplementary Figs. S2.2-S2.4). The thickness is about 15-20 cm, exposed only in GS10. It is marked with sharp contact with the underlying and overlying units, formed at or above mean sea level (Fig. 2b and 6). **Unit-r** is only exposed at the bottom of GS10, composed of coarse sand with broken shell fragments (Figs. 2b, 4d; Supplementary Fig. S2.1-S2.4), deposited by a tsunami event (Figs. 2b).

**S3: Paleontological studies (micro-fossil): Foraminiferal analysis**

The foraminifera analysis was carried with a standard methodology as suggested by Loeblich and Tappan^18, 19^; Scott et al ^20^; Sen Gupta^21^; Murray^22^; and Hayward, et al^23^ (Fig. S3.1 and Table S3.1). Q-mode cluster analysis was carried out using Constrained Incremental Sum of Squares (CONISS) method^24^. Along with this we also performed Fisher Alpha Diversity Index – FADI^25^. Further, to understand the foraminifera environment we also carried out Detrended Correspondence Analysis (DCA), which gave us Hierarchical Dendrograms^26^ (Fig. S3.2).

Two clusters (A and B) and hierarchical dendrograms were recognized based on the quantitative foraminifera data analysis (Figs. S3.1 and S3.2). The species assemblages in the biofacies 1 and cluster A represents a sub tidal sediment provenance and its mixing with intertidal sediments. It comprises of *Ammonia becarri, Quinqueloculina seminulam, Elphidium crispum and Rotalia sp.* (Figs. S3.1 and S3.2).

Cluster B represents an intra-tidal environment, which comprises of *Elphidium discoidale*, *Amphistegina lobifera* in Beach ridge facies. Whereas, at 350m inland Cluster A shows a sub tidal species assemblage of *Elphidium crispum*, *Amphistegina lobifera Quinqueloculina seminulam, Rotalia sp.* and Cluster B shows an intertidal environment predominated by species of *Ammonia beccarii* (Figs. S3.1 and S3.2). This suggests mixing of intertidal and sub tidal sediment sources at various proportion and its deposition in 350m inland. Paleotsunami deposit (Unit-n) biofacies I show majority of sub tidal foraminifera with minor amount of intertidal species, which are suggestive of a deeper sediment provenance (Figs. S3.1 and S3.2b). Paleotsunami deposit (Unit-p) biofacies II shows major intertidal foraminifera with minor number of subtidal foraminifera suggest a shallow sediment provenance.

**Table S3.1:** Foraminifera species and their ecology found in Badabalu, Southern Andaman.

| **Sl. No** | **Names of Foraminiferal Species** | **Test Composition and Ecology** | **Reference** |
| --- | --- | --- | --- |
| 1 | *Ammonia beccarii* | Calcareous, infaunal, free; muddy sand; marginal marine, water depth 0 to 50 m | Murray^22^ |
| 2 | *Quinqueloculina seminulam* | Porcelaneous, epifaunal on firm substrates and on sediment; infaunal down to 10cm. Marginal marine; water depth 0 to 2564 m. | Murray^22^; Hayward et al^23^ |
| 3 | *Rotalia sp.* | Calcareous, infaunal, marginal marine;  Water depth 32 m to 529 m. | Hayward et al^23^; |
| 4 | *Amphistegina lobifera* | Amphistegina: epifaunal, free; coral reefs, lagoons; water depth 0.3 m to 452 m | Murray^22^; Hayward et al^23^ |
| 5 | *Elphidium crispum* | Calcareous, non-keeled species, infaunal, free; mud, sand, Inner shelf, water depth  32 m to 529 m | Hayward et al^223^ |
| 6 | *Elphidium discoidale* | Calcareous, non-keeled species, infaunal, free; mud, sand; marginal marine; water depth 3 m to 145 m | Murray^22^; Hayward et al^23^ |

All litho-units (Unit-a - youngest and Unit-r - oldest) were analysed. However, Units a, f, h, i, j, k, l, and m did not show presence of any foraminifera assemblages (Fig. S2.3). Possibly the preservation potential is based on the environmental influence, dissolution activity, bioturbation, etching and reworking^22, 27, 28, 29^. In most of the older deposits, preservation potential for calcareous tests is poor due to the low pH existing below the surface^30^. Sediment units analysed from the geoslice GS2, GS3 and GS5 at inland did not contain any foraminifera expect the bottom-most layer of GS5, Uint n (Fig. S2.3).

**Unit-b** (2004 tsunami deposit) shows good preservation and high abundance of foraminifera (110 individuals per 10gm) in the beach ridge facies, which were dominantly pristine (70-50%) (Figs. S3.1-3.3, Table S3.2). The *Elphidium crispum* was dominant within the foraminiferal assemblage. The foraminiferal tests with size >250 µm exhibit significant abrasion. FADI varies from 0.58 to 0.83 from top to bottom, with a similarity index of 0.7- 0.9. Q-mode cluster analysis shows very close clustering (Fig. S3.4). Detrended Correspondence Analysis (DCA) and Hierarchical Dendrograms suggest that these species belong to **Biofacies I** - indicative of subtidal sediments associated with the tsunami (Fig. S3.2). Inland sections did not contain any foraminifera. Although, thin veneers of 2004 tsunami deposit (Unit-b) exposed in 350m inland (Trench-TS1) did not contain any foraminifera, it yielded dinoflagallate *Scrippsiella spinifera* (500 µm). This dinoflagallate species are very common in the seawater column, with a wide distribution through sub-arctic, temporal and tropical oceanic regions^31^.

**Unit-c** represents pre-2004 tsunami peaty soil. Only geoslice GS6 yield a very few (74 individuals per 10gm) foraminifera (Fig. S3.3a). The *Elphidium discoidale* was dominant in the foraminiferal assemblage. Q-mode cluster analysis suggested a coarse clustering in this unit. FAD index was 0.15. DCA and the resulting Hierarchical Dendrograms as well as similarity index of -0.16 suggests that these species belong to **Biofacies II** – sourced from intertidal sediments, attributed to inter-tidal/marshy environment (Figs. S3.2 and S3.3a)

**Unit-d** represents paleo-tsunami deposit. The higher percentage of foraminifera (245 individuals per 10gm) was observed in geoslice GS7 collected from the beach ridge (Table S3.2). The *Elphidium crispum* was dominant. The foraminifera size >250 μm shows significant abrasion (42%) in the test. Q-mode cluster analysis shows a very coarse clustering (Fig. S3.3a). Based on the FAD index of 0.58, Detrended Correspondence Analysis (DCA), Hierarchical Dendrograms and similarity index ranging from 0.7-0.9, these species were classified under **Biofacies I** -indicative of subtidal sediments (Fig. S3.2).

**Unit-e** represents pre-2004 tsunami deposit. Comparatively less concentration (96 individuals per 10gm) of foraminifera were found from the geoslice section GS7 collected from the present-day beach ridge (Figs. S3.2 and S3.3b). However, inland sections did not yield any foraminifera. In this unit also the *Elphidium discoidale* was dominant. Q-mode analysis shows a coarse clustering. Based on FAD index of 1.11, Detrended Correspondence Analysis (DCA), Hierarchical Dendrograms and similarity index of -0.16, these species were classified under **Biofacies II** – indicative of intertidal sediment source attributed to the marshy environment (Figs. S3.2 and S3.3b).

**Unit-g** represents paleo-tsunami deposit. Higher concentration of foraminifera was observed from geoslice section GS7, however, inland sections not show foraminiferal preservation (Fig.S3.3b). In total 222 number of foraminifera per 10gm were identified; about 42% were pristine. The *Elphidium crispum* was dominant in the assemblage. Considering the FAD index of 0.58, Q-mode clustering showing very coarse clustering, similarity index varying from 0.7-0.9 as well as Detrended Correspondence Analysis (DCA) and Hierarchical Dendrograms these species are classified as **Biofacies I –** indicative of subtidal sediments associated with the tsunami (Figs. S3.2 and S3.3b).

**Unit-n** corresponds to the tsunami deposit identified at 350m inland at a depth of about 180 cm. High concentration of foraminifera and good preservation potentials (177 individuals per 10gm), which were dominantly abraded (90%) (Fig. S3.1). *Ammonia beccarii* dominate the foraminiferal assemblage (Fig. S3.5). FAD index of 0.58 and Q-mode clustering analysis showing coarse cluster, similarity index of 1, Detrended Correspondence Analysis (DCA) and Hierarchical Dendrograms suggests a mixing of intertidal (**Biofacies I**), where majority of subtidal sediments mix with little intertidal sediments and deposition at 350m inland due to near-source tsunami (Figs. S3.2 and S3.3b).

**Unit-p** represents tsunami deposit found inland. High abundance of foraminifera (63 individuals per 10gm) was observed. About 88% were dominantly abraded. The *Elphidium crispum* dominate the foraminiferal assemblage. FAD index of 1.11, Q-mode analysis showing coarse clustering, similarity index of -1, Detrended Correspondence Analysis (DCA) and Hierarchical Dendrograms suggests minor mixing of subtidal sediments (**Biofacies II**) with major intertidal sediments due to the tsunami and deposited at inland (Figs. S3.2 and S3.3b).

Unit-l, Unit-n and Unit-p are the thicker tsunami deposit observed inland with broken coral and shell fragments. From these units, only Unit-n and Unit-p show intermixing of deep subtidal sediments with intertidal/marshy sediments due to the tsunami. Typically, these species are abraded, fragmented; suggest large sediment transport and water depth due to near-source mega-earthquake and associated transoceanic tsunami.

**Table S3.2:** Formaninferal taxonomy and Taphonomy for beach ridge facies (GS6, and GS7) and 350 m inland section (GS9, GS2, GS3 and GS5). n/a. = Foraminifera not available

| Foraminiferal taxonomy | | | | | Foraminiferal taphonomy | | |
| --- | --- | --- | --- | --- | --- | --- | --- |
| Units | Depth in  Unit (cm) | Total counts Per 10g | % sub tidal  species | % intertidal  species | % Abraded | % Fragmented | %Pristine |
| Beach ridge facies | | | | | | | |
| Unit a’ | 0-20 | n/a | n/a | n/a | n/a | n/a | n/a |
| Unit b | 20-31 | 110 | 96 | 4 | 30 | 20 | 50 |
| Unit b | 60-67 | 71 | 94 | 6 | 35 | 25 | 40 |
| Unit c | 84-90 | 74 | 0 | 100 | 0 | 0 | 100 |
| Unit d | 90-100 | 245 | 92 | 8 | 42 | 38 | 20 |
| Unit e | 111-130 | 96 | 75 | 25 | 0 | 0 | 100 |
| Unit g | 130-150 | 222 | 100 | 0 | 52 | 30 | 18 |
| 350 m Inland section (GS9) | | | | | | | |
| Unit a | 0-8 | n/a | n/a | n/a | n/a | n/a | n/a |
| Unit b | 8-11 | n/a | n/a | n/a | n/a | n/a | n/a |
| Unit c | 11-14 | n/a | n/a | n/a | n/a | n/a | n/a |
| Unit d | 14-16 | n/a | n/a | n/a | n/a | n/a | n/a |
| Unit f | 16-30 | n/a | n/a | n/a | n/a | n/a | n/a |
| Unit g | 30-40 | n/a | n/a | n/a | n/a | n/a | n/a |
| Unit h | 40-70 | n/a | n/a | n/a | n/a | n/a | n/a |
| Unit i | 70-150 | n/a | n/a | n/a | n/a | n/a | n/a |
| Unit j | 150-172 | n/a | n/a | n/a | n/a | n/a | n/a |
| Unit k | 172-190 | n/a | n/a | n/a | n/a | n/a | n/a |
| Unit l | 190-200 | n/a | n/a | n/a | n/a | n/a | n/a |
| Unit m | 200-210 | n/a | n/a | n/a | n/a | n/a | n/a |
| Unit n | 210-220 | 177 | 15 | 85 | 90 | 10 | 0 |
| Unit o | 222-232 | n/a | n/a | n/a | n/a | n/a | n/a |
| Unit p | 260-270 | 63 | 82 | 8 | 88 | 12 | 0 |
| 350 m Inland section (GS2, GS3 and GS5) | | | | | | | |
| Unit a | 0-8 | n/a | n/a | n/a | n/a | n/a | n/a |
| Unit b | 8-11 | n/a | n/a | n/a | n/a | n/a | n/a |
| Unit c | 11-28 | n/a | n/a | n/a | n/a | n/a | n/a |
| Unit d | 28-31 | n/a | n/a | n/a | n/a | n/a | n/a |
| Unit e | 31-34 | n/a | n/a | n/a | n/a | n/a | n/a |
| Unit f | 34-47 | n/a | n/a | n/a | n/a | n/a | n/a |
| Unit g | 47-58 | n/a | n/a | n/a | n/a | n/a | n/a |
| Unit h | 58-72 | n/a | n/a | n/a | n/a | n/a | n/a |
| Unit i | 72-124 | n/a | n/a | n/a | n/a | n/a | n/a |
| Unit j | 124-135 | n/a | n/a | n/a | n/a | n/a | n/a |
| Unit k | 135-151 | n/a | n/a | n/a | n/a | n/a | n/a |
| Unit l | 151-195 | n/a | n/a | n/a | n/a | n/a | n/a |
| Unit m | 195-205 | n/a | n/a | n/a | n/a | n/a | n/a |
| Unit n | 205-212 | 537 | 77 | 23 | 60 | 20 | 20 |
| Dianoflagallate 350 m Inland section (Trench 1) Fig. S3.3 | | | | | | | |
| Unit b | 10-12 | 7 | -- | -- | -- | -- | -- |

**S4: Dating**

Four sediment samples were dated by Optical Stimulated Luminescence (OSL) dating technique at IIT Kanpur, and 18 samples were dated for ^14^C (AMS) ages at Beta Analytic, USA, as well as at Inter University Accelerator Center (IUAC), New Delhi (Figs. S4.1-S4.3). Ages were calibrated and modelled with Bayesian analysis in the program OxCal v.4.2.4 to get calendar ages and events^32, 33, 34^ (Fig. S4.2; Supplementary Table S4.1).

*S4a: OSL Dating*

Optical Stimulated Luminescence dating (OSL) technique involves the measurement of the luminescence signal, which was stored in the crystal lattice of sand grains after the burial^35^. To obtain the age, the OSL signals, stored in the light-sensitive traps of the quartz grains, must be reset to zero by exposure to the sunlight either during erosion, transportation or deposition^36^. Therefore, OSL measures the elapsed time since the last exposure to sunlight^37^. Earthquakes generated tsunami waves are best known to carry deep-seated sediments to the direct contact with the sunlight. Therefore, dating these tsunamigenic sediments provides the first-hand information about the tsunami events^38^. The standard, SAR-OSL protocol has been applied to date the tsunami events worldwide, for example the large 1755 tsunami event in Algarve coast, Portugal^39^, 2004 Indian Ocean tsunami at Phra Thong Island, Thailand^38^ and Killalea lagoon, southeast coast of Australia^40^. However, two major problems are encountered while dating the tsunami deposits: (i) it has been observed that turbulent water-lain tsunami deposits show incomplete resetting of the luminescence signals, which leads to overestimation of the sediment ages^41, 42, 43^, (ii) Another limitation encountered while dating young tsunamigenic sediments are their dim signals due to incomplete bleaching^44^.

*Sample Preparation and Measurement*

We collected sediment samples from the exposed trenches as well as geoslices obtained from Badabalu site (Figs. S2.1-S2.11; Table S4.2). For paleodose measurement, samples were treated with 1N HCl for one hour followed by washing the sample at-least three times with de-ionized water. It was followed by treatment with 30% H_2_O_2_ until all the effervescence disappears followed by washing again with de-ionized water. This is done to get rid of carbonates and organic matter from the sediments. Dried samples were then sieved to obtain 90-212 um grain fractions of which only 90-125 um fraction size was used for further analysis. The quartz and feldspar were isolated with the help of Frantz magnetic separator with constant current of 1.50 A. Then the isolated quartz was etched with 40% Hydrofluoric acid (HF) solution for 60 minutes to remove outer alpha skin and dissolve any leftover feldspar. The isolated quartz was then rinsed with HCL to get rid of any fluorite precipitate from HF acid.

| **Table S4.1:** ^14^C Accelerator Mass Spectrometry (AMS) ages from Badabalu. | | | | | | | | | | |
| --- | --- | --- | --- | --- | --- | --- | --- | --- | --- | --- |
| **Sr. No.** | **Sample site Geoslice/ Trench no.** | **Sample no.** | **Dating Lab number^a^** | **Sedimentary Unit** | **Depth in cm** | **Material** | **δ13C(‰)‡** | **^14^C age yrs BP** | **Calendar age (2σ) from OxCal** | **Probability Range of age (2σ)** |
| 1 | TS2 | TS2Ca | 383139 | c | 15 | Charcoal | -27.3 | 102.4 +/- 0.3 pMC | Modern |  |
| 2 | GS9 | JNMGS9C1 | IUACD#16C239 | h | 44 | Buried wood |  | 3235±547 | **BC 3033 -348** | 95.4% probability  3089BC (0.3%) 3052BC  **3033BC (94.2%) 348BC**  317BC (0.9%) 208BC  68.2% probability  2274BC (0.6%) 2256BC  2208BC (67.6%) 833BC |
| 3 | GS3 | JNMGS3a | 383134 | j | 125 | Coconut clast | -26.6 | 180 ± 30 | **AD 1652 -1917** | 95.4% Probability  **1652 (19.1%) 1696 AD**  **1726 (51.9%) 1815 AD**  **1836 (4.1%) 1877 AD**  **1917 AD (20.4%)** |
| 4 | GS3 | JNMGS3b | IUACD#16C237 | j | 130 | Root |  | 376±49 | **AD 1442-1636** | 95.4% probability  **1442AD (95.4%) 1636AD**  68.2% probability  1449AD (43.8%) 1522AD  1575AD (4.7%) 1585AD  1590AD (19.7%) 1625AD |
| 5 | GS9 | JNMGS9C2 | IUACD#16C226 | j | 148 | Charcoal |  | 368±45 | **AD 1446-1636** | 95.4% probability  **1446AD (95.4%) 1636AD** 1454AD (40.6%) 1522AD  1575AD (27.6%) 1625AD |
| 6 | GS3 | JNMGS3c | 383135 | l | 154 | Buried wood | - 27.2 | 340 ± 30 | **AD 1470- 1640** | 95.4% Probability  **1470 (95.4%) 1640AD**  68.2% probability  1490AD (23.2%) 1525AD  1557AD (30.7%) 1602AD  1610AD (14.3%) 1632AD |
| 7 | GS3 | JNMGS3d | IUACD#16C238 | l | 163 | Buried wood |  | 592±58 | **AD 1286- 1426** | 95.4% probability  **1286AD (95.4%) 1426AD**  68.2% probability  1304AD (49.6%) 1364AD  1384AD (18.6%) 1407AD |
| 8 | GS5 | JNMGS5C3 | 331288 | l | 174 | Buried wood | - 27.2 | 390 ± 30 | **AD 1441- 1631** | **1441 (63.3%) 1524 AD**  **1559 (0.5%) 1562 AD**  **1571 (25.6%) 1631 AD** |
| 9 | GS5 | JNMGS5C2 | IUACD#16C223 | l | 182 | Charcoal |  | 421±46 | **AD 1416- 1632** | 95.4% probability  **1416AD (73.3%) 1524AD**  **1557AD (22.1%) 1632AD**  68.2% probability  1430AD (59.7%) 1494AD  1601AD (8.5%) 1616AD |
| 10 | GS5 | JNMGS5C1 | 411660 | m | 196 | Buried wood | -25.9 | 1530 ± 30 | **AD 425-600** | 95.4% Probability  **428 (95.4%) 599AD** |
| 11 | GS9 | JNMGS9C3 | IUACD#16C227 | m | 190 | Charcoal |  | 456±46 | **AD 1329-1620** | 95.4% probability  **1329AD (0.9%) 1340AD**  **1396AD (89.2%) 1521AD**  **1591AD (5.3%) 1620AD**  68.2% probability  1415AD (68.2%) 1466AD |
| 12 | GS5 | JNMGS5C5 | 331287 | n | 204 | Buried wood | -26.8 | 3590 ± 30 | **BC 2028-1884** | 95.4% Probability  **2028 (95.4%) 1884BC** |
| 13 | GS5 | JNMFORBB1 | 411658 | n | 208 | Foraminifera | +0.1 | 3740 ± 30 | **BC 2275-2035** | 95.4% Probability  **2275 (81.6%) 2122BC**  **2093 (13.8%) 2035BC** |
| 14 | GS10 | JNMGS10C1 | IUACD#16C229 | n | 216 | Charcoal |  | 3720±49 | **BC 2284-1972** | 95.4% probability  **2284BC (5.0%) 2247BC**  **2234BC (90.4%)**  **1972BC**  68.2% probability 2198BC (15.0%) 2166BC  2150BC (18.7%) 2111BC  2104BC (34.5%) 2036BC |
| 15 | GS8 | JNMGS8C2 | IUACD#16C225 | o | 222 | Charcoal |  | 2828±319 | **BC 1876-211** | 95.4% probability  **1876BC (0.5%) 1840BC**  **1823BC (0.4%) 1795BC**  **1782BC (93.3%) 352BC**  297BC (1.1%) 228BC  221BC (0.1%) 211BC  68.2% probability  1446BC (62.6%) 748BC |
| 16 | GS8 | JNMGS8C1 | IUACD#16C224 | p | 260 | Charcoal |  | 4286±49 | **BC 3082- 2704** | 95.4% probability **3082BC (1.1%) 3068BC**  **3026BC (85.3%) 2860BC**  **2808BC (7.7%) 2756BC**  **2720BC (1.3%) 2704BC**  68.2% probability  3010BC (10.5%) 2980BC  2940BC (57.7%) 2874BC |
| 17 | GS9 | JNMGS9C4 | IUACD#16C228 | p | 228 | Charcoal |  | 4639±51 | **BC 3630- 3137** | 95.4% probability  **3630BC (5.6%) 3582BC**  **3533BC (87.7%) 3335BC**  **3210BC (1.3%) 3192BC**  **3152BC (0.9%) 3137BC**  68.2% probability  3512BC (54.7%) 3424BC  3383BC (13.5%) 3360BC |
| 18 | GS9 | JNMGS9C5 | IUACD#16C240 | p | 272 | Buried wood |  | 6528±72 | **BC 5621-5359** | 95.4% probability  **5621BC (95.4%) 5359BC** |

*^a^Samples processing and measurements were carried out at Beta Analytics, USA and IUAC, New Delhi.*

*^b^“Calibrated” or calendar ages were calculated using “CALIB rev 5.01” and calibration curves (IntCal04, Reimer et al^32, 33^).*

*† “Corrected pMC (percent modern carbon)" indicates the percent of modern (1950) carbon corrected for fractionation using the 13C measurement.*

*‡The Conventional Radiocarbon Age represents the Measured Radiocarbon Age corrected for isotopic fractionation, calculated using δ^13^C. ∆R correction of 50 yr has been applied to sample no. JNMFORBB1 (Dutta et al^34^).*

After drying, the sample was re-sieved to remove < 90 um to acquire fine pure quartz grains. These grains were then mounted on 9.8 mm diameter stainless steel aliquots with the help of silicon spray. All the processing was carried out in the laboratory controlled red light environment. For the paleodose determination, Riso TL/OSL reader with an EMI 9635Q photomultiplier and two 3 mm Hoya U-340 filters were used.

*D_e_ Determination and Ages*

For all the samples, 24 aliquots were prepared and measured using SAR protocol for which 40-60% aliquots pass the SAR criteria. The protocol includes five dose points, comprising three regenerative doses, a zero dose and one recycling dose^45^. The quartz decay curve with a peak value at (0-0.4s) i.e., the fast component and late background signals (23-40s) were used for all the measurements. The fast component signal of the decay curve is suitable for estimating the equivalent dose because they are rapidly bleached by sunlight and less susceptible to thermal transfer of charge^45, 46^. For ***De*** (Equivalent Dose) calculation, dose-response data i.e., Lx/Tx was fitted with an exponential function and natural dose was extrapolated from the regenerative doses.

Obtaining accurate ages is the ultimate aim of the dating protocol. The tsunamigenic sediments are known to contain a mix of grains, of which some are well bleached and some remained under a cover of water and bleached insufficiently. The equivalent dose measured in these samples will be largely due to the residual dose and results in over-estimation of the ages^47^. To overcome this problem several age models have been proposed to obtain the best single value of the equivalent dose^47, 48^. The Minimum Age Model (MAM) is the most reliable age models used for the partially bleached samples^47^ and Central Age Model (CAM) is the most widely accepted model to determine the equivalent dose. Selection of the age models depends primarily upon the over-dispersion (OD), which is the observed spread in the estimated De values^49^. If OD is more than 40% the MAM age model is used whereas CAM is used^47^.

Due to the observed over-dispersed De in sample TSTL1 and TS3TL1, the MAM age model was used. Although sample TSTL1 provided the most reliable result of the 2004 tsunami event, the TS3TL1 gave an overestimated age with an offset of 20-40 years. Similar results of residual charge equivalent to <50 years have been observed in the 2004 tsunami deposits from India^41^, 60-120 years from Lisbon 1755 tsunami^39^ and 20-40 years from Thailand^38^.

Buried wood and charcoal samples from Unit-p gave 14C AMS ages of 6528±72 yr (BCE 5621-5359; JNMGS9C5); 4639±51 (BCE 3630-3137; JNMGS9C4) and 4286±49 yr (BCE 3082-2704; JNMGS8C1). A charcoal from Unit-o yielded an age of 2828±319 yr (BCE 1876-211; JNMGS8C2). Charcoal and foraminifera from Unit-n gave AMS ages of 3590±30 yr (BCE 2028-1884; JNMGS5C5), 3720±49 (BCE 2284-1972; JNMGS10C1) and 3740±30 yr (BCE 2275-2035; JNMFORBB1), respectively. A buried wood and charcoal from the Unit-m gave AMS ages of 1530±30 yr (CE 425-600; JNMGS5C1) and 456±46 yr (CE 1329-1620; JNMGS9C3), respectively. Three buried wood, and one charcoal sample from Unit-l yielded 340±30 yr (CE 1470-1640; JNMGS3c), 592±58 yr (CE 1286-1426; JNMGS3d), 390±30 yr (CE 1441-1631; JNMGS5C3) and 421±46 yr (CE 1416-1632; JNMGS5C2), respectively. Three samples from the Unit-j were dated as 180±30 yr (CE 1652-1917; JNMGS3a), 376±49 yr (CE 1442-1636; JNMGS3b), and 368±45 yr (CE 1446-1636; JNMGS9C2). A charcoal sample from the Unit-h was dated as 3235±547 yr (BCE 3033-348; JNMGS9C1). Sediment sample collected from Unit-g yielded an OSL age of 205±35 yr (CE 1739-1850; GS10a). Sample collected from Unit-d from trench T1 gave an OSL age of 155±11 yr (CE 1838-1883; TSTL2). Whereas, a charcoal from Unit-c from trench T2 gave a modern age of 102.4±0.3 pMC (TS2Ca). The samples from Unit-b from trenches TS1 and TS3 gave OSL ages of 11±2 yr (CE 1999-2007; TSTL1) and 39±5 yr (CE 1965-1985; TS3TL1) respectively.

**Table S4.2:** Optical Stimulation Luminescence (OSL) ages from Badabalu.

| **Sr. No.** | **Sample site Geoslice/ Trench no.** | **Sample no.** | **Material** | **Depth in cm** | **Sedime-ntary Unit** | **Water saturation** | **U(ppm)** | **Th(ppm)** | **K(%)** | **De** | **Dose Rate** | **^a^Age in BP** | **^b^Age in CE/BCE**  **OxCal Cal.** |
| --- | --- | --- | --- | --- | --- | --- | --- | --- | --- | --- | --- | --- | --- |
| 1 | TS1 | TSTL1 | Fine-sand | 17 | b | 2.99 ±0.06 | 0.348±0.004 | 1.127±0.012 | 3.004± 0.02 | 0.036 ± 0.006 | 3.28±0.04 | 11±2 | **CE 1999-2007** |
| 2 | TS1 | TSTL2 | Fine-sand | 35 | d | 0.58 ±0.05 | 0.158±0.002 | 0.580±0.005 | 1.686± 0.012 | 0.304±0.02 | 1.96±0.03 | 155±11 | **CE 1838-1883** |
| 3 | TS3 | TS3TL1 | Medium-coarse sand | 70 | b | 1.3 ±0.14 | 0.039±0.006 | 0.323 ±0.003 | 0.857 ±0.02 | 0.042±0.009 | 1.09±0.03 | 39±5 | **CE 1965-1985** |
| 4 | GS10 | GS10a | Fine-sand | 34 | g | 1.61 ±0.16 | 0.206±0.005 | 0.902±0.010 | 2.215 ±0.02 | 0.51±0.087 | 2.491±0.03 | 205±35 | **CE 1747-1850** |

*^a^Samples processing and measurements were carried out at Indian Institute of Technology Kanpur.*

*^b^“Calibrated” or calendar ages were calculated using “OxCal” program.*

*S4b: OxCal modelled ages*

All ages obtained from 14C AMS and OSL were modelled to get calendar ages and probability distributions of stratigraphy exposed in geoslices and trenches excavated along WNW-ESE transect at Badabalu (Fig. S4.2). The ages are calculated and modelled using OxCal version 4.2.4 (Ramsey et al 2013^50^) and IntCal13 atmospheric curves (Reimer et al., 2013^51^). We infer at least eight events (earthquakes/tsunamis) those occurred in last 8000 yrs. Event I represents 2004 tsunami; Event II was around CE 1777-1883, could be correlated with CE 1881 earthquake and tsunami from Car Nicobar. Event III occurred during CE 1674-1821, could be correlated with CE 1762 earthquake/tsunami reported from Arakan Suduction Zone. Event IV was around CE 1485-1610, correlated with CE 1672 reported from Andaman Island. Event V was around CE 1325-1434, correlated with CE 1300-1400 earthquake and tsunami from Andaman, Thailand and Indonesia. Event VI was around BCE 2480-2060, could be correlated with a tsunami reported from southeast Sri Lanka that occurred during BCE 2000-3000. Event VII occurred during BCE 2966-2286, correlated with tsunami event that occurred between 4500 cal. BP and 5000 cal. BP reported from southeast Sri Lanka, and also with the event of 5357-5575 cal. BP reported from Indonesia. Event VIII occurred before BCE 5600, correlated with event that occurred during 7324-7529 cal. BP reported from Indonesia. Conventional radiocarbon ages and other relevant details are summarized in Tables S4.1 and S4.2. Areas with white outlines indicate the probability distributions functions (PDFs) of calibrated radiocarbon ages. Gray areas represent posterior PDFs. Brackets below each PDF are 2σ uncertainties.

**S5: Geochemical analysis**

Several studies have attempted to differentiate tsunami deposits globally from non-tsunami deposits in terms of geochemical signatures^52, 53, 54, 55, 56^. Elevated concentrations of major and trace elements (e.g., Ca, Na, Mg, Ba, and Sr), selected metals (Cr, Cu, Ni, Co, Pb, Zn), as well as elemental ratios (Na/K, Ca/K, Ba/Rb, Sr/Rb, Ca/Rb, Ca/Ti) of coexisting sediments within a single core sample have been used to identify tsunami deposits indicated by the specific geochemical signatures expected from marine influence (Table S5.1; Figs. 5 & S5.1)^52, 56, 57, 58^. On the other hand, terrigenous deposits that are mainly derived from the crustal sources are characterized by varying abundances of Fe_2_O_3_, SiO_2_, Al_2_O_3_, MgO, TiO_2_ different than the marine sediments; some of these signatures are affected by prolonged burial, leaching, and minor contamination by marine sediments ^59, 60^. Consequently, these major oxides are relatively depleted in the tsunami deposits reported from different parts of the world^54, 61, 62, 63^. Particularly, tsunami deposits are more enriched in CaO, Na_2_O, and MnO contents and depleted in SiO_2_, Fe_2_O_3_, Al_2_O_3_, and K_2_O relative to the terrigenous deposits− the characteristic tsunami signature is due to contribution from shells, oceanic salinity or their authigenic nature (components of oceanic inorganic minerals that precipitate directly from the seawater, either in the water column or in the sediment after burial) including hydrogenous Fe-Mn oxyhydroxides. These forms either coating on existing minerals or occur as nodules and crusts generally below in the deepest part of slope or an abyssal plain. The higher concentrations of Ca, Sr, and Mg in coarser tsunami deposits compared to overlying or underlying crustal sediment deposits have been attributed to the presence of biogenic sediments such as calcareous shells and shell-hashes^64, 65, 66^. Abundant corals are also commonly found in tsunami deposits. Marine sediments scoured by high-energy tsunami waves when mixed with coastal sediments may show enhanced concentrations of Ti, Zr and Th^67^. However, it is possible that all these geochemical signatures may not be well preserved in coarse tsunami deposits because of hydrodynamic size fractionation and removal of clay material^68^.

To examine the geochemical signatures of the near-surface coastal stratigraphy from Badabalu site, we analysed 16 samples from 17 litho-units (Units a to q, except from Unit-o) for major oxides and selected trace element abundances (Table S5.1; Figs. 5 and S5.1). Major oxides abundances were determined using a wavelength dispersive X-ray Fluorescence Spectrometry (WD-XRF, Rigaku ZSX Primus II) and trace element concentrations determined using an Inductively Coupled Plasma Mass Spectrometer (ICP-MS, Thermo Fisher Scientific iCAPQ) at IIT Kanpur. Detailed analytical procedure is given by Chandra et al. (2017)^69^. XRF analyses were carried out on fusion glass beads and ICP-MS analyses were done on samples digested using HF-HNO_3_ mixture. Based on three repetitive measurements of geo-standards (LKD-2, SBC-1, WGB-1, AGV-2) analysed with unknown samples, the uncertainty associated with the major elements is <5% and that for the trace elements is within the range of 3-10%.

**Table S5.1: Geochemical composition of the tsunami (TS) and non-tsunami sediments (NS) of Andaman Islands. Major oxides in weight % (XRF data) and trace elements (ICP-MS data) in ppm. Eu/Eu* = Eu_CN_/(Sm_CN_×Gd_CN_)^0.5^, Ce/Ce*=Ce_CN_/(La_CN_*Nd_CN_)^0.5^; CN is chondrite normalization after McDonough and Sun (1995) ^70^.**

| **Nature** | **NS** | **TS** | **NS** | **TS** | **NS** | **NS** | **TS** | **NS** | **NS** | **NS** | **NS** | **TS** | **NS** | **TS** | **TS** | **NS** |
| --- | --- | --- | --- | --- | --- | --- | --- | --- | --- | --- | --- | --- | --- | --- | --- | --- |
| **Major Elements** | **Unit-a** | **Unit-b** | **Unit-c** | **Unit-d** | **Unit-e** | **Unit-f** | **Unit-g** | **Unit-h** | **Unit-i** | **Unit-j** | **Unit-k** | **Unit-l** | **Unit-m** | **Unit-n** | **Unit-p** | **Unit-q** |
| **SiO2** | 70.4 | 79.6 | 66.8 | 42.9 | 58.9 | 75.6 | 31.1 | 73.1 | 65.4 | 62.3 | 64.1 | 58.8 | 62.3 | 63.6 | 58.3 | 59.5 |
| **Al2O3** | 10.9 | 3.9 | 12.1 | 2.1 | 2.6 | 8.1 | 1.7 | 10.1 | 10.3 | 14.1 | 11.8 | 14.8 | 15.1 | 12.9 | 10.2 | 10.4 |
| **TiO2** | 0.8 | 0.4 | 0.8 | 0.3 | 0.3 | 0.6 | 0.2 | 0.7 | 0.7 | 0.9 | 0.8 | 0.9 | 0.9 | 0.8 | 0.7 | 0.7 |
| **Fe2O3** | 2.7 | 0.9 | 2.9 | 1.1 | 1.1 | 2.5 | 0.8 | 2.4 | 2.7 | 5.6 | 3.6 | 7.2 | 5.3 | 4.4 | 4.7 | 4.1 |
| **MgO** | 1.1 | 0.3 | 1.3 | 1.1 | 0.9 | 0.9 | 1.2 | 1.1 | 1.2 | 1.6 | 1.3 | 1.8 | 1.8 | 1.4 | 1.6 | 1.9 |
| **MnO** | 0.02 | 0.02 | 0.03 | 0.05 | 0.03 | 0.02 | 0.05 | 0.02 | 0.02 | 0.03 | 0.03 | 0.04 | 0.04 | 0.03 | 0.04 | 0.04 |
| **CaO** | 1.7 | 6.7 | 0.1 | 29.8 | 6.1 | 1.7 | 15.3 | 1.7 | 0.2 | 1.7 | 1.7 | 6.7 | 1.7 | 1.8 | 6.4 | 5.5 |
| **K2O** | 1.3 | 0.6 | 1.4 | 0.3 | 0.4 | 1.1 | 0.2 | 1.2 | 1.3 | 1.7 | 1.4 | 2.1 | 1.9 | 1.8 | 1.5 | 1.4 |
| **Na2O** | 1.4 | 0.8 | 1.5 | 1.6 | 2.2 | 1.2 | 1.5 | 1.5 | 1.5 | 1.3 | 1.5 | 1.1 | 1.3 | 1.1 | 0.9 | 1.1 |
| **P2O5** | 0 | 0 | 0.01 | 0.01 | 0 | 0.01 | 0.00 | 0.00 | 0.02 | 0.04 | 0.05 | 0.09 | 0.03 | 0.00 | 0 | 0.01 |
| **TOTAL** | 90.3 | 93.2 | 86.9 | 78.9 | 72.4 | 91.7 | 52.1 | 91.7 | 83.3 | 89.1 | 86.2 | 93.5 | 90.3 | 87.9 | 84.4 | 84.6 |
| **LOI** | 9.7 | 6.8 | 13.1 | 21.1 | 27.6 | 8.3 | 47.9 | 8.3 | 16.7 | 10.9 | 13.8 | 6.5 | 9.7 | 12.1 | 15.6 | 15.4 |
| **Trace Elements** | | | | | | | | | | | | | | | | |
| **Sc** | 192 | 94.2 | 6.1 | 3.3 | 79.8 | 21.8 | 154 | 16.9 | 31.4 | 48.4 | 52.8 | 220 | 138 | 26.3 | 112 | 14.6 |
| **Mn** | 80.1 | 34.8 | 37.9 | 123 | 83.6 | 69.6 | 96.5 | 85.5 | 135 | 112 | 76.4 | 176 | 111 | 90.7 | 173 | 197 |
| **V** | 59.2 | 12.8 | 21.9 | 11.7 | 13.7 | 35.3 | 65.5 | 43.8 | 78.1 | 69.6 | 32.1 | 89.3 | 72.1 | 56.3 | 58.4 | 62.0 |
| **Cr** | 78.1 | 27.8 | 31.4 | 20.8 | 40.5 | 53.7 | 91.8 | 74.1 | 98.4 | 82.6 | 42.3 | 117 | 83.3 | 69.3 | 86.1 | 70.8 |
| **Co** | 20.3 | 15.6 | 2.5 | 3.3 | 16.7 | 11.9 | 26.1 | 16.2 | 21.4 | 18.4 | 13.8 | 28.7 | 21.8 | 14.1 | 20.7 | 13.7 |
| **Ni** | 35.1 | 11.8 | 5.4 | 6.8 | 21.1 | 27.3 | 46.2 | 34.1 | 51.4 | 45.2 | 22.8 | 68.9 | 47.9 | 32.3 | 46.4 | 42.8 |
| **Cu** | 22.9 | 11.5 | 6.2 | 2.1 | 10.7 | 14.4 | 25.1 | 16.6 | 24.1 | 21.1 | 14.1 | 29.7 | 24.3 | 18.4 | 19.1 | 16.8 |
| **Zn** | 69.5 | 120 | 7.1 | 1.1 | 46.1 | 47.5 | 1808 | 688 | 83.9 | 782 | 116 | 106 | 140 | 63.9 | 60.1 | 60.2 |
| **Rb** | 53.7 | 15.8 | 12.6 | 3.5 | 13.5 | 34.8 | 55.5 | 45.9 | 64.5 | 55.6 | 29.3 | 70.5 | 58.4 | 51.5 | 49.7 | 51.1 |
| **Sr** | 78.6 | 42.1 | 18.3 | 566 | 552 | 56.5 | 82.9 | 61.5 | 82.3 | 72.2 | 48.1 | 91.7 | 75.3 | 72.1 | 644 | 745 |
| **Ba** | 275 | 127 | 52.8 | 15.1 | 102 | 161 | 270 | 169 | 253 | 227 | 129 | 335 | 245 | 172 | 206 | 191 |
| **Cs** | 6.3 | 4.6 | 0.5 | 0.1 | 4.3 | 3.8 | 8.0 | 4.9 | 6.7 | 5.5 | 4.1 | 8.4 | 6.7 | 4.9 | 5.9 | 4.2 |
| **Y** | 35.2 | 12.8 | 7.6 | 17.8 | 10.8 | 21.0 | 23.0 | 17.4 | 19.8 | 17.6 | 12.5 | 26.1 | 20.0 | 15.1 | 18.8 | 15.1 |
| **Zr** | 1684 | 1324 | 51.3 | 211 | 1073 | 871 | 1792 | 942 | 1178 | 920 | 851 | 1930 | 1271 | 757 | 1186 | 603 |
| **Nb** | 14.6 | 7.3 | 6.4 | 2.9 | 5.6 | 8.9 | 15.1 | 10.8 | 12.9 | 11.7 | 8.3 | 17.4 | 11.8 | 10.3 | 10.1 | 9.5 |
| **Hf** | 37.5 | 29.4 | 1.5 | 4.6 | 24.1 | 19.2 | 38.3 | 21.3 | 25.9 | 20.9 | 18.7 | 42.7 | 28.6 | 17.7 | 27.0 | 13.3 |
| **Ga** | 13.5 | 7.1 | 3.7 | 1.9 | 6.7 | 8.7 | 15.2 | 10.6 | 15.3 | 13.3 | 8.4 | 17.5 | 14.8 | 10.9 | 11.3 | 10.1 |
| **La** | 50.1 | 18.1 | 21.1 | 16.1 | 14.1 | 25.4 | 36.7 | 39.9 | 33.2 | 33.1 | 17.4 | 47.6 | 32.3 | 25.7 | 27.7 | 26.3 |
| **Ce** | 168 | 96.4 | 44.4 | 36.3 | 79.1 | 81.3 | 149 | 118 | 116 | 107 | 74.1 | 178 | 119 | 84.2 | 115 | 81.7 |
| **Pr** | 10.6 | 3.9 | 5.1 | 4.5 | 3.4 | 5.6 | 7.9 | 8.2 | 7.3 | 7.5 | 4.1 | 9.8 | 6.9 | 5.8 | 6.4 | 6.1 |
| **Nd** | 39.7 | 15.1 | 18.8 | 18.3 | 13.3 | 21.7 | 29.9 | 30.7 | 28.1 | 28.1 | 16.3 | 37.2 | 26.3 | 22.8 | 25.1 | 23.9 |
| **Sm** | 7.9 | 2.9 | 3.8 | 3.6 | 2.6 | 4.2 | 6.1 | 6.1 | 5.8 | 5.7 | 3.4 | 7.4 | 5.3 | 4.7 | 5.2 | 4.8 |
| **Eu** | 1.7 | 0.7 | 1.1 | 0.8 | 0.7 | 1.1 | 1.5 | 1.1 | 1.5 | 1.4 | 0.8 | 2.1 | 1.4 | 1.2 | 1.4 | 1.3 |
| **Gd** | 8.2 | 3.6 | 4.2 | 3.5 | 3.1 | 4.8 | 6.3 | 6.3 | 6.5 | 6.1 | 3.8 | 7.9 | 5.4 | 4.9 | 5.4 | 4.9 |
| **Tb** | 1.2 | 0.5 | 0.5 | 0.4 | 0.5 | 0.7 | 1.1 | 1.1 | 1.1 | 0.9 | 0.6 | 1.3 | 0.9 | 0.8 | 0.9 | 0.8 |
| **Dy** | 4.6 | 2.1 | 2.4 | 1.4 | 1.8 | 2.8 | 4.3 | 3.7 | 4.1 | 3.6 | 2.4 | 5.1 | 3.8 | 3.1 | 3.6 | 3.1 |
| **Ho** | 0.9 | 0.5 | 0.5 | 0.2 | 0.4 | 0.6 | 0.9 | 0.7 | 0.8 | 0.7 | 0.5 | 1.1 | 0.8 | 0.6 | 0.7 | 0.5 |
| **Er** | 3.3 | 1.9 | 1.5 | 0.9 | 1.6 | 2.0 | 3.3 | 2.5 | 3.1 | 2.6 | 1.8 | 3.8 | 2.8 | 2.2 | 2.7 | 2.1 |
| **Tm** | 0.4 | 0.3 | 0.2 | 0.1 | 0.2 | 0.2 | 0.4 | 0.3 | 0.4 | 0.3 | 0.2 | 0.5 | 0.4 | 0.3 | 0.3 | 0.2 |
| **Yb** | 2.8 | 1.9 | 1.3 | 0.6 | 1.6 | 1.8 | 3.1 | 2.2 | 2.7 | 2.3 | 1.8 | 3.7 | 2.7 | 2.1 | 2.4 | 1.8 |
| **Lu** | 0.5 | 0.3 | 0.2 | 0.1 | 0.3 | 0.3 | 0.5 | 0.4 | 0.5 | 0.4 | 0.3 | 0.6 | 0.4 | 0.3 | 0.4 | 0.3 |
| **CaO/TiO_2_** | 2.18 | 17.8 | 0.08 | 97.5 | 18.8 | 2.82 | 69.1 | 2.59 | 0.26 | 1.95 | 2.27 | 7.67 | 1.93 | 2.23 | 9.73 | 8.21 |
| **Sr/Ba** | 0.29 | 0.33 | 0.35 | 37.6 | 5.43 | 0.35 | 0.31 | 0.36 | 0.33 | 0.32 | 0.37 | 0.27 | 0.31 | 0.42 | 3.13 | 3.91 |
| **Na/K** | 1.09 | 1.28 | 1.07 | 6.22 | 5.17 | 1.16 | 6.43 | 1.20 | 1.19 | 0.72 | 1.02 | 0.54 | 0.67 | 0.55 | 0.62 | 0.66 |
| **Eu/Eu*** | 0.65 | 0.66 | 0.76 | 0.69 | 0.75 | 0.75 | 0.75 | 0.55 | 0.75 | 0.73 | 0.68 | 0.80 | 0.80 | 0.76 | 0.81 | 0.82 |
| **Ce/Ce*** | 1.62 | 2.55 | 1.00 | 1.02 | 2.63 | 1.53 | 1.96 | 1.44 | 1.67 | 1.54 | 2.00 | 1.81 | 1.77 | 1.55 | 1.96 | 1.47 |
| **Nature** | **NS** | **TS** | **NS** | **TS** | **NS** | **NS** | **TS** | **NS** | **NS** | **NS** | **NS** | **TS** | **NS** | **TS** | **TS** | **NS** |
| **Major Elements** | **Unit-a** | **Unit-b** | **Unit-c** | **Unit-d** | **Unit-e** | **Unit-f** | **Unit-g** | **Unit-h** | **Unit-i** | **Unit-j** | **Unit-k** | **Unit-l** | **Unit-m** | **Unit-n** | **Unit-p** | **Unit-q** |
| **SiO_2_** | 70.4 | 79.6 | 66.8 | 42.9 | 58.9 | 75.6 | 31.1 | 73.1 | 65.4 | 62.3 | 64.1 | 58.8 | 62.3 | 63.6 | 58.3 | 59.5 |
| **Al_2_O_3_** | 10.9 | 3.9 | 12.1 | 2.1 | 2.6 | 8.1 | 1.7 | 10.1 | 10.3 | 14.1 | 11.8 | 14.8 | 15.1 | 12.9 | 10.2 | 10.4 |
| **TiO_2_** | 0.8 | 0.4 | 0.8 | 0.3 | 0.3 | 0.6 | 0.2 | 0.7 | 0.7 | 0.9 | 0.8 | 0.9 | 0.9 | 0.8 | 0.7 | 0.7 |
| **Fe_2_O_3_** | 2.7 | 0.9 | 2.9 | 1.1 | 1.1 | 2.5 | 0.8 | 2.4 | 2.7 | 5.6 | 3.6 | 7.2 | 5.3 | 4.4 | 4.7 | 4.1 |
| **MgO** | 1.1 | 0.3 | 1.3 | 1.1 | 0.9 | 0.9 | 1.2 | 1.1 | 1.2 | 1.6 | 1.3 | 1.8 | 1.8 | 1.4 | 1.6 | 1.9 |
| **MnO** | 0.02 | 0.02 | 0.03 | 0.05 | 0.03 | 0.02 | 0.05 | 0.02 | 0.02 | 0.03 | 0.03 | 0.04 | 0.04 | 0.03 | 0.04 | 0.04 |
| **CaO** | 1.7 | 6.7 | 0.1 | 29.8 | 6.1 | 1.7 | 15.3 | 1.7 | 0.2 | 1.7 | 1.7 | 6.7 | 1.7 | 1.8 | 6.4 | 5.5 |
| **K_2_O** | 1.3 | 0.6 | 1.4 | 0.3 | 0.4 | 1.1 | 0.2 | 1.2 | 1.3 | 1.7 | 1.4 | 2.1 | 1.9 | 1.8 | 1.5 | 1.4 |
| **Na_2_O** | 1.4 | 0.8 | 1.5 | 1.6 | 2.2 | 1.2 | 1.5 | 1.5 | 1.5 | 1.3 | 1.5 | 1.1 | 1.3 | 1.1 | 0.9 | 1.1 |
| **P_2_O_5_** | 0 | 0 | 0.01 | 0.01 | 0 | 0.01 | 0.00 | 0.00 | 0.02 | 0.04 | 0.05 | 0.09 | 0.03 | 0.00 | 0 | 0.01 |
| **TOTAL** | 90.3 | 93.2 | 86.9 | 78.9 | 72.4 | 91.7 | 52.1 | 91.7 | 83.3 | 89.1 | 86.2 | 93.5 | 90.3 | 87.9 | 84.4 | 84.6 |
| **LOI** | 9.7 | 6.8 | 13.1 | 21.1 | 27.6 | 8.3 | 47.9 | 8.3 | 16.7 | 10.9 | 13.8 | 6.5 | 9.7 | 12.1 | 15.6 | 15.4 |
| **Trace Elements** | | | | | | | | | | | | | | | | |
| **Sc** | 192 | 94.2 | 6.1 | 3.3 | 79.8 | 21.8 | 154 | 16.9 | 31.4 | 48.4 | 52.8 | 220 | 138 | 26.3 | 112 | 14.6 |
| **Mn** | 80.1 | 34.8 | 37.9 | 123 | 83.6 | 69.6 | 96.5 | 85.5 | 135 | 112 | 76.4 | 176 | 111 | 90.7 | 173 | 197 |
| **V** | 59.2 | 12.8 | 21.9 | 11.7 | 13.7 | 35.3 | 65.5 | 43.8 | 78.1 | 69.6 | 32.1 | 89.3 | 72.1 | 56.3 | 58.4 | 62.0 |
| **Cr** | 78.1 | 27.8 | 31.4 | 20.8 | 40.5 | 53.7 | 91.8 | 74.1 | 98.4 | 82.6 | 42.3 | 117 | 83.3 | 69.3 | 86.1 | 70.8 |
| **Co** | 20.3 | 15.6 | 2.5 | 3.3 | 16.7 | 11.9 | 26.1 | 16.2 | 21.4 | 18.4 | 13.8 | 28.7 | 21.8 | 14.1 | 20.7 | 13.7 |
| **Ni** | 35.1 | 11.8 | 5.4 | 6.8 | 21.1 | 27.3 | 46.2 | 34.1 | 51.4 | 45.2 | 22.8 | 68.9 | 47.9 | 32.3 | 46.4 | 42.8 |
| **Cu** | 22.9 | 11.5 | 6.2 | 2.1 | 10.7 | 14.4 | 25.1 | 16.6 | 24.1 | 21.1 | 14.1 | 29.7 | 24.3 | 18.4 | 19.1 | 16.8 |
| **Zn** | 69.5 | 120 | 7.1 | 1.1 | 46.1 | 47.5 | 1808 | 688 | 83.9 | 782 | 116 | 106 | 140 | 63.9 | 60.1 | 60.2 |
| **Rb** | 53.7 | 15.8 | 12.6 | 3.5 | 13.5 | 34.8 | 55.5 | 45.9 | 64.5 | 55.6 | 29.3 | 70.5 | 58.4 | 51.5 | 49.7 | 51.1 |
| **Sr** | 78.6 | 42.1 | 18.3 | 566 | 552 | 56.5 | 82.9 | 61.5 | 82.3 | 72.2 | 48.1 | 91.7 | 75.3 | 72.1 | 644 | 745 |
| **Ba** | 275 | 127 | 52.8 | 15.1 | 102 | 161 | 270 | 169 | 253 | 227 | 129 | 335 | 245 | 172 | 206 | 191 |
| **Cs** | 6.3 | 4.6 | 0.5 | 0.1 | 4.3 | 3.8 | 8.0 | 4.9 | 6.7 | 5.5 | 4.1 | 8.4 | 6.7 | 4.9 | 5.9 | 4.2 |
| **Y** | 35.2 | 12.8 | 7.6 | 17.8 | 10.8 | 21.0 | 23.0 | 17.4 | 19.8 | 17.6 | 12.5 | 26.1 | 20.0 | 15.1 | 18.8 | 15.1 |
| **Zr** | 1684 | 1324 | 51.3 | 211 | 1073 | 871 | 1792 | 942 | 1178 | 920 | 851 | 1930 | 1271 | 757 | 1186 | 603 |
| **Nb** | 14.6 | 7.3 | 6.4 | 2.9 | 5.6 | 8.9 | 15.1 | 10.8 | 12.9 | 11.7 | 8.3 | 17.4 | 11.8 | 10.3 | 10.1 | 9.5 |
| **Hf** | 37.5 | 29.4 | 1.5 | 4.6 | 24.1 | 19.2 | 38.3 | 21.3 | 25.9 | 20.9 | 18.7 | 42.7 | 28.6 | 17.7 | 27.0 | 13.3 |
| **Ga** | 13.5 | 7.1 | 3.7 | 1.9 | 6.7 | 8.7 | 15.2 | 10.6 | 15.3 | 13.3 | 8.4 | 17.5 | 14.8 | 10.9 | 11.3 | 10.1 |
| **La** | 50.1 | 18.1 | 21.1 | 16.1 | 14.1 | 25.4 | 36.7 | 39.9 | 33.2 | 33.1 | 17.4 | 47.6 | 32.3 | 25.7 | 27.7 | 26.3 |
| **Ce** | 168 | 96.4 | 44.4 | 36.3 | 79.1 | 81.3 | 149 | 118 | 116 | 107 | 74.1 | 178 | 119 | 84.2 | 115 | 81.7 |
| **Pr** | 10.6 | 3.9 | 5.1 | 4.5 | 3.4 | 5.6 | 7.9 | 8.2 | 7.3 | 7.5 | 4.1 | 9.8 | 6.9 | 5.8 | 6.4 | 6.1 |
| **Nd** | 39.7 | 15.1 | 18.8 | 18.3 | 13.3 | 21.7 | 29.9 | 30.7 | 28.1 | 28.1 | 16.3 | 37.2 | 26.3 | 22.8 | 25.1 | 23.9 |
| **Sm** | 7.9 | 2.9 | 3.8 | 3.6 | 2.6 | 4.2 | 6.1 | 6.1 | 5.8 | 5.7 | 3.4 | 7.4 | 5.3 | 4.7 | 5.2 | 4.8 |
| **Eu** | 1.7 | 0.7 | 1.1 | 0.8 | 0.7 | 1.1 | 1.5 | 1.1 | 1.5 | 1.4 | 0.8 | 2.1 | 1.4 | 1.2 | 1.4 | 1.3 |
| **Gd** | 8.2 | 3.6 | 4.2 | 3.5 | 3.1 | 4.8 | 6.3 | 6.3 | 6.5 | 6.1 | 3.8 | 7.9 | 5.4 | 4.9 | 5.4 | 4.9 |
| **Tb** | 1.2 | 0.5 | 0.5 | 0.4 | 0.5 | 0.7 | 1.1 | 1.1 | 1.1 | 0.9 | 0.6 | 1.3 | 0.9 | 0.8 | 0.9 | 0.8 |
| **Dy** | 4.6 | 2.1 | 2.4 | 1.4 | 1.8 | 2.8 | 4.3 | 3.7 | 4.1 | 3.6 | 2.4 | 5.1 | 3.8 | 3.1 | 3.6 | 3.1 |
| **Ho** | 0.9 | 0.5 | 0.5 | 0.2 | 0.4 | 0.6 | 0.9 | 0.7 | 0.8 | 0.7 | 0.5 | 1.1 | 0.8 | 0.6 | 0.7 | 0.5 |
| **Er** | 3.3 | 1.9 | 1.5 | 0.9 | 1.6 | 2.0 | 3.3 | 2.5 | 3.1 | 2.6 | 1.8 | 3.8 | 2.8 | 2.2 | 2.7 | 2.1 |
| **Tm** | 0.4 | 0.3 | 0.2 | 0.1 | 0.2 | 0.2 | 0.4 | 0.3 | 0.4 | 0.3 | 0.2 | 0.5 | 0.4 | 0.3 | 0.3 | 0.2 |
| **Yb** | 2.8 | 1.9 | 1.3 | 0.6 | 1.6 | 1.8 | 3.1 | 2.2 | 2.7 | 2.3 | 1.8 | 3.7 | 2.7 | 2.1 | 2.4 | 1.8 |
| **Lu** | 0.5 | 0.3 | 0.2 | 0.1 | 0.3 | 0.3 | 0.5 | 0.4 | 0.5 | 0.4 | 0.3 | 0.6 | 0.4 | 0.3 | 0.4 | 0.3 |
| **CaO/TiO_2_** | 2.18 | 17.8 | 0.08 | 97.5 | 18.8 | 2.82 | 69.1 | 2.59 | 0.26 | 1.95 | 2.27 | 7.67 | 1.93 | 2.23 | 9.73 | 8.21 |
| **Sr/Ba** | 0.29 | 0.33 | 0.35 | 37.6 | 5.43 | 0.35 | 0.31 | 0.36 | 0.33 | 0.32 | 0.37 | 0.27 | 0.31 | 0.42 | 3.13 | 3.91 |
| **Na/K** | 1.09 | 1.28 | 1.07 | 6.22 | 5.17 | 1.16 | 6.43 | 1.20 | 1.19 | 0.72 | 1.02 | 0.54 | 0.67 | 0.55 | 0.62 | 0.66 |
| **Eu/Eu*** | 0.65 | 0.66 | 0.76 | 0.69 | 0.75 | 0.75 | 0.75 | 0.55 | 0.75 | 0.73 | 0.68 | 0.80 | 0.80 | 0.76 | 0.81 | 0.82 |
| **Ce/Ce*** | 1.62 | 2.55 | 1.00 | 1.02 | 2.63 | 1.53 | 1.96 | 1.44 | 1.67 | 1.54 | 2.00 | 1.81 | 1.77 | 1.55 | 1.96 | 1.47 |

**Supplementary Figures:**


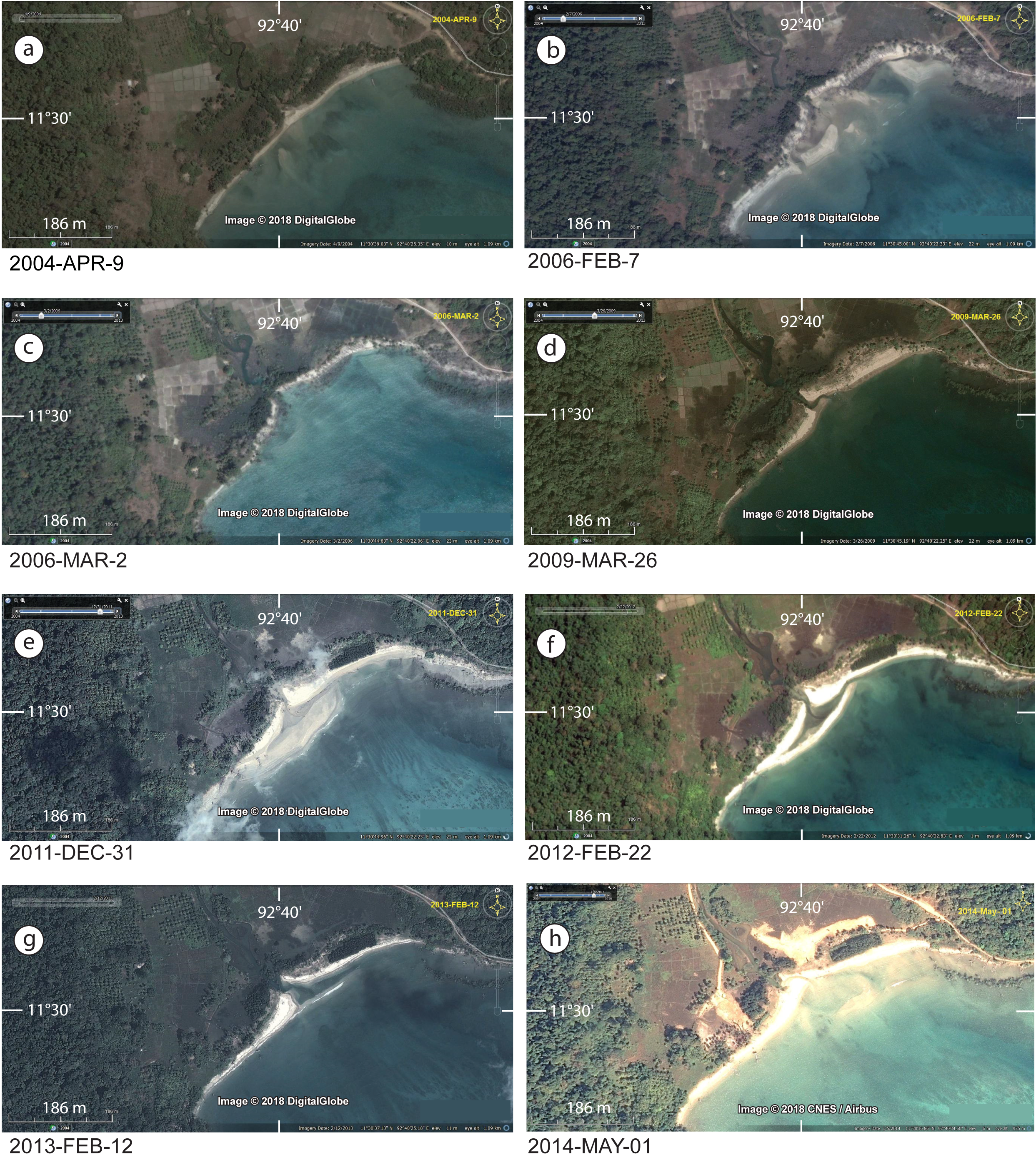


**Fig. S1.1 (a-h):** Google Earth images showing changes in the landscape along the south coast of Andaman Island at Badabalu caused by 2004 Sumatra-Andaman earthquake. We interpreted pre and post-earthquake images from April 2004 to May 2014. The land subsidence was ~40-45cm, which resulted in inundation and erosion of the coastline as well as inland migration of beach and beach-ridge. [Figs. S1.1a-g: Map data: *Google, DigitalGlobe*], and [S1.1h: *Map data Google, CNES/Airbus*].

**
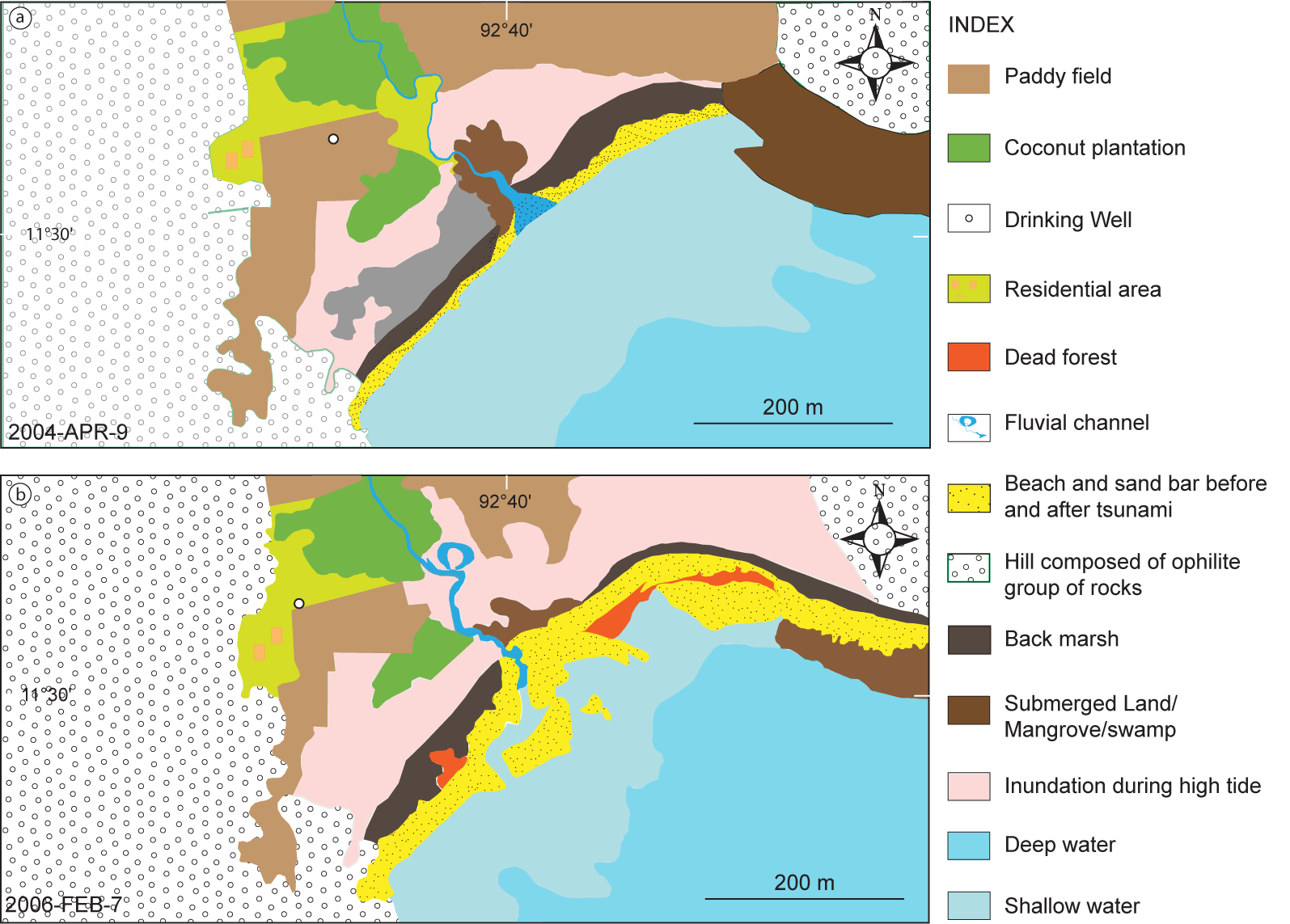
**

**Fig. S1.2: (a)** Generalized geomorphic map showing the distribution of coastal landforms around Badabalu during 09 April 2004, based on onformation extracted from Google Earth image (refer Fig. S1.1a). **(b)** Map showing prominent geomorphic changes caused by 2004 Sumatra-Andaman earthquake and tsunami. Landform information extracted from Google Earth image taken on 07 February 2006 (refer Fig. S1.1b). Coseismic subsidence resulted in the landward shifting of beach by 35-50 m, causing inundation and formation of beach-ridge as well as back-marsh inland. The land-level change also affected the stream debouching into the bay. The stream had a well-marked 3-6m wide meandering channel before the 2004 earthquake. Morphology of the channel changed from meandering to straight, also marked by the formation of an Ox-bow lake and widening of channel up to 8-14m. (*Geomorphic features in Figs. S1.2 a and b were traced from Google Earth images using Adobe illustrator Version CS3. Prepared by JNM & FCJ*)


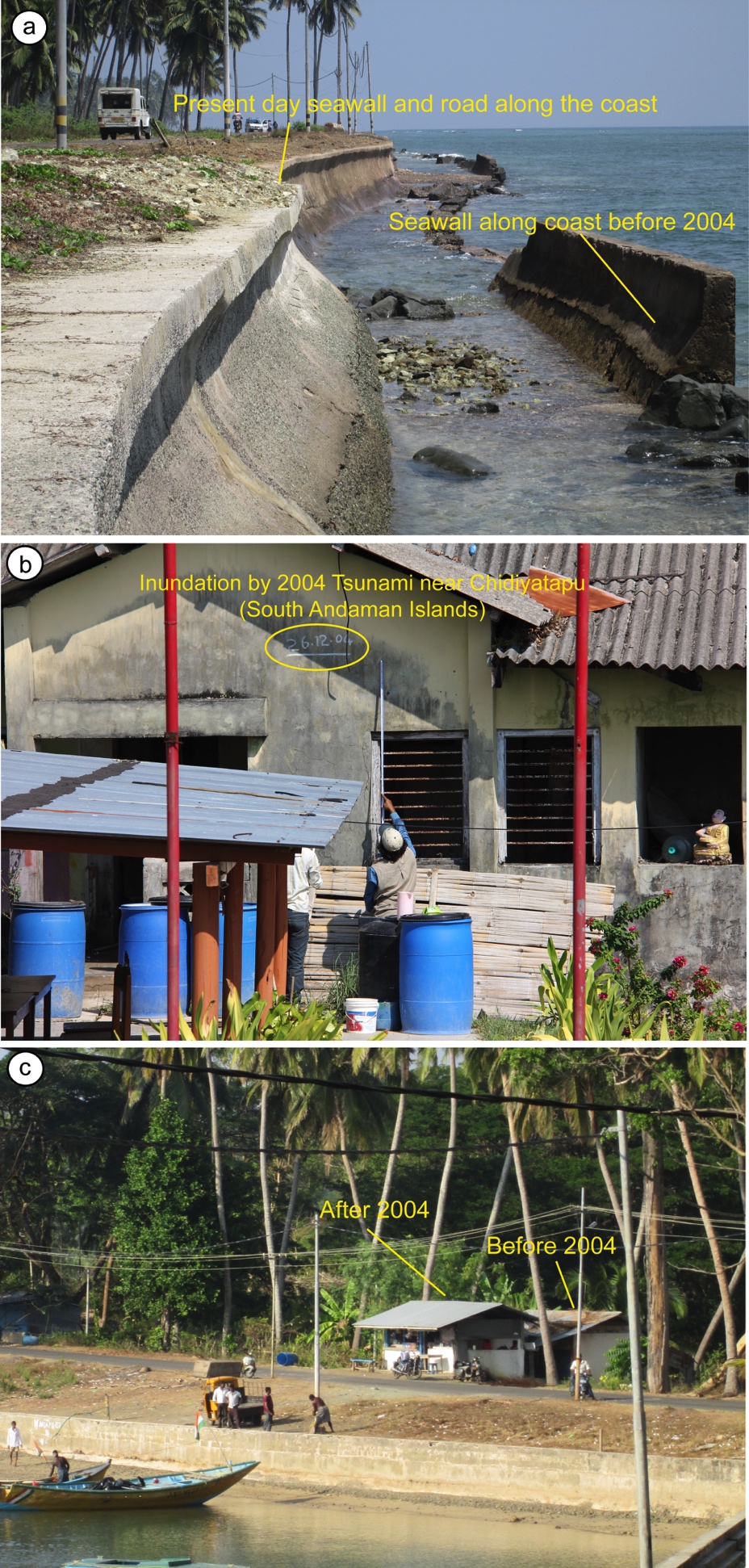


**Fig. S1.3: (a)** Field photograph showing a damaged seawall due to 0.5m subsidence along the southeast coast of Andaman Island during the 2004 Sumatra-Andaman earthquake, which was later reconstructed at a higher level. The photo was taken on the way to Chidiyatapu. **(b)** Submergence of the area by ~3 m (indicated by water-mark) during the 2004 tsunami near Chidiyatapu. The tsunami height was about 3-3.5 m. **(c)** Reconstructed local house on the reclaimed elevated areas to avoid inundation during high tide. Photo near Chidiyatapu along the south coast of Andaman. [All photos taken by JNM].


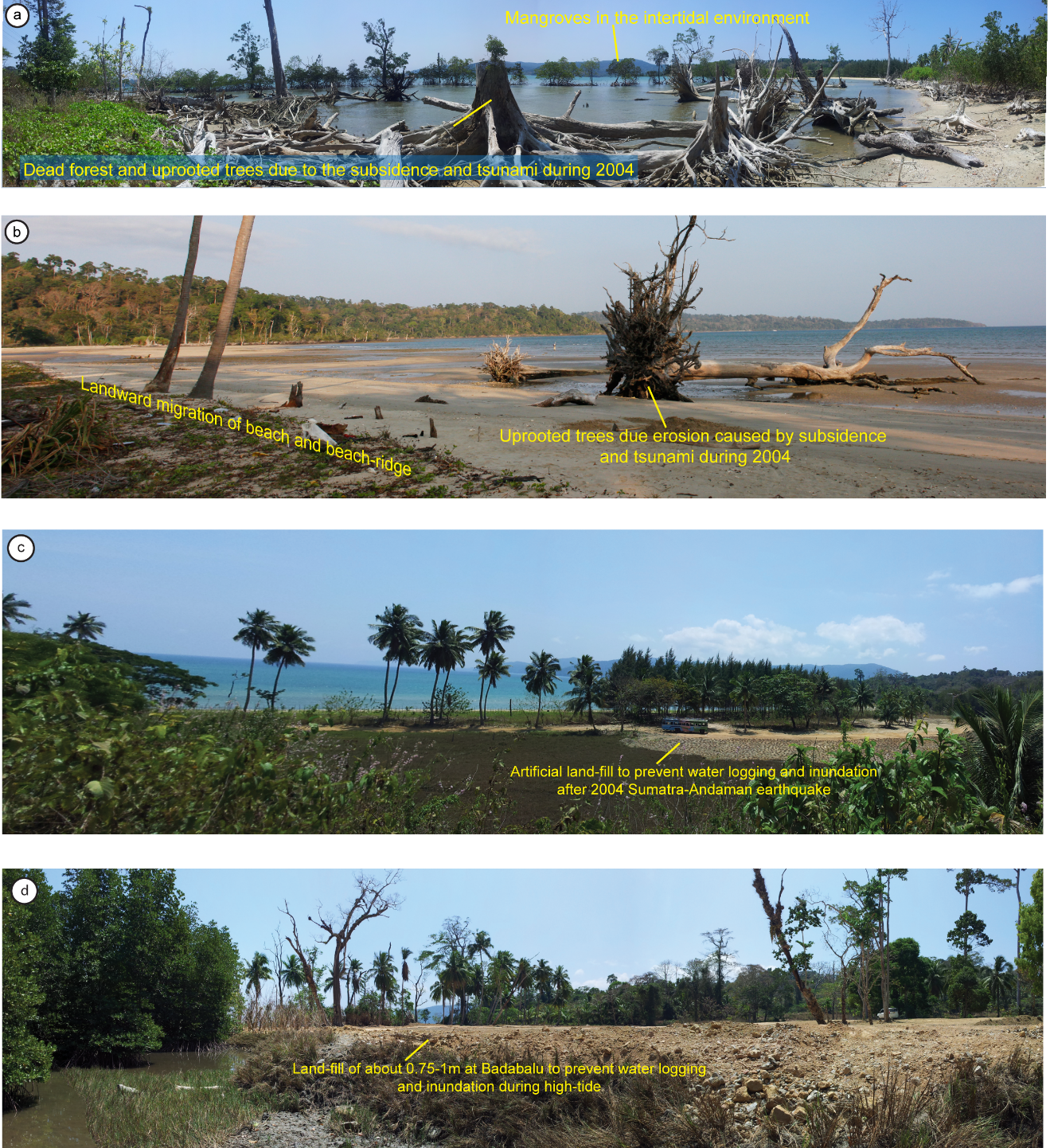


**Fig. S1.4: (a)** Formation of dead forest due to the intrusion of saline water and regular inundation resulted due to subsidence along the southern coast of Andaman Island. **(b)** Landward migration of beach and beach-ridge observed at Badabalu. The coastline is subjected to coastal erosion due to subsidence and tsunami. (Photos looking east at Badabalu beach). **(c)** Artificial land-fill by local residents to prevent waterlogging and inundation from high-tide near Badabalu (Photo looking south). **(d)** The fill was up to 0.75-1.0 m along the south coast of Andaman Islands at Badabalu (Photo looking south). [All photos taken by JNM].


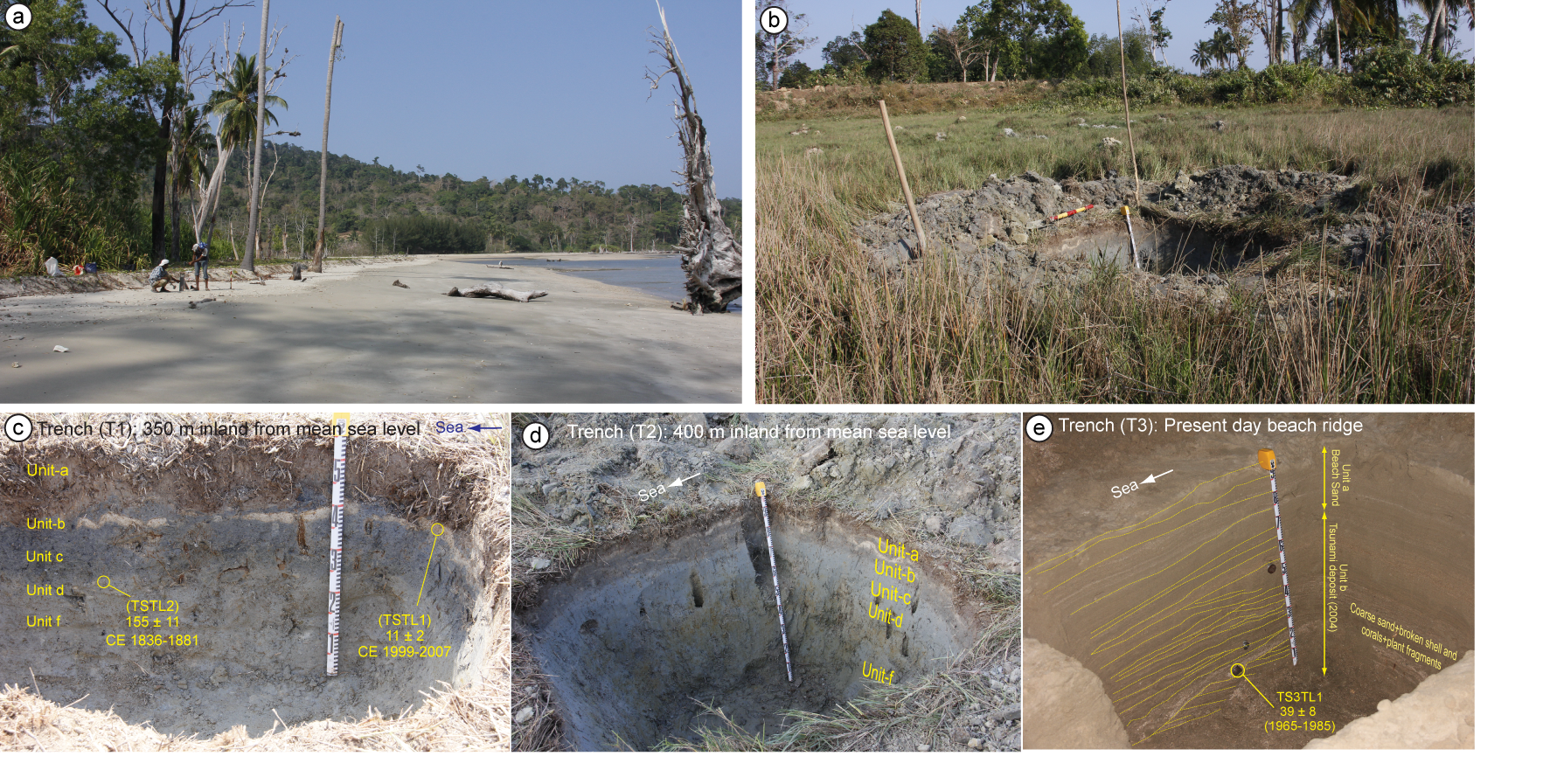


**Fig. S2.1**: **(a)** Geoslice section collected from the present-day beach at Badabalu. Sampling was carried out keeping in mind that the present-day beach area was occupied by a back-marsh before 2004. Please refer Figure 2a-b for location. **(b)** E-W oriented trenches (T2& T3) excavated behind the present-day beach-ridge in the back-marsh area at Badabalu. Please refer Figure 3a-b for location. **(c)** T1 and T2 trenches exposed along a transect behind the beach ridge in the back-marsh area. The near-surface stratigraphic section reveals present day peaty soil and discontinuous sand layers of 2004 tsunami deposit. Please refer Figure 3a-b for location. **(d)** E-W oriented trench (T3) excavated on the present-day beach. The lower portion of T3 trench shows a thick 2004 tsunami deposit comprised of coarse to medium sand with bi-directional structures capped by fine sand of present-day beach at Badabalu. Please refer Figure 3a-b for location. [All photos taken by JNM].


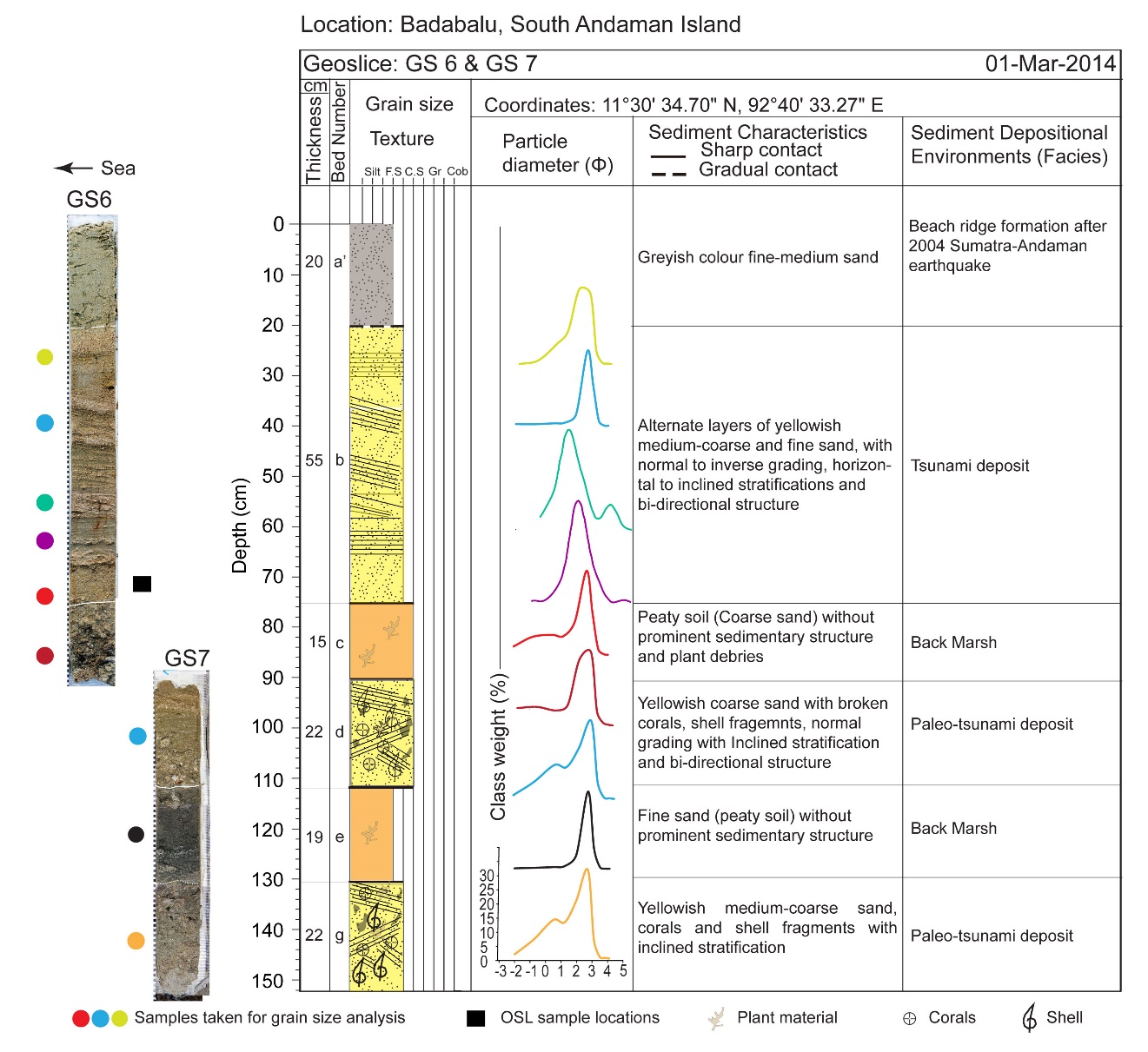


**Fig. S2.2:** Geoslice sections GS6 and GS7 collected from the present-day beach-ridge at Badabalu. Geoslice section GS6 show present-day beach-ridge deposit, about 55 cm thick medium-coarse sand representing 2004 tsunami deposit. The lower portion comprised of a peaty unit. Further deeper section exposed in GS7 show two coarser units sand separated by the peaty unit. The coarser units represent deposition by paleo-tsunami events. Please refer Figure 3a-b for location. [All photos taken by JNM].


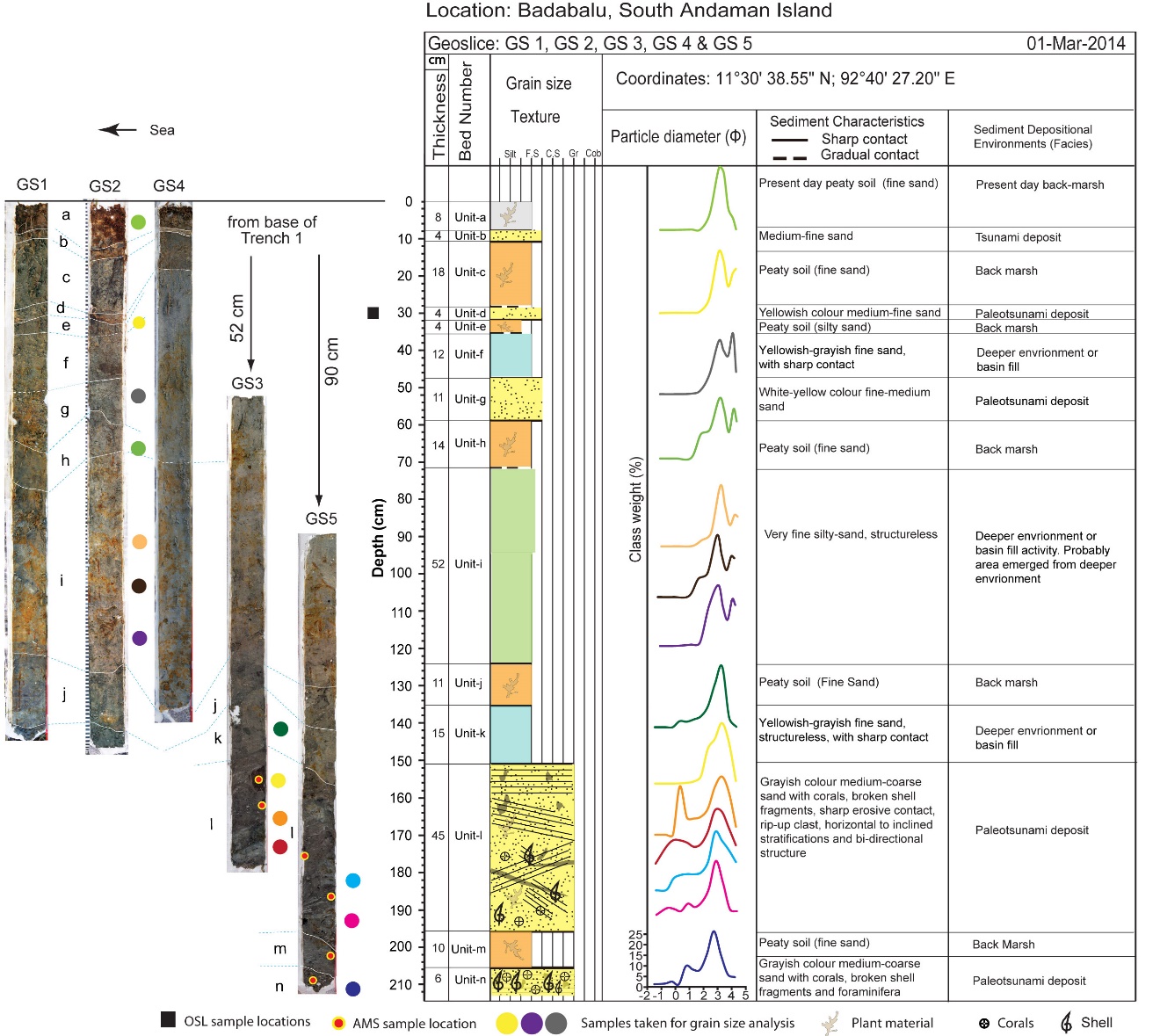


**Fig. S2.3:** Geoslice sections GS1, GS2, GS4 and GS5 along with composite log, grain size distribution, sediment characteristics and probable interpretations. These geoslice sections were collected from the back-marsh area. Please refer Figure 3a-b for location. [All photos taken by JNM].

**
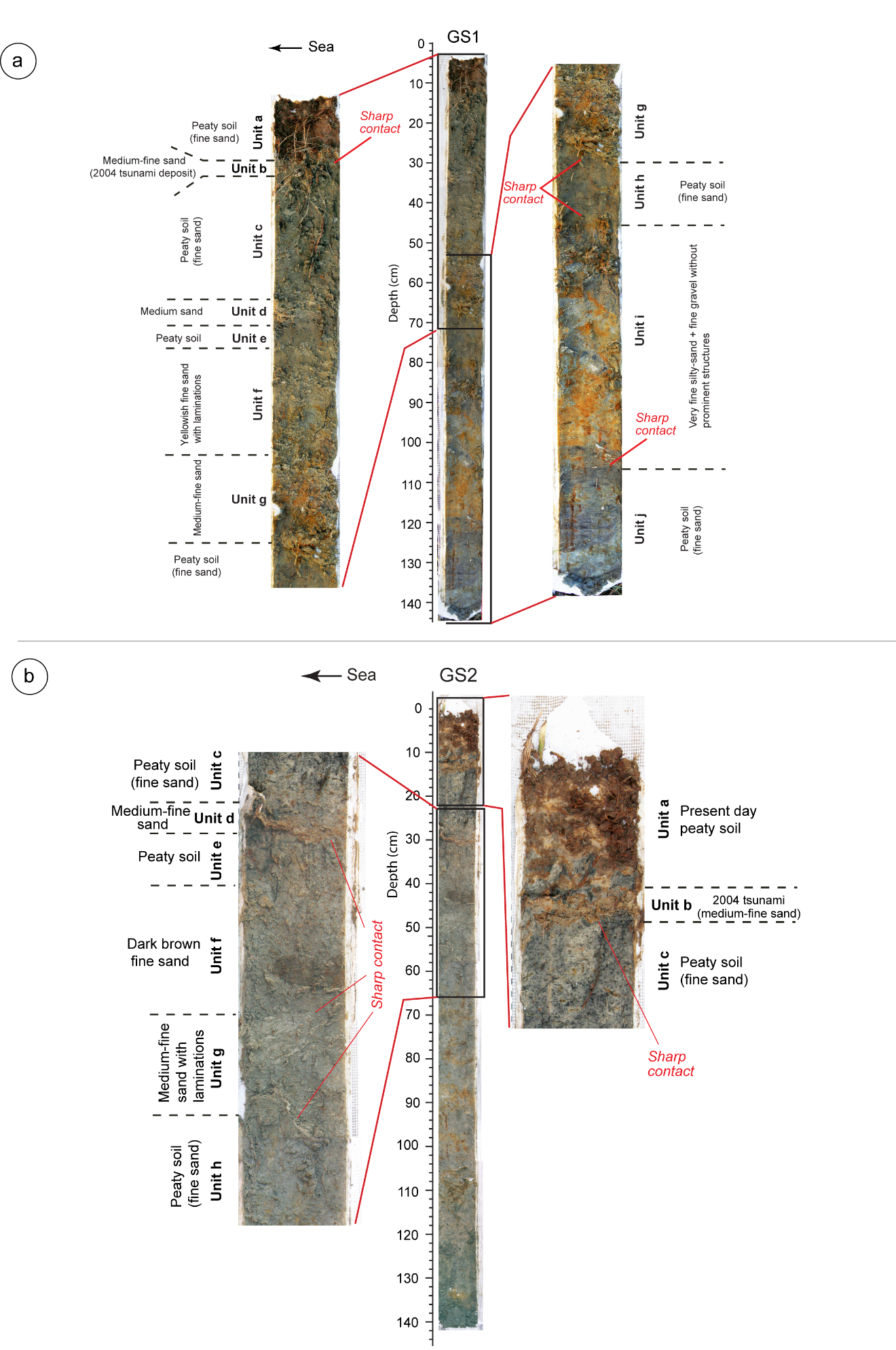
**

**Fig. S2.4:** **(a)** Close-up view of geoslice GS1. The upper portion of the stratigraphic sequence show well preserved 2004 tsunami deposit (Unit-b) with a sharp contact with underlying 2004 peaty soil (Unit-c), followed by a sand-sheets (Units d and g) suggestive of deposited during paleo-tsunami events. The middle and bottom portion show peat (Units h and j) with a sharp contact with overlying and underlying units. Geoslice section was collected from the back-marsh area. Please refer Figure 3a-b for location. **(b)** Close-up view of geoslice GS2 collected from inland showing a thin sand sheet of 2004 tsunami (Unit-b) with a sharp contact with underlying peaty unit (Unit-c). Another sand sheet Unit-g is also marked by sharp contact with the underlying and overlying units. Geoslice section was collected from the back-marsh area. Please refer Figure 3a-b for location. [All photos taken by JNM].

*(Note: Refer Fig. 3a-d for GS6, GS7, GS4 and GS3; Fig. 4a-d for GS5, GS8, GS9 and GS10).*


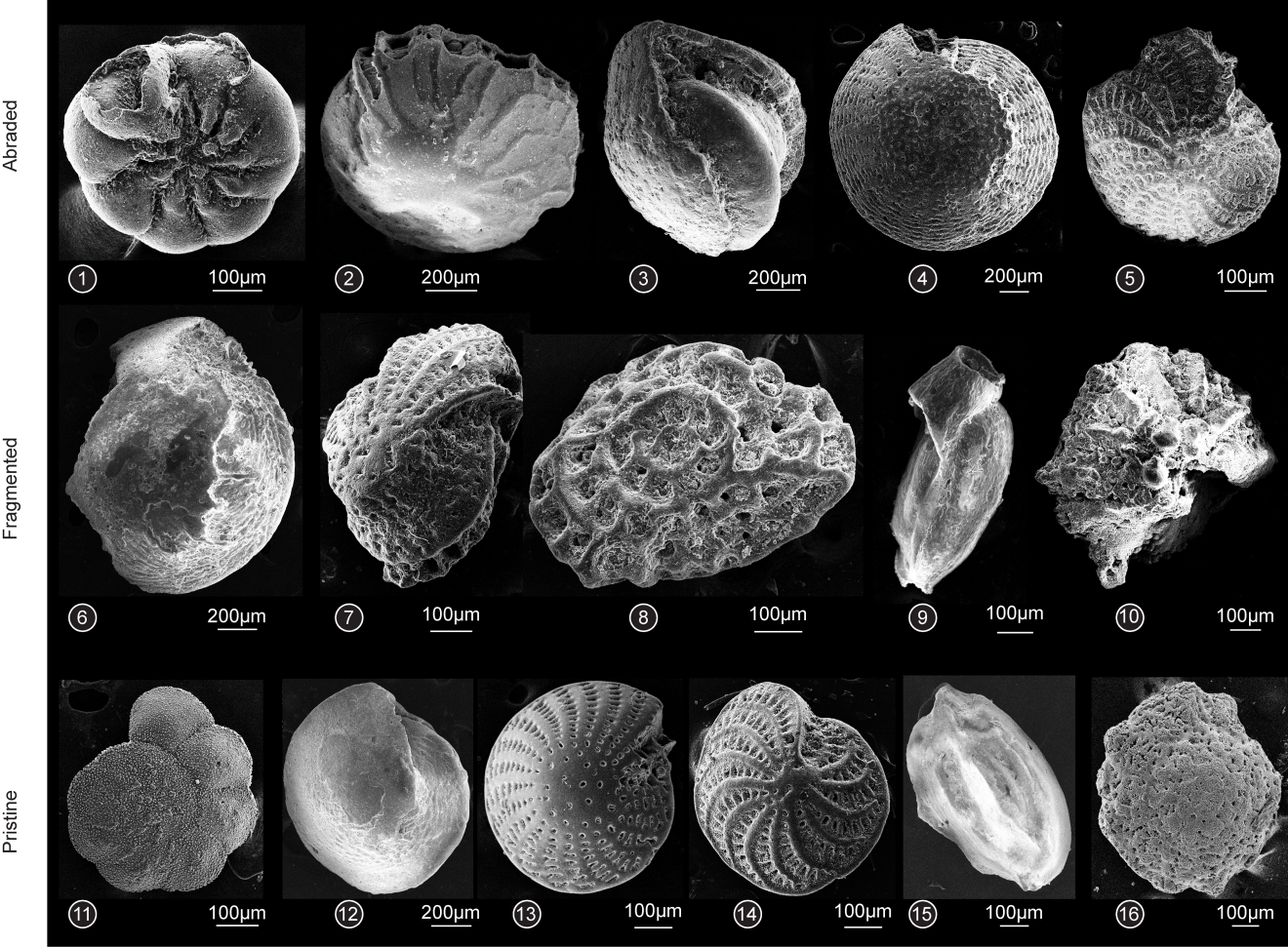


#### Fig. S3.1: The scanning electron microscope (SEM) images of Foraminifera. Taphonomic characters that describe the test condition of individual foraminifera. The first raw shows abraded tests of (1) *Ammonia beccarii* (dorsal view) (2) *Amphistegina* sp., (3) *Amphistegina lobifera,* (4) *Elphidium discoidale,* (5) *Eiphidium crispum.* The second raw shows fragmented tests of (6) *Amphistegina* sp., (7) *Eiphidium crispum,* (8) *Elphidium discoidale,* (9) *Quinqueloculina* sp. (10) *Rotalia* sp. (dorsal view). The raw three shows pristine tests of (11) *Ammonia beccarii* (ventral view) (12) *Amphistegina*sp (13) *Elphidium discoidale,* (14) *Eiphidium crispum,* (15) *Quinqueloculina seminulam,* and (16) *Rotalia* sp. (ventral view). [All photos taken with SEM at IIT Kanpur by FCJ].


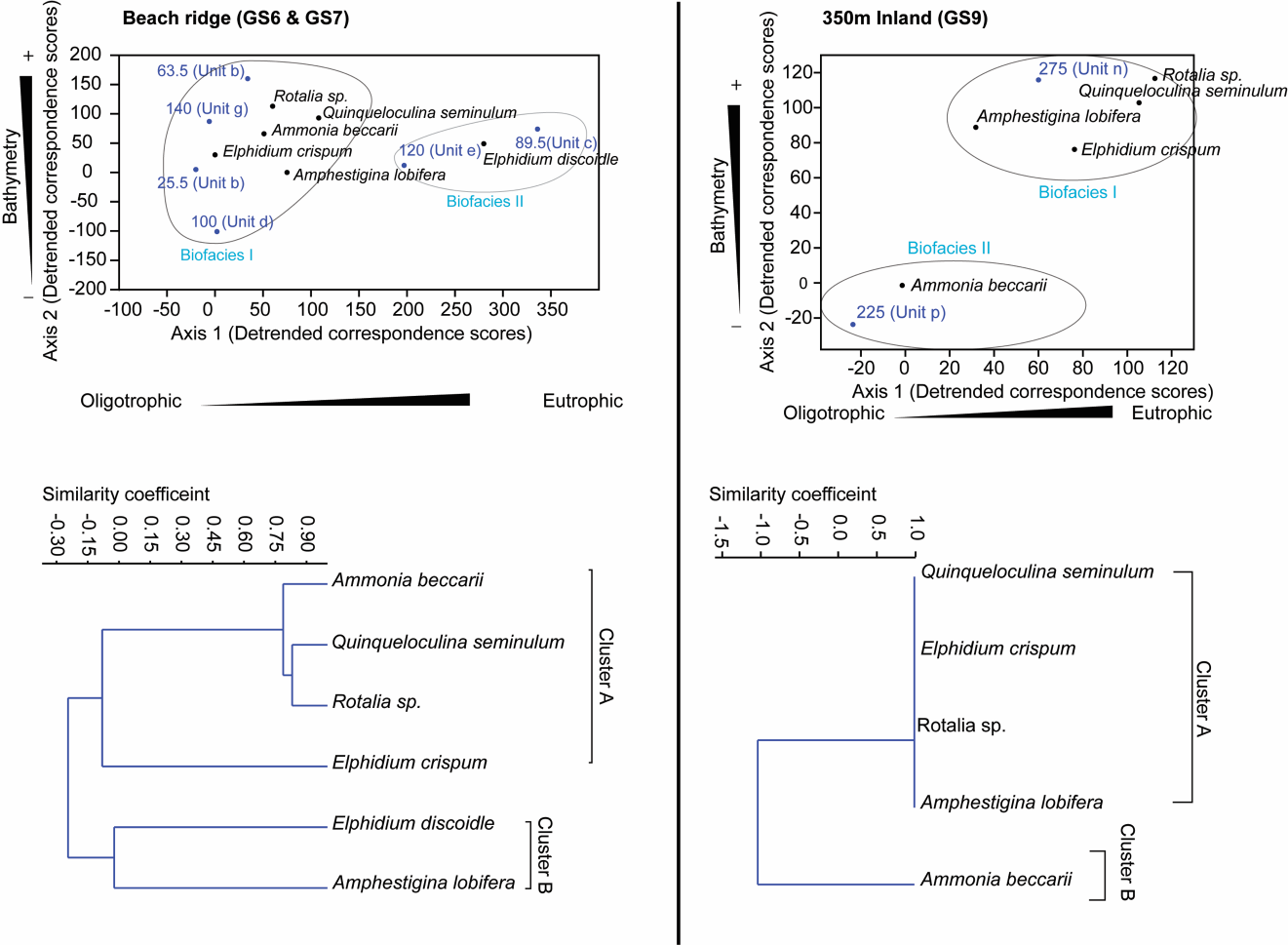


**Fig. S3.2:** Detrended correspondence diagram and Q-mode Hierarchical Dendrograms by using Paired group (UPGMA) based on correlation similarity index between the species at various depths. Biofacies-I suggests subtidal environment and sediment source. Biofacies-II suggests intertidal environment and associated sediment source.

**(a)**


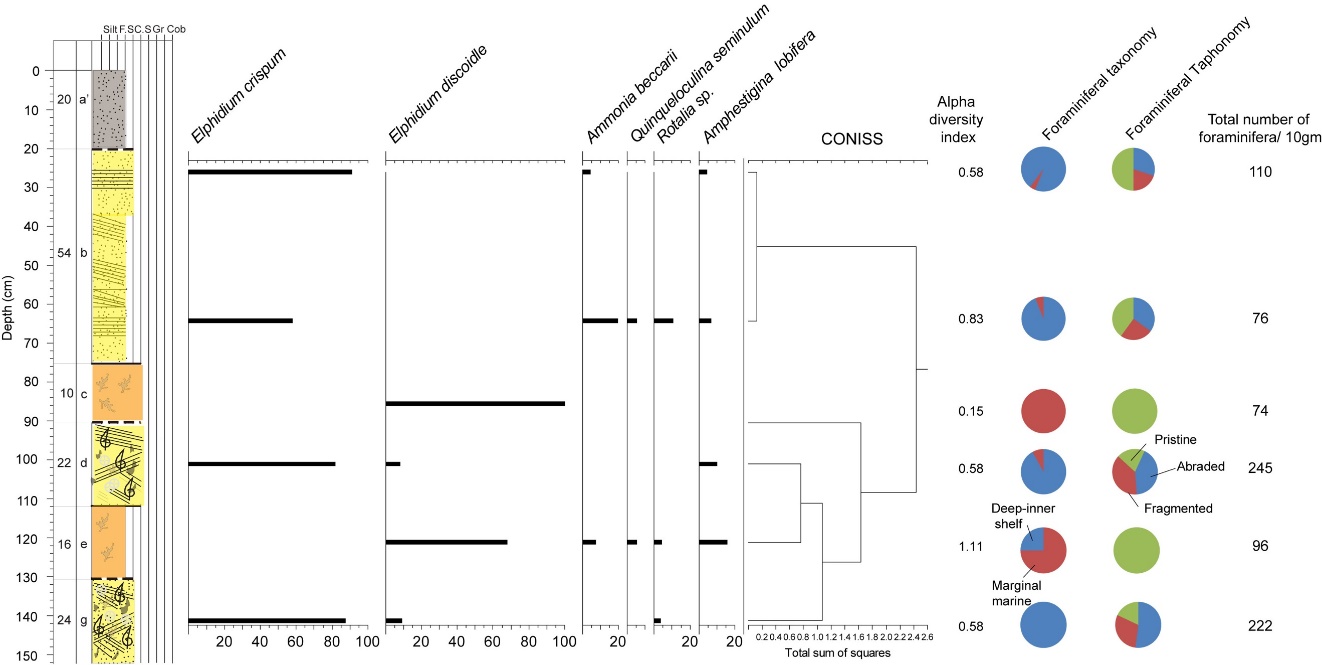


**(b)**


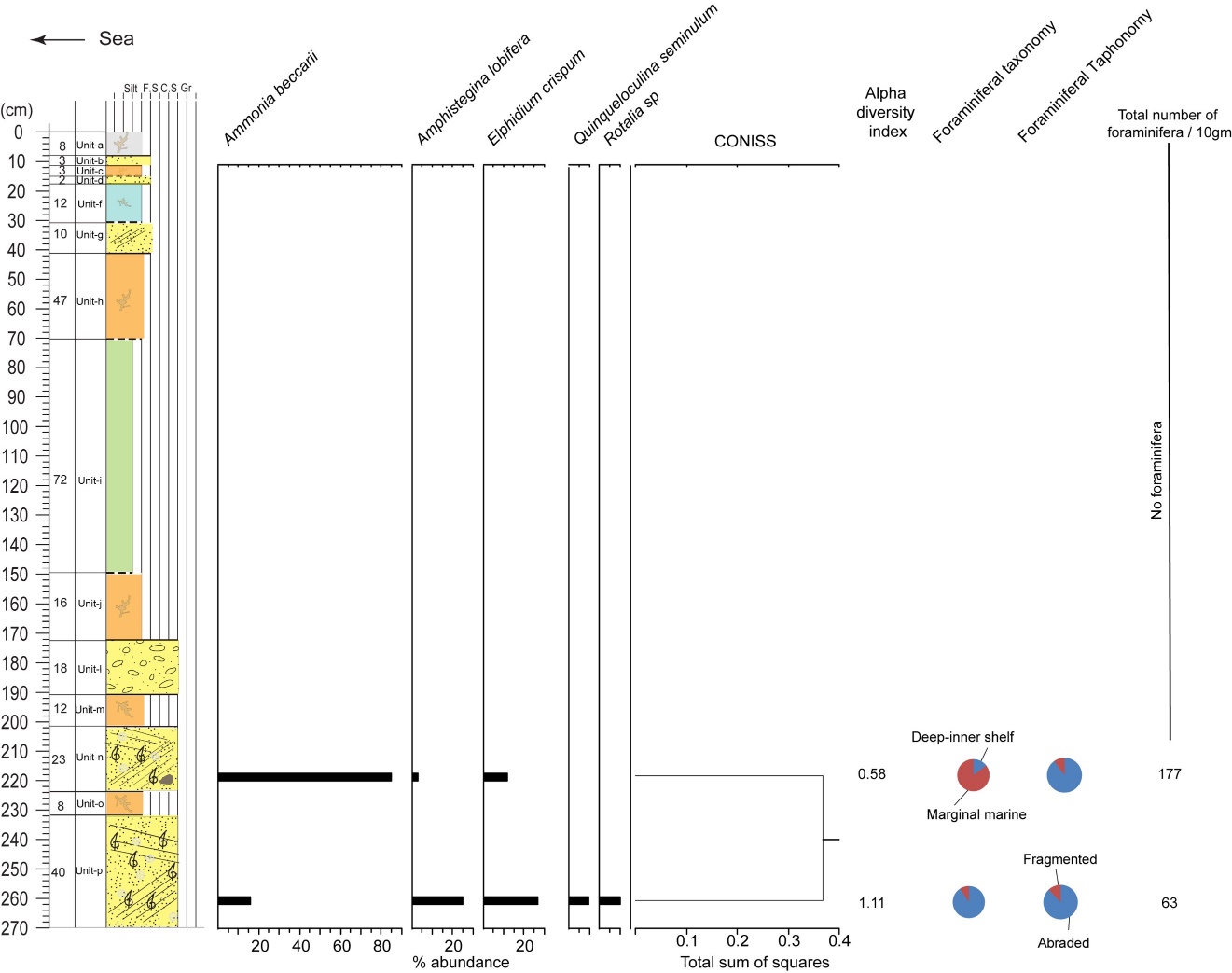


#### Fig. S3.3: (a) Geoslice sections GS6 and GS7 showing different lithounits and foraminiferid species assemblage with respect to the sediment units at various depths. (b) Geoslice GS9 showing different lithounits and foraminiferid species assemblage with respect to the sediment units at various depths.

**Fig. S4.1:** Radial Plot, Growth Curve and Histogram of the samples collected from Badabalu (viz. TSTL1; TSTL2; TS3TL1 and GS10a).

**
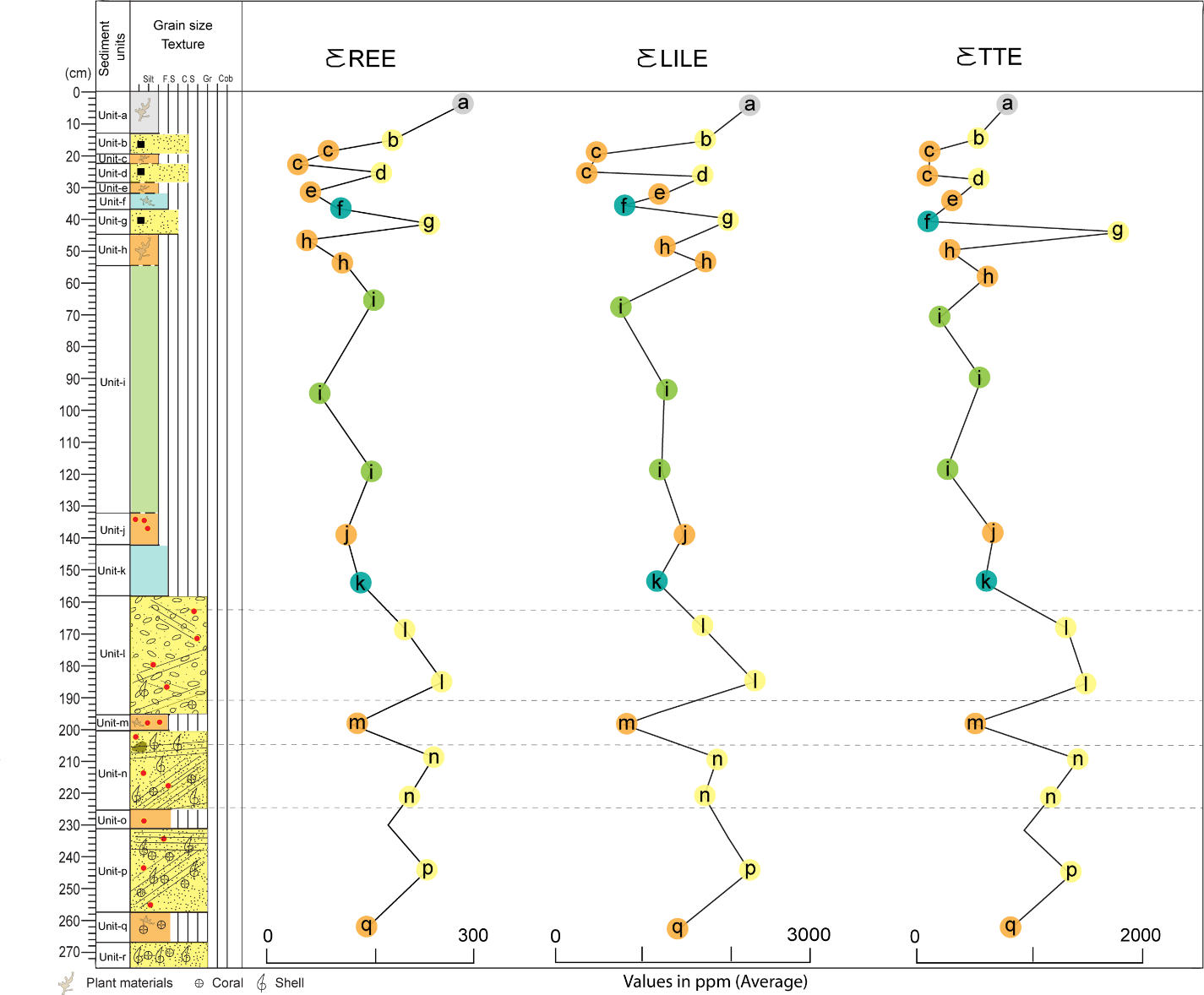
**

**Fig. S5.1:** Geochemical patterns showing variations in the total rare earth element budget (ΣREE, in ppm), Large-Ion Lithophile Elements (ΣLILE, in ppm), and Transition Trace Elements (ΣTTE, in ppm) in the complete litho-stratigraphic section (from Unit-a to Unit-q). The patterns show distinct enrichment in REE and LILE abundances in all the tsunami deposits (in yellow) except Unit-l, which shows a slightly varying composition. However, terrigenous deposits show clear depletion pattern relative to the tsunami deposits. Additionally, Units b, d, g, l, n, and p are also enriched in alkaline and transition elements, which confirm their marine origin. Transition and trace elements show remarkable enrichments in all the tsunami deposits with their highest concentration in Unit-g, and least in Units b and d. However, Transition Trace Elements (TTE) show varying compositions in all the adjacent litho-units. Lower concentrations of TTE in units c, e, f, m, and q are indicative of their terrigenous origin, however, units a, h, i, j, and k show comparatively higher concentrations, which characterize a doubtful origin might be due to inter-mixing of sediments.

**References**

1. Rajendran, K. *et al.*  Age estimates of coastal terraces in the Andaman and Nicobar Islands and their tectonic implications. *Tectonophysics* **455,** 53-60, <https://doi.org/10.1016/j.tecto.2008.05.004> (2008).
2. Rajendran, C. P., Rajendran, K., Andrade, V. & Srinivasalu, S. Ages and relative sizes of pre-2004 tsunamis in the Bay of Bengal inferred from geologic evidence in the Andaman and Nicobar Islands. *J. Geophys. Res.* **118,** 1345-1362, <https://doi.org/10.1002/jgrb.50122> (2013).
3. Rajendran, C. P. *et al.* Geoarchaeological evidence of a Chola-period tsunami from an ancient port at Kaveripattinam on the southeastern coast of India. *Geoarchaeology* **26,** 867-887, <https://doi.org/10.1002/gea.20376> (2011).
4. Malik, J. N. *et al.*  Geologic evidence for two pre-2004 earthquakes during recent centuries near Port Blair, South Andaman Island, India. *Geology* **39,** 559-562, [https://doi.org/10.1130/G31707.1](https://doi.org/10.1130/G31707.1%20) (2011).
5. Malik, J. N. *et al.* Stratigraphic evidence for earthquakes and tsunamis on the west coast of South Andaman Island, India during the past 1000 years. *Tectonophysics* **661,** 49-65, [https://doi.org/10.1016/j.tecto.2015.07.038](https://doi.org/10.1016/j.tecto.2015.07.038%20) (2015).
6. Aung, T. T. *et al.*  Geologic evidence for three great earthquakes in the past 3400 years off Myanmar. *J. Earthq. Tsunami* **02,** 259-265, [https://doi.org/10.1142/S1793431108000335](https://doi.org/10.1142/S1793431108000335%20) (2008).
7. Monecke, K. *et al.*  A 1,000-year sediment record of tsunami recurrence in northern Sumatra. *Nature* **455,** (7217), 1232, <https://doi.org/10.1038/nature07374> (2008).
8. Rubin, C. M. *et al.* Highly variable recurrence of tsunamis in the 7,400 years before the 2004 Indian Ocean tsunami. *Nat. Commun.* **8,** 16019, <https://doi.org/10.1038/ncomms16019> (2017).
9. Jankaew, K. *et al.*  Medieval forewarning of the 2004 Indian Ocean tsunami in Thailand. *Nature* **455,** 1228-1231, <https://doi.org/10.1038/nature07373> (2008).
10. Prendergast, A. L., Cupper, M. L., Jankaew, K. & Sawai, Y. Indian Ocean tsunami recurrence from optical dating of tsunami sand sheets in Thailand. *Mar. Geol.* **295–298,** 20-27, [http://dx.doi.org/10.1016/j.margeo.2011.11.012](http://dx.doi.org/10.1016/j.margeo.2011.11.012%20) (2012).
11. Fujino, S. *et al.* Stratigraphic evidence for pre-2004 tsunamis in southwestern Thailand. *Mar. Geol.* **262**, 25-28, <https://doi.org/10.1016/j.margeo.2009.02.011> (2009).
12. Meltzner, A. J. *et al.* Coral evidence for earthquake recurrence and an AD 1390–1455 cluster at the south end of the 2004 Aceh–Andaman rupture. *J. Geophys. Res.* **115**, 1-46, [https://doi.org/10.1029/2010JB007499](https://doi.org/10.1029/2010JB007499%20) (2010).
13. Meltzner, A. J. Persistent termini of 2004-and 2005-like ruptures of the Sunda megathrust. *J. Geophys. Res.* **117**, 1-15, [http://dx.doi.org/10.1029/2011JB008888](http://dx.doi.org/10.1029/2011JB008888%20) (2012).
14. Sieh, K. *et al.* Earthquake supercycles inferred from sea-level changes recorded in the Corals of west Sumatra. *Science* **322,** 1674-1677, [https://doi.org/10.1126/science.1163589](https://doi.org/10.1126/science.1163589%20) (2008).
15. Jackson, K. L. *et al.* Holocene Indian Ocean tsunami history in Sri Lanka. *Geology* **42,** 859-862, [https://doi.org/10.1130/G35796.1](https://doi.org/10.1130/G35796.1%20) (2014).
16. Dahanayake, K. & Kulasena, N. Recognition of diagnostic criteria for recent- and paleo-tsunami sediments from Sri Lanka. *Mar. Geol.* **254,** 180-186, [https://doi.org/10.1016/j.margeo.2008.06.005](https://doi.org/10.1016/j.margeo.2008.06.005%20) (2008).
17. Patton, J. R. *et al.* A 6600 year earthquake history in the region of the 2004 Sumatra- Andaman subduction zone earthquake. *Geosphere.* **11(6),** 2067-2129, https:// doi:10.1130/GES01066.1 (2015).
18. Loeblich Jr, A.R. & Tappan, H. Suprageneric classification of the Foraminiferida (Protozoa). *Micropaleontology* **30,** 1-70, [https://doi.org/10.2307/1485456](https://doi.org/10.2307/1485456%20) (1984).
19. Loeblich, A. R. & Tappan, H. *Foraminiferal Genera and Their Classification.* Von Nosttrand Reinhold Co. New York, **I-II,** 970p, 874 pls., [https://doi.org/10.1007/978-1-4899-5760-3](https://doi.org/10.1007/978-1-4899-5760-3%20) (2015).
20. Scott, D. B., Medioli, F. S. & Schafer., C. T. *Monitoring in Coastal Environments Using Foraminifera and Thecamoebian Indicators.* Cambridge University Press, 192 p., [https://doi.org/10.1086/344003](https://doi.org/10.1086/344003%20) (2001).
21. Sen Gupta, B. K. *Modern Foraminifera,* New York, Boston, Dordrecht, London, Moscow, Kluwer Academic Publishers, 371 p., <https://doi.org/10.1007/0-306-48104-9> (2007).
22. Murray, J. W. *Ecology and Applications of Benthic Foraminifera.* Cambridge University Press, 426 p., [https://doi.org/10.1017/CBO9780511535529](https://doi.org/10.1017/CBO9780511535529%20) (2006).
23. Hayward, B. W., Le Coze, F. & Gross, O. *World Foraminifera Database*. Accessed at <http://www.marinespecies.org/foraminifera> (2017).
24. Grimm, E. C. CONISS: a FORTRAN 77 program for stratigraphically constrained cluster analysis by the method of incremental sum of squares. *Computat. Geosci.***13,** 13-35, [https://doi.org/10.1016/0098-3004(87)90022-7](https://doi.org/10.1016/0098-3004(87)90022-7%20) (1987).
25. Fisher, R. A., Corbet, A. S. & Williams, C. B. The relation between the number of species and the number of individuals in a random sample of an animal population. *J. Anima.l Ecol.* 42-58, [https://doi.org/10.2307/1411](https://doi.org/10.2307/1411%20) (1943).
26. Hammer, R., Harper, D. A. T. & Ryan, P. D. PAST: Paleontological Statistics Software Package for Education and Data Analysis. *Palaeontol. Electron.* **4**, 1-9 (2001).
27. Alday, M., Cearreta, A., Freitas, M. C. & Andrade, C. Modern and late Holocene foraminiferal record of restricted environmental conditions in the Albufeira Lagoon, SW Portugal. *Geol. Acta* **11,** 75-84, <https://doi.org/10.1344/105.000001754> (2013).
28. Murray, J. W. & Alve, E. Natural dissolution of modern shallow water benthic foraminifera: taphonomic effects on the palaeoecological record. *Palaeogeogr. Palaeoclimatol. Palaeoecol.***146,** 195-209, <https://doi.org/10.1016/S0031-0182(98)00132-1> (1999).
29. Yawsangratt, S. *et al.* Evidence of probable paleotsunami deposits on Kho Khao Island, Phang Nga Province, Thailand. *Nat. Haz.* **63,** 151-163, [https://doi.org/10.1007/s11069-011-9729-4](https://doi.org/10.1007/s11069-011-9729-4%20) (2012).
30. Phleger Fred B. Foraminiferal populations and marine marsh processes. *Limnol. Oceanogr.* **15,** 522-534, [https://doi.org/10.4319/lo.1970.15.4.0522](https://doi.org/10.4319/lo.1970.15.4.0522%20) (1970).
31. Okolodkov, Y. B., Merino-Virgilio, F. D. C., Aguilar-Trujillo, A. C., Osorio-Moreno, I. & Herrera-Silveira, J. A. The genus Scrippsiella (Dinoflagellata) in coastal waters of the northern Yucatan Peninsula, SE Gulf of Mexico El género Scrippsiella (Dinoflagellata) en las aguas costeras del norte de la Península de Yucatán, sureste del Golfo de México. *Boletín de la SMF y SOFILAC* **4,** 21-32 (2014).
32. Reimer, P. J. IntCal04 terrestrial radiocarbon age calibration, 0–26 cal kyr BP. *Radiocarbon* **46,** 1029-1058, [https://doi.org/10.1017/S0033822200032999](https://doi.org/10.1017/S0033822200032999%20) (2004).
33. Reimer, P. J. *et al.* IntCal09 and Marine09 radiocarbon age calibration curves, 0-50,000 years cal BP. *Radiocarbon* **51,** 1111-1150, [https://doi.org/10.1017/S0033822200034202](https://doi.org/10.1017/S0033822200034202%20) (2009).
34. Dutta, K., Bhushan, R. & Somayajulu, B. ∆R Correction values for the northern Indian Ocean. *Radiocarbon* **43,** 483-488, [https://doi.org/10.1017/S0033822200038376](https://doi.org/10.1017/S0033822200038376%20) (2001).
35. Aitken, M. J. *An introduction to optical dating, Oxford University Press,* 267, [https://doi.org/10.2307/506799](https://doi.org/10.2307/506799%20) (1998).
36. Duller, G. A. T. Luminescence dating of Quaternary sediments: recent advances. *J. Quat. Scien.* **19,** 183–192, [https://doi.org/10.1002/jqs.809](https://doi.org/10.1002/jqs.809%20) (2004).
37. Huntley, D. J., Godfrey-Smith, D. I. & Thewalt, M. L. W. Optical dating of sediments. *Nature* **313,** 105–107, [https://doi.org/10.1038/313105a0](https://doi.org/10.1038/313105a0%20) (1985)
38. Brill, D. *et al.* OSL dating of tsunami deposits from Phra Thong Island, Thailand. *Quaternary Geochronology* **10,** 224-229, [http://dx.doi.org/10.1016/j.quageo.2012.02.016](http://dx.doi.org/10.1016/j.quageo.2012.02.016%20) (2012).
39. Cunha, P. P. *et al.* Optical dating of clastic deposits generated by an extreme marine coastal flood: The 1755 tsunami deposits in the Algarve (Portugal). *Quaternary Geochronology* **5,** 329-335, [https://doi.org/10.1016/j.quageo.2009.09.004](https://doi.org/10.1016/j.quageo.2009.09.004%20) (2010).
40. Switzer, A. D. & Jones, B. G. Large-scale washover sedimentation in a freshwater la-goon from the southeast Australian coast: sea-level change, tsunami or exception-ally large storm? *The Holocene* **18,** 787–803, [https://doi.org/10.1177/0959683608089214](https://doi.org/10.1177/0959683608089214%20) (2008).
41. Murari, M. K., Achyuthan, H. & Singhvi, A. K. Luminescence studies on the sediments laid down by the December 2004 tsunami event: prospects for the dating of palaeo tsunamis and for the estimation of sediment fluxes. *Curr. Sci.* **92,** 367–371, [https://doi.org/10.2307/24096734](https://doi.org/10.2307/24096734%20) (2007)
42. Bishop, P., Sanderson, D., Hansom, J. & Chaimanee, N. Age-dating of tsunami deposits: lessons from the 26 December 2004 tsunami in Thailand. *The Geogr. J.* **171,** 379-384, <https://doi.org/10.1111/j.1475-4959.2005.00175_4.x> (2005).
43. Robinson, R. A. J. *et al.* OSL dating and sedimentology of the Indian Ocean 2004 tsunami sediments from Thailand and the ~ 8.1 ks (Storegga) tsunami deposit in eastern Scotland. *Geophys. Res. Abst.* **10,** EGU2008-A-12382 (2008).
44. Madsen, A. T., Murray, A. S., Andersen, T. J., Pejrup, M. & Breuning-Madsen, H. Optically stimulated luminescence dating of young estuarine sediments: a comparison with ^210^Pb and ^137^Cs dating. *Mar. Geol.* **214,** 251-268, [https://doi.org/10.1016/j.margeo.2004.10.034](https://doi.org/10.1016/j.margeo.2004.10.034%20) (2005).
45. Wintle, A. G. & Murray, A. S. A review of quartz optically stimulated luminescence characteristics and their relevance in single- aliquot regeneration dating protocols. *Rad. Measur.* **41,** 369-391, [https://doi.org/10.1016/j.radmeas.2005.11.001](https://doi.org/10.1016/j.radmeas.2005.11.001%20) (2006).
46. Ballarini, M. *et al*. Optical dating of young coastal dunes on a decadal time scale. *Quat. Sci. Rev.* **22,** 1011–1017, <https://doi.org/10.1016/S0277-3791(03)00043-X> (2003).
47. Galbraith, R. F., Roberts, R. G., Laslett, G. M., Yoshida, H. & Olley, J. M. Optical dating of single and multiple grains of quartz from Jinmium rock shelter, northern Australia. Part I: Experimental design and statistical models. *Archaeometry* **41,** 339–364, [https://doi.org/10.1111/j.1475-4754.1999.tb00987.x](https://doi.org/10.1111/j.1475-4754.1999.tb00987.x%20) (1999).
48. Olley, J., Caitcheon, G. & Murray, A. The distribution of apparent dose as determined by optically stimulated luminescence in small aliquots of fluvial quartz: Implications for dating young sediments. *Quat. Sci. Rev.* **17,** 1033–1040, <https://doi.org/10.1016/S0277-3791(97)00090-5> (1998).
49. Bailey, R. M. & Arnold, L. J. Statistical modeling of single grain quartz De distributions and an assessment of procedures for estimating burial dose. *Quat. Sci. Rev.* **25**, 2475–2502, <https://doi.org/10.1016/j.quascirev.2005.09.012> (2006).
50. Ramsey, B. C., Scott, M. & van der Plicht, H. Calibration for archaeological and environmental terrestrial samples in the time range 26–50 ka cal BP. *Radiocarbon* **55**, 2021–2027, [https://doi.org/10.2458/azu_js_rc.55.16935](https://doi.org/10.2458/azu_js_rc.55.16935%20) (2013).
51. Reimer, P. J. et al. IntCal13 and Marine13 radiocarbon age calibration curves 0–50,000 years cal BP. *Radiocarbon*, **55,** 1869–1887, [https://doi.org/10.2458/azu_js_rc.55.16947](https://doi.org/10.2458/azu_js_rc.55.16947%20) (2013).
52. Srinivasalu, S. *et al.* Evaluation of trace-metal enrichments from the 26 December 2004 tsunami sediments along the southeast coast of India. *Environ. Geol.* **53,** 1711–1721, <https://doi.org/10.1007/s00254-007-0777-8> (2008).
53. Moore, A. *et al.* Sedimentary deposits from the 17 July 2006 Western Java Tsunami, Indonesia: Use of grain size analyses to assess tsunami flow depth, speed, and traction carpet characteristics. *Pur. and Appl. Geophy*. **168**, 1951–1961, <https://doi.org/10.1007/s00024-011-0280-8> (2011).
54. Font, E. *et al.* Benchmarks and sediment source(s) of the 1755 Lisbon tsunami deposit at Boca do Rio Estuary. *Mar. Geol.* **343,** 1–14, [https://doi.org/10.1016/j.margeo.2013.06.008](https://doi.org/10.1016/j.margeo.2013.06.008%20) (2013).
55. Judd, K., Goff, C. C., Goff, Gadd, J. P., Zawadzki, A. & Fierro, D. Multi-proxy evidence for small historical tsunamis leaving little or no sedimentary record. *Mar. Geol.* **385,** 204–215, [https://doi.org/10.1016/j.margeo.2017.01.002](https://doi.org/10.1016/j.margeo.2017.01.002%20) (2017).
56. Goff C. C., Szczuciński, W. & Shinozaki, T. Applications of geochemistry in tsunami research: A review. *Ear. Sci. Rev.* **165,** 203–244, <https://doi.org/10.1016/j.earscirev.2016.12.003> (2017).
57. Dahanayake, K., Kulasena, N., Ravi Prasad, G.V., Dutta, K. & Ray, D.K. Sedimentological and 14C dating studies of past tsunami events in Southern Sri Lanka. *Nat. Haz.* **63,** 197–209, [https://doi.org/10.1007/s11069-011-9824-6](https://doi.org/10.1007/s11069-011-9824-6%20) (2012).
58. Long, A. J. *et al.* Late glacial and Holocene relative sea-level changes and first evidence for the Storegga tsunami in Sutherland, Scotland. *J. Quat. Sci.* **31,** 239–255, [https://doi.org/10.1002/jqs.2862](https://doi.org/10.1002/jqs.2862%20) (2016).
59. Agah, H., Hashtroudi, M. S. & Baeyens, W. Trace Metals and Major Elements in Sediments of the Northern Persian Gulf. *J Pers. Gul. (Mar. Sci.).* **3,** 45-58, jpg.inio.ac.ir/ (2012).
60. Li, L., Huang, Z., Qiu, Q., Natawidjaya, D. H. & Sieh, K. Tsunami-induced coastal change: Scenario studies for Painan, West Sumatra, Indonesia. *Ear. Plan. Spac.* **64,** 799–816, [https://doi.org/10.5047/eps.2011.08.002](https://doi.org/10.5047/eps.2011.08.002%20) (2012).
61. Goff, C. C., Chan, J. C. H., Goff, J. & Gadd, P. Late Holocene record of environmental changes, cyclones and tsunamis in a coastal lake, Mangaia, Cook Islands. *Isl. Ar.* **25,** 333–349, <https://doi.org/10.1111/iar.12153> (2016).
62. May, S. M., Vött, A., Brückner, H. & Smedile, A. The Gyra washover fan in the Lefkada Lagoon, NW Greece - possible evidence of the 365 AD Crete earthquake and tsunami. *Eart. Planet. Spac.* **64,** 859–874, [https://doi.org/10.5047/eps.2012.03.007](https://doi.org/10.5047/eps.2012.03.007%20) (2012).
63. Koster, B., Vött, A., Mathes-Schmidt, M. & Reicherter, K. Geoscientific investigations in search of tsunami deposits in the environs of the Agoulinitsa peatland, Kaiafas Lagoon and Kakovatos (Gulf of Kyparissia, western Peloponnese, Greece). *Z. Geomorphol.* **59,** 125–156, [https://doi.org/10.1127/zfg_suppl/2014/S-00192](https://doi.org/10.1127/zfg_suppl/2014/S-00192%20) (2015).
64. Szczuciński, W. *et al.* Environmental and geological impacts of the 26 December 2004 tsunami in coastal zone of Thailand-Overview of short and long-term effects*. Pol. J. Environ. Stud.* **15,** 793–810, alnap.org/ (2006).
65. Bahlburg, H. & Weiss, R. Sedimentology of the December 26, 2004, Sumatra tsunami deposits in eastern India (Tamil Nadu) and Kenya. *Internat. J. Ear. Sci.* **96,** 1195–1209, [https://doi.org/10.1007/s00531-006-0148-9](https://doi.org/10.1007/s00531-006-0148-9%20) (2007).
66. Engel, M. *et al.* Coastal stratigraphies of eastern Bonaire (Netherlands Antilles): New insights into the palaeo-tsunami history of the southern Caribbean. *Sed. Geol.* **231,** 14–30, <https://doi.org/10.1016/j.sedgeo.2010.08.002> (2010).
67. Goff, C. C., Goff, J., Wong, H. K. Y. & Cisternas, M. Insights from geochemistry and diatoms to characterize a tsunami's deposit and maximum inundation limit. *Mar. Geol.* **359,** 22–34, [https://doi.org/10.1016/j.margeo.2014.11.009](https://doi.org/10.1016/j.margeo.2014.11.009%20) (2015).
68. Nichol, S. L. *et al.* Geomorphology and accommodation space as limiting factors on tsunami deposition: Chatham Island, southwest Pacific Ocean. *Sed. Geol.* **229,** 41–52, <https://doi.org/10.1016/j.sedgeo.2010.06.001> (2010).
69. Chandra, J., Paul, D.,  Viladkar, S.G & Sensarma, S. Origin of the Amba Dongar carbonatite complex, India and its possible linkage with the Deccan Large Igneous Province*. Geol. Soc. Lond. Spcl. Publ.* 463, 137-169, [https://doi.org/10.1144/sp463.3](https://doi.org/10.1144/sp463.3%20) (2017).
70. McDonough, W. F. & Sun, S. S. The composition of the Earth. *Chem. Geol.* **120,** 223-253, [https://doi.org/10.1016/0009-2541(94)00140-4](https://doi.org/10.1016/0009-2541(94)00140-4%20) (1995).
